# Smoking and Dental Implants: A Systematic Review and Meta-Analysis

**DOI:** 10.3390/medicina58010039

**Published:** 2021-12-27

**Authors:** Abir Dunia Mustapha, Zainab Salame, Bruno Ramos Chrcanovic

**Affiliations:** 1 Faculty of Odontology, Malmö University, 214 21 Malmo, Sweden; abirmustapha@hotmail.com (A.D.M.); zainab.salame@hotmail.com (Z.S.); 2 Department of Prosthodontics, Faculty of Odontology, Malmö University, 214 21 Malmo, Sweden

**Keywords:** dentistry, implantology, dental implant, failure, marginal bone loss, smoking, systematic review, meta-analysis, meta-regression

## Abstract

*Background and Objectives*: Tobacco is today the single most preventable cause of death, being associated with countless diseases, including cancer and neurological, cardiovascular, and respiratory diseases. Smoking also brings negative consequences to oral health, potentially impairing treatment with dental implants. The present review aimed to evaluate the influence of smoking on dental implant failure rates and marginal bone loss (MBL). *Materials and Methods*: Electronic search was undertaken in three databases, plus a manual search of journals. Meta-analyses were performed, in addition to meta-regressions, in order to verify how the odds ratio (OR) and MBL were associated with follow-up time. *Results*: The review included 292 publications. Altogether, there were 35,511 and 114,597 implants placed in smokers and in non-smokers, respectively. Pairwise meta-analysis showed that implants in smokers had a higher failure risk in comparison with non-smokers (OR 2.402, *p* < 0.001). The difference in implant failure between the groups was statistically significant in the maxilla (OR 2.910, *p* < 0.001), as well as in the mandible (OR 2.866, *p* < 0.001). The MBL mean difference (MD) between the groups was 0.580 mm (*p* < 0.001). There was an estimated decrease of 0.001 in OR (*p* = 0.566) and increase of 0.004 mm (*p* = 0.279) in the MBL MD between groups for every additional month of follow-up, although without statistical significance. Therefore, there was no clear influence of the follow-up on the effect size (OR) and on MBL MD between groups. *Conclusions*: Implants placed in smokers present a 140.2% higher risk of failure than implants placed in non-smokers.

## 1. Introduction

Tobacco is today the single most preventable cause of death, killing an estimate of more than 8 million people each year, leading many more to suffer from illnesses [1]. Smoking has been associated with countless diseases, including cancer and neurological, cardiovascular, and respiratory diseases [2,3]. Therefore, from an economics point of view, the increase in the prevalence of illnesses connected with smoking contributes to an increase in overall medical costs [2].

Tobacco use brings negative consequences to oral health [4]. Not only the prevalence, but also the severity of periodontal diseases is usually higher among smokers than among non-smokers [5]. Smoking is connected with various lesions in the oral cavity, either malignant or not, which includes black hairy tongue, leukoplakia, epithelial dysplasia, oral squamous cell carcinoma, among others [6,7]. Smoking is also associated with deleterious effects to oral rehabilitation with dental implants. Previous systematic reviews on the subject had shed some light on the issue [8,9,10,11,12,13,14]. The results suggested that placement of implants in smokers had an influence on implant failure rates and on marginal bone loss (MBL) when compared with placement in non-smokers. One review was published several years ago [11], and since then many more clinical studies looking into this matter have been published. Additionally, the other reviews [8,9,12,13,14] were based on a limited number of included studies. It was therefore the aim of the present systematic review to perform an update on the subject, adding more information from additional studies.

## 2. Materials and Methods

This study followed the PRISMA 2020 Statement guidelines [15]. Registry in PROSPERO was undertaken with the registration number CRD42021240682.

### 2.1. Objectives

The purpose of the present study was to test the null hypothesis of no difference in the implant failure rates and marginal bone loss after the insertion of dental implants in patients who smoke compared with the insertion in non-smokers, against the alternative hypothesis of a difference, based on a systematic review of the literature. The focused question was elaborated by using the PICO format (participants, interventions, comparisons, outcomes): In patients being rehabilitated with dental implants, what is the effect of smoking on the implant failure rates and marginal bone loss?

### 2.2. Search Strategies

An electronic search without time restrictions for studies published in English was undertaken and last updated in October 2021 in the following databases: PubMed/Medline, Web of Science, and Science Direct. The following terms were used in the search strategies:

(dental implant OR oral implant) AND (smoking OR smoker OR tobacco OR nicotine)

A manual search of dental implant-related journals (listed in the Supplementary Materials) was performed. The reference list of the identified studies and the relevant reviews on the subject were also checked for possible additional studies.

### 2.3. Inclusion and Exclusion Criteria

Clinical human studies were included, with information on implant failure rates in diabetic and in non-diabetic individuals, rehabilitated with cylindrical modern dental implants of commercially pure titanium or its alloys. Case reports, technical reports, animal and in vitro studies, and review papers were excluded. Studies evaluating mini-implants, zygomatic, orthodontic, zirconia, subperiosteal, or hollow implants were excluded.

### 2.4. Study Selection

The titles and abstracts of all reports identified through the electronic searches were read independently by the authors. For studies appearing to meet the inclusion criteria, or for which there were insufficient data in the title and abstract to make a clear decision, the full report was obtained. Disagreements were resolved by discussion between the authors.

RefWorks Reference Management Software version 4.6.241 (Ex Libris, Jerusalem, Israel) was used in order to detect duplicate references in different electronic databases.

### 2.5. Quality Assessment

Quality assessment of the studies was executed by the three authors of the review, according to the Quality Assessment Tool of the National Institutes of Health [16]. Studies of ‘good’ quality were judged to have at least 7 points. Disagreements were resolved by discussion between the authors.

### 2.6. Definitions

For this review, patients smoking a minimum of one cigarette per day (an everyday smoker [17]) were classified as smokers. An implant was considered a failure if presenting signs and symptoms led to implant removal, i.e., a lost implant.

### 2.7. Data Extraction

Data extraction was performed by the three authors of the review. The following data were retrieved from the studies: year of publication, country, study design, number of patients, patients’ age, implant healing period, failed and placed implants and MBL in each group, smoking definitions, implant system, jaws receiving implants (maxilla and/or mandible), and follow-up time. Contact with authors for providing missing data was performed.

### 2.8. Meta-Analysis

Implant failure (dichotomous) and MBL (continuous) were the outcomes evaluated. The statistical unit for the outcomes was the implant. The I^2^ statistic evaluated heterogeneity, and the inverse variance method was used for random-effects or fixed-effects model, depending on the heterogeneity. The estimates of relative effect for implant failure were expressed in odds ratio (OR) and in mean difference (MD) in millimeters for MBL. Meta-regressions were performed to verify how the OR and MBL were associated with the time of follow-up. The significance level was set at 0.05. The data were analyzed using OpenMeta[Analyst] version 12.11.14 (Tufts University, Boston, MA, USA) [18]. A funnel plot (plot of effect size versus standard error) was drawn, with the software OpenMEE version 04.19.16 (Tufts University, Boston, MA, USA) [19].

## 3. Results

### 3.1. Literature Search

**Figure 1:**
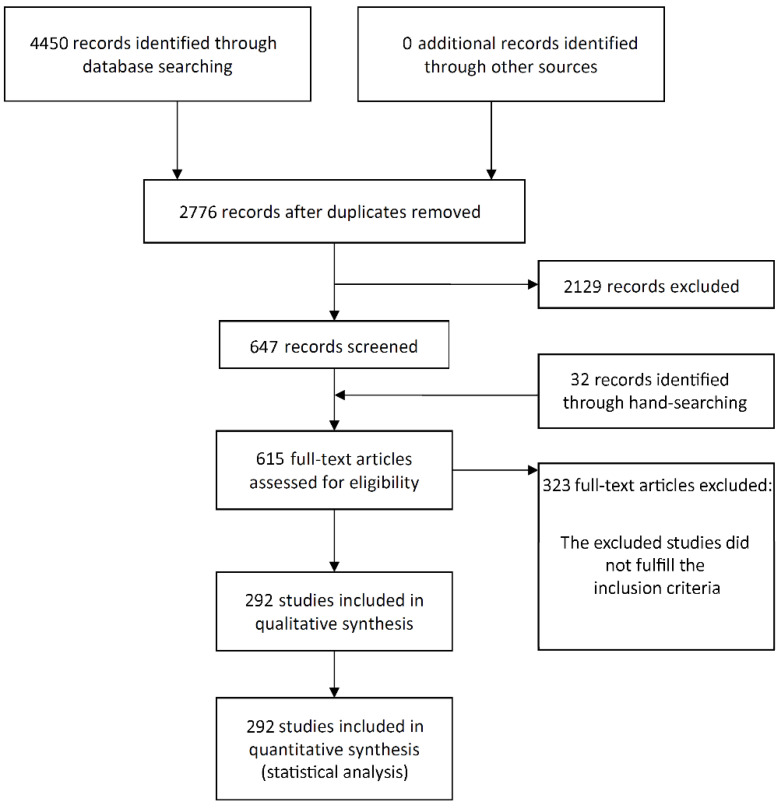
Study screening process.

The study selection process is summarized in Figure 1. The search initially resulted in 4450 papers (1454 in PubMed, 1767 in Web of Science, 1229 in ScienceDirect—in the last one the filter ‘Article type—Research articles’ was used, as well as the terms ‘dental implant’ and ‘oral implant’ between quotation marks, due to the great number of initial entries), of which 292 publications were eligible for inclusion (see Supplementary Materials for list of included articles).

### 3.2. Description of the Studies

Table S1 (see Supplementary Materials) presents detailed data of the 292 included studies. The articles were published between 1993 and 2021. A total of 231 studies were unicenter, 51 were multicenter, and it was not possible to get clear information for the other 10 studies. When it comes to study design, 54 studies were randomized clinical trials (RCT), 42 were prospective studies (without a pre-established controlled group), 22 were prospective controlled clinical trials, and were 174 retrospective observational studies. For 138 studies, at least one university was reported as the institution where the study was carried out, which was the case for private dental practice for 127 studies. Multicenter studies could include the two types of institutions—namely private practice and university. For 14 studies, it was not possible to get information on the type of institution where the study was performed. Italy was the country where the research was carried out for 76 studies (other countries could be included in case of multicenter studies). Other common places for the studies (the same observation for multicenter studies applies here) were USA in 47 cases; Spain in 23 cases; Belgium in 18 cases; Sweden, Israel, and Switzerland in 17 cases each; Germany in 15 cases; and Brazil and China in 7 cases each, among others.

The mean follow-up ± standard deviation of 257 studies was 52.7 ± 43.8 months (min–max, 3–291). For the other 35 studies, there was neither information on the precise time of follow-up nor the mean follow-up time. Information on follow-up in these 35 studies was usually reported as, for example, ‘patients were followed up between the years 2008 to 2012’, or ‘patients were followed up for up to 48 months’.

Immediate prosthetic loading of the implants was applied in 111 studies, early loading in 12 studies, and delayed loading in 169 studies. These loading protocols could be either separately (either immediate, or early, or delayed) applied for all implants of a study, or a combination of them for different implants of the same study. For 1 study, the implants were not loaded, and for 57 studies this information was not available.

Most of the studies (*n* = 192) included implants placed in the maxilla and mandible, 68 studies included patients that received implants only in maxillae, and the other 32 studies included only implants placed in mandibles. Information on the number of smokers among the patients was not available for 33 studies.

Information on implant failure was available in 289 publications (the other 3 publications provided information on MBL only). Altogether, there were 35,511 implants that were placed in smokers and 114,597 implants placed in non-smokers, and 2265 and 3827 implant failures in these groups, respectively. Implants most commonly used were from the following manufacturers: Nobel Biocare (Göteborg, Sweden) in 78 studies, Straumann (Basel, Switzerland) in 55 studies, Astra Tech (Mölndal, Sweden) in 29 studies, and Dentsply (Mannheim, Germany) in 14 studies. Information on which implant brand and/or system used was not available in 41 studies.

Mean MBL separated by the focus groups of the present review was reported in 32 studies.

### 3.3. Quality Assessment

Almost all included studies (291 out of 292) were classified as ‘good’ according to the quality assessment tool (Table S2—see Supplementary Materials). Only one study was classified as presenting a ‘fair’ quality. However, it was deemed not sufficient to invalidate its results, as the outcome information necessary for the present review (implant failure between the groups) was clearly available. In most cases, the main issues in the publications were related to statistical methods not being well-described and to the inclusion of non-consecutive patients in the studies.

### 3.4. Meta-Analyses 

A random-effects model was used to evaluate the comparison of the implant failure between the two groups, due to heterogeneity (τ^2^ = 0.156, Chi^2^ = 459.701, I^2^ = 37.351, *p* < 0.001). The pairwise meta-analysis showed implants placed in smokers had a higher risk of failure than implants placed in non-smokers, with an OR 2.402 (95% CI, 2.176, 2.652, *p* < 0.001; Figure S1—see Supplementary Materials). An OR of 2.402 implies that failures of implants placed in smokers present a 2.402 higher risk of happening than failures of implants placed in non-smokers; i.e., implants in smokers have a higher risk of failure by 140.2% in relation to implants in non-smokers.

**Figure 2:**
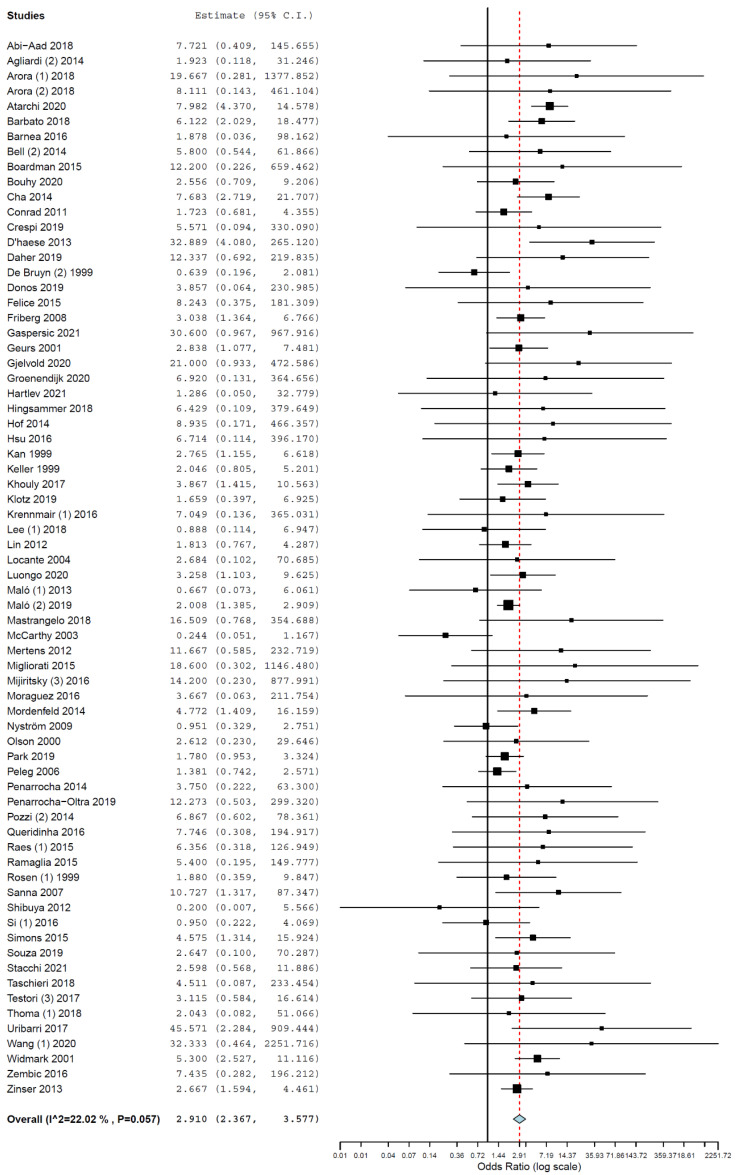
Forest plot for the event ‘implant failure’, studies evaluating implants inserted exclusively in maxillae. Estimate in odds ratio (OR).

**Figure 3:**
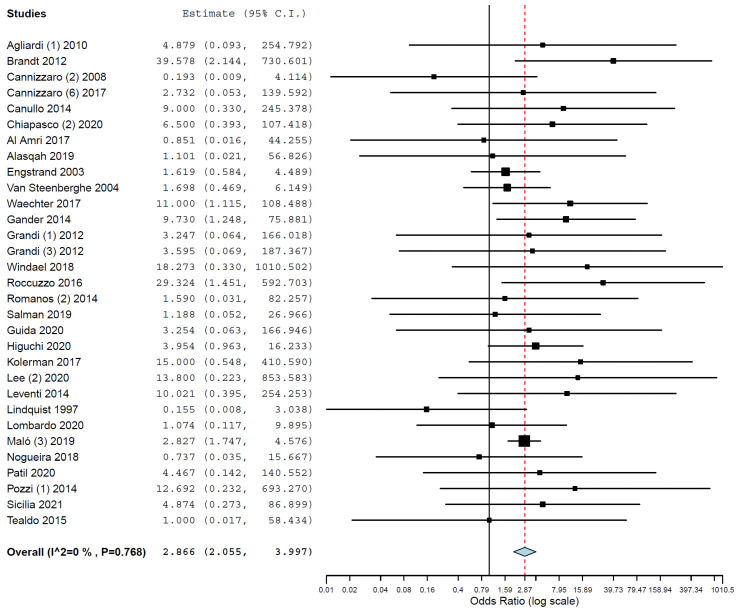
Forest plot for the event ‘implant failure’, studies evaluating implants inserted exclusively in mandibles. Estimate in odds ratio (OR).

Subgroup analyses were performed for the group of studies evaluating implants inserted exclusively in different jaws. The OR for implant failure when only studies evaluating implants inserted in maxillae were pooled was 2.910 (95% CI, 2.367, 3.577, *p* < 0.001; Figure 2), and when only studies evaluating implants inserted in mandibles were pooled was 2.866 (95% CI, 2.055, 3.997, *p* < 0.001; Figure 3).

**Figure 4:**
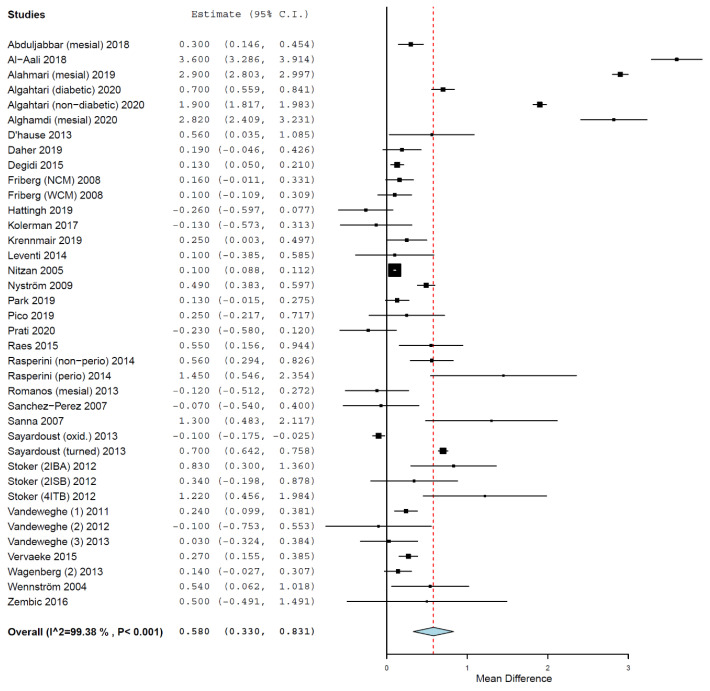
Forest plot for the event ‘marginal bone loss’. Estimate in mean difference (MD) of marginal bone loss (MBL) in millimeters.

The MD of MBL between the groups was 0.580 mm (95% CI, 0.330, 0.831, standard error = 0.128, *p* < 0.001) (τ^2^ = 0.578, Chi^2^ = 5985.613, I^2^ = 99.382, *p* < 0.001) (Figure 4), meaning that implants placed in smokers presented a mean 0.580 mm higher MBL than the implants placed in non-smokers. The difference was statistically significant.

### 3.5. Meta-Regressions

A total of 257 studies provided clear information about the follow-up time or mean follow-up time. For the other 35 studies, no precise follow-up time was possible to be obtained. Most of these studies conducted survival analysis, either life-table or Kaplan–Meier analysis, but with no mean follow-up time was provided.

When a meta-regression considering the follow-up period as a covariate in relation to OR was plotted for these 257 studies, it was observed that the follow-up time did not have an effect on the OR of implant failure between the groups. The first-degree equation resulting from the linear regression of this meta-regression was

y = 0.848 − 0.001x,

where: intercept = 0.848 (0.676, 1.021), and standard error = 0.088, *p* < 0.001. Follow-up = −0.001 (−0.003, 0.002), and standard error = 0.001, *p* = 0.566.

In this case, there was an estimated decrease of 0.001 in OR for every additional month of follow-up, although not statistically significant.

**Figure 5:**
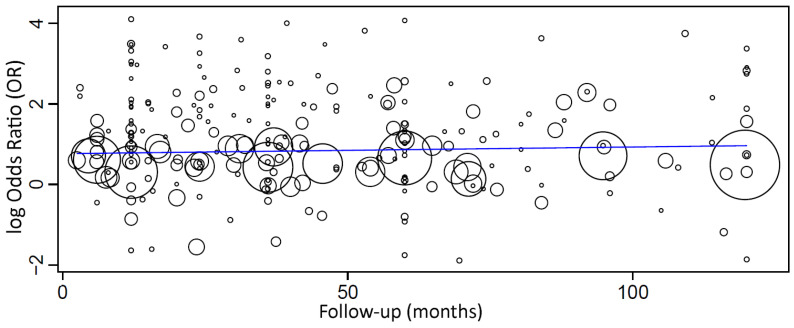
Scatter plot for the meta-regression with the association between the odds ratio (OR) of implant failure between smokers and non-smokers and the follow-up time (in months; limited to 120 months). Every circle represents a study, and the size of the circle represents the weight of the study in the analysis.

A sensitivity analysis of the meta-regression was performed, plotting together only the studies with follow-up up until 10 years (Figure 5). The first-degree equation resulting from the linear regression of this sensitivity analysis was

y = 0.764 + 0.002x,

where: intercept = 0.764 (0.545, 0.982), and standard error = 0.111, *p* < 0.001. Follow-up = 0.002 (−0.002, 0.006), and standard error = 0.002, *p* = 0.407.

A sensitivity analysis of the meta-regression was performed plotting together only the studies with follow-up up until 5 years. The first-degree equation resulting from the linear regression of this sensitivity analysis was

y = 0.698 + 0.004x,

where: intercept = 0.698 (0.432, 0.965), and standard error = 0.136, *p* < 0.001. Follow-up = 0.004 (−0.004, 0.012), and standard error = 0.004, *p* = 0.305.

However, none of these meta-regressions between follow-up and OR were statistically significant.

**Figure 6:**
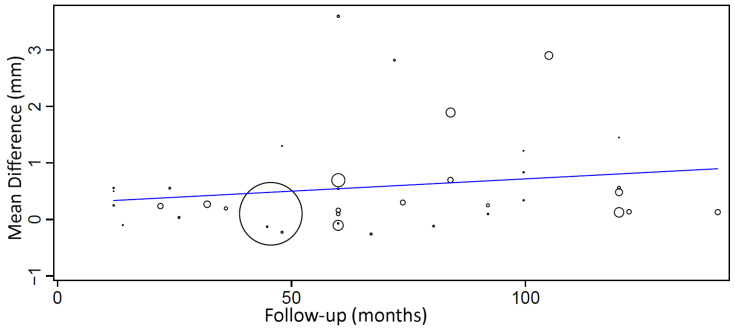
Scatter plot for the meta-regression with the association between follow-up (in months) and MBL mean difference (in millimeters) between diabetic and non-diabetic individuals. Every circle represents a study and the size of the circle represents the weight of the study in the analysis.

A meta-regression considering the effect of follow-up on MBL mean difference between groups (Figure 6) resulted in the following first-degree equation:

y = 0.283 + 0.004x,

where: intercept = 0.283 (−0.326, 0.892), and standard error = 0.311, *p* = 0.363. Follow-up = 0.004 (−0.004, 0.012), and standard error = 0.004, *p* = 0.279.

There was an estimated increase of 0.004 mm in the mean difference of MBL between groups for every additional month of follow-up, although with no statistical significance.

### 3.6. Publication Bias 

**Figure 7:**
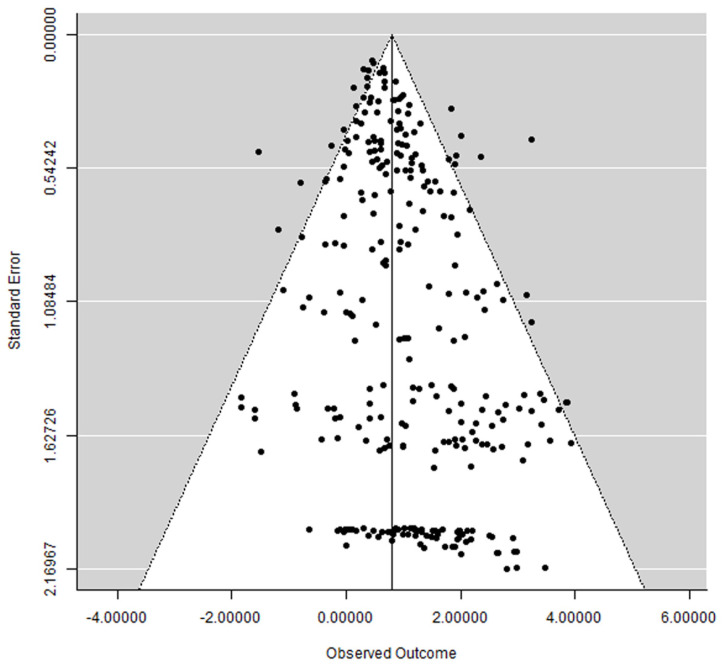
Funnel plot.

A funnel plot did not show a clear asymmetry (Figure 7), indicating the possible absence of publication bias.

## 4. Discussion

According to the results of the present review, implants placed in smokers presented a statistically significant higher risk of failure as well as a higher mean MBL than implants placed in non-smokers. The null hypothesis was therefore rejected. The meta-regression and the sensitivity analyses indicated that the effect sizes (OR) concerning failures between the groups virtually do not change with follow-up, suggesting that the effect of smoking in implant failures does not fade away with time. There are some possible explanations for the higher implant failure rate in smokers. Much is believed to be associated with the negative effects of the smoking toxins on bone metabolism and osteogenesis, and on angiogenesis, which are important in osseointegration and in the long-term maintenance of implants.

Cigarette smoke exposure causes an alteration in the composition of bone matrix and also worsens bone mineralization, which consequently leads to bone fragility. The exposure to smoke results in a reduction in bone trabeculae thickness, which is associated with a decrease in mineralizing surface as well as in the mineral deposition rate. All of this consequently leads to lower bone formation rate and longer mineralization time [20]. It has been observed that the higher the dose and the longer the duration of smoking, the higher the impact on bone mineral density [21,22]. Several pathophysiologic mechanisms that predispose smokers to bone loss have already been identified, with an inhibitory effect on osteogenesis and negative impact on bone metabolism [23]. For example, smoking has the capacity to impair the intestinal absorption of calcium by changing the metabolism of the calciotropic metabolism [24]. As another example, smoking leads to hypercortisolism [25], which changes osteoblast and osteoclast proper function [26,27]. Smokers present higher levels of free radicals [28] and increased levels oxidative stress biomarkers [29] than non-smokers, which may play an indirect role in activating bone pro-resorption pathways by affecting osteoclast differentiation and activity [30]. Smoking may also affect the so-called RANKL–RANK–OPG pathway, a series of biochemical processes that regulate the proliferation and activity of osteoclasts [31]. The process ends up disturbing the bone healing process [32].

Angiogenesis is the formation of new blood cells, which is important in the process of osseointegration of implants [33]. It has been shown that cigarette smoke inhibits several biochemical and physiological processes that disturb angiogenesis, which in turn results in abnormal blood supply to tissues, ending up decreasing repair of damaged tissues and remodeling [34,35]. Moreover, cigarette smoke was associated with decreased expression of angiogenic markers in the early bone healing phase, consequently impairing bone healing [36].

The higher MBL observed in smokers can be associated with the aforementioned negative consequences of smoking on bone metabolism, osteogenesis, and angiogenesis. There is an increased risk of peri-implantitis in smokers compared with non-smokers [37,38]. Smokers usually present worse peri-implant biological parameters than non-smokers, including higher bleeding index, deeper peri-implant pockets, and higher degree of peri-implant mucosal inflammation [39,40].

There was a statistically significant difference in the failure rate between the groups for implants placed either in the maxilla or in the mandible. Therefore, the effect smoking may be so deleterious to osseointegration and to the long-term survival of implants that its negative effects would overpower any possible advantage of the lower in the relation to the upper jaw regarding bone quality, bone volume, and cortical plates [41,42]. Previous reviews found that the impact of smoking on implant survival may be worse [43] or only significant in the maxilla [8,11], which can be associated with data from a limited number of included studies in comparison with the present review.

The present general result is similar to the results of previous reviews. The findings of four of these reviews observed significant differences in implant failure and/or MBL, with worse results for the group of smoker patients [8,9,12,13]. However, these four reviews included a very limited number of studies. Adding more information from observational studies may aid in clinical reasoning and establish a more solid foundation for causal inferences [44]. Another review focused on implants placed in areas of maxillary sinus floor augmentation, observing a statistically significant increased risk of implant failure in smokers [10]. Another review investigated the possible association between an enhanced risk of dental implant failure and an increased number of cigarettes smoked per day, observing a positive correlation between these factors [14]. As there already is considerable evidence that smoking may impair treatment with dental implants, further research should focus on the possible influence of smoking preventive measures, such as whether quitting smoking for varied lengths of time around the time of surgery may have a positive impact on the clinical outcomes and on the quantitative impact of smoking on dental implant outcomes.

## 5. Limitations of the Present Study

The results of the present study are not robust due to limitations. First of all, many included studies were retrospective clinical trials, which usually results in the absence of some important information in the publications. Second, many studies had a small sample size and/or a short follow-up period. The latter can result in an underestimation of the number of failures. Third, several studies presented a low level of specificity, meaning that their aim was not to investigate the difference in the clinical outcomes between the groups being compared in the present review. Last but not least, the studies presented many confounding factors that may also have affected the clinical outcomes of dental implants, not just the fact that implants were placed in smokers or non-smokers. As for example, we can cite the influence of implants of different diameters and lengths [45,46], status of the opposing dental arch, bruxism [47,48], diabetes [49,50], periodontal status [51,52], intake of different classes of medicaments by the patients [53,54,55,56,57], irradiation of the head and neck region [58,59], treatment performed by different professionals [60], different loading protocols [61,62], insertion in fresh extraction sockets [63], other diseases [64,65,66,67,68], type of the prosthetic configuration [69,70,71], and patient’s sex [72], among others. Moreover, individual patients sometimes present with more than one risk factor [73,74,75]. The impact of these factors is difficult to estimate if these variables are not identified separately between the different groups.

Even when journals are indexed in databases, such as the ones searched for in this review, it can still be difficult to identify all relevant studies. Although it may not be possible to be absolutely perfect in retrieving all eligible studies for a focused question, handsearching still has a valuable role to play in identifying reports of trials for inclusion in systematic reviews of health care interventions [76]. The authors of the present review tried to minimize the possibility of an incomplete retrieval of identified research by conducting a hand search of 14 dental implant-related journals and of the reference list of the identified studies and the relevant reviews on the subject, in the search for possible additional studies.

The assessment tool utilized in this systematic review indicated that almost all included studies did have a low risk of bias. Only one study was classified as presenting a moderate risk, which was due to issues that would not affect the proper eligibility of the study, such as statistical methods not well-described and inclusion of non-consecutive patients. However, this may not play an important role, as whether or not the study employed more refined statistical methods or whether or not the study well-described the statistical methods, the information necessary for the present review was still clearly available.

## 6. Conclusions

- Implants placed in smokers present a 140.2% higher risk of failure than implants placed in non-smokers;
- The difference in implant failure between the groups was statistically significant for implants placed in the maxilla and the mandible (higher for smokers);
- The mean difference in MBL between the groups was statistically significant (higher for smokers);
- There was no clear influence of the follow-up time on the effect size (OR) and on MBL mean difference between groups.

## Data Availability

The data presented in this study are available within the article and Supplementary Materials.

## Supplementary Materials

The following are available online at https://www.mdpi.com/1648-9144/58/1/39/s1, Dental implant-related journals included in the manual search, Reference list of the included articles, Figure S1: Forest plot for the event ‘implant failure’, global results—estimate in odds ratio (OR), Table S1: Detailed data of the included studies, Table S2: Quality assessment tool, according to the National Institutes of Health (NIH).
