# Supplementary material for: Smoking and Dental Implants: A Systematic Review and Meta-Analysis
Source: Medicina (Kaunas). 2021 Dec 27;58(1):39. doi: 10.3390/medicina58010039 (PMC8780868; doi:10.3390/medicina58010039)
Supplement: Supplementary file 1 [file medicina-58-00039-s001.zip › medicina-1496611-supplementary.pdf]

SUPPLEMENTARY MATERIAL

a. Dental implant-related journals included in the manual search;
b. Reference list of the included studies;
c. Figure S1. Forest plot for the event 'implant failure', global results;
d. Table S1. Detailed data of the included studies;
e. Table S2. Quality assessment of the included studies, according to the National Institutes of Health (NIH).

a. Dental implant-related journals included in the manual search

Clinical Implant Dentistry and Related Research, Clinical Oral Implants Research, European Journal of Oral Implantology, Implant Dentistry, International Journal of Implant Dentistry, International Journal of Oral and Maxillofacial Implants, International Journal of Oral Implantology, International Journal of Prosthodontics, Journal of Clinical Periodontology, Journal of Oral Implantology, Journal of Periodontology, Journal of Prosthetic Dentistry, Journal of Prosthodontics, Journal of Prosthodontic Research.

b. Reference list of the included studies

1. Abduljabbar T, Al-Hamoudi N, Al-Sowygh ZH, Alajmi M, Javed F, Vohra F. Comparison of peri-implant clinical and radiographic status around short (6 mm in length) dental implants placed in cigarette-smokers and never-smokers: Six-year follow-up results. Clin Implant Dent Relat Res. 2018 Feb;20(1):21-25.
2. Abi-Aad H, Daher F, Dimassi H, Cordioli G, Majzoub Z. Immediate vs conventional loading of variable-thread tapered implants supporting three- to four-unit fixed partial dentures in the posterior maxilla: 1-year interim results of a split-mouth randomised controlled trial. Eur J Oral Implantol. 2018;11(3):337-350.
3. Agliardi E, Clericò M, Ciancio P, Massironi D. Immediate loading of full-arch fixed prostheses supported by axial and tilted implants for the treatment of edentulous atrophic mandibles. Quintessence Int. 2010 Apr;41(4):285-93.
4. Agliardi EL, Pozzi A, Stappert CF, Benzi R, Romeo D, Gherlone E. Immediate fixed rehabilitation of the edentulous maxilla: a prospective clinical and radiological study after 3 years of loading. Clin Implant Dent Relat Res. 2014 Apr;16(2):292-302.
5. Al Amri MD, Kellesarian SV, Abduljabbar TS, Al Rifaiy MQ, Al Baker AM, Al-Kheraif AA. Comparison of Peri-Implant Soft Tissue Parameters and Crestal Bone Loss Around Immediately Loaded and Delayed Loaded Implants in Smokers and Non-Smokers: 5-Year Follow-Up Results. J Periodontol. 2017 Jan;88(1):3-9.
6. Al Najam Y, Tahmaseb A, Wiryasaputra D, Wolvius E, Dhamo B. Outcomes of dental implants in young patients with congenital versus non-congenital missing teeth. Int J Implant Dent. 2021 Aug 23;7(1):92.
7. Al-Aali KA, Alrabiah M, Al-Hamdan RS, Al-Hamoudi N, Aldahian N, Abduljabbar T. Impact of jaw location on clinical and radiological status of dental implants placed in cigarette-smokers and never-smokers: 5-year follow-up results. Clin Implant Dent Relat Res. 2018 Dec;20(6):983-987.
8. Alahmari F, Javed F, Ahmed ZU, Romanos GE, Al-Kheraif AA. Soft tissue status and crestal bone loss around conventionally-loaded dental implants placed in cigarette- and waterpipe (narghile) smokers: 8-years' follow-up results. Clin Implant Dent Relat Res. 2019 Oct;21(5):873-878.
9. Alasqah MN, Alfawaz YF, Aldahiyan N, Vohra F, Alotaibi BM, Abduljabbar T. Longitudinal assessment of clinical and radiographic periimplant status around narrow and regular diameter implants placed in cigarette-smokers and nonsmokers. Clin Implant Dent Relat Res. 2019 Oct;21(5):910-915.
10. Alghamdi O, Alrabiah M, Al-Hamoudi N, AlKindi M, Vohra F, Abduljabbar T. Peri-implant soft tissue status and crestal bone loss around immediately-loaded narrow-diameter implants placed

in cigarette-smokers: 6-year follow-up results. Clin Implant Dent Relat Res. 2020 Apr;22(2):220-225.

11. Aloy-Prósper A, Peñarrocha-Oltra D, Peñarrocha-Diago M, Camacho-Alonso F, Peñarrocha-Diago M. Peri-implant Hard and Soft Tissue Stability in Implants Placed Simultaneously Versus Delayed with Intraoral Block Bone Grafts in Horizontal Defects: A Retrospective Case Series Study. Int J Oral Maxillofac Implants. 2016 Jan-Feb;31(1):133-41.
12. Alqahtani F, Alqhtani N, Alkhtani F, Devang Divakar D, Al-Kheraif AA, Javed F. Clinicoradiographic markers of peri-implantitis in cigarette-smokers and never-smokers with type 2 diabetes mellitus at 7-years follow-up. J Periodontol. 2020 Sep;91(9):1132-1138.
13. Alsaadi G, Quirynen M, Komárek A, van Steenberghe D. Impact of local and systemic factors on the incidence of oral implant failures, up to abutment connection. J Clin Periodontol. 2007 Jul;34(7):610-7.
14. Alsaadi G, Quirynen M, Komárek A, van Steenberghe D. Impact of local and systemic factors on the incidence of late oral implant loss. Clin Oral Implants Res. 2008 Jul;19(7):670-6.
15. Alsaadi G, Quirynen M, Michiles K, Teughels W, Komárek A, van Steenberghe D. Impact of local and systemic factors on the incidence of failures up to abutment connection with modified surface oral implants. J Clin Periodontol. 2008 Jan;35(1):51-7.
16. Al-Sabbagh M, Thomas MV, Bhavsar I, De Leeuw R. Effect of Bisphosphonate and Age on Implant Failure as Determined by Patient-Reported Outcomes. J Oral Implantol. 2015 Dec;41(6):e287-91.
17. Angelis P, Passarelli PC, Gasparini G, Boniello R, D'Amato G, De Angelis S. Monolithic CAD-CAM lithium disilicate versus monolithic CAD-CAM zirconia for single implant-supported posterior crowns using a digital workflow: A 3-year cross-sectional retrospective study. J Prosthet Dent. 2020 Feb;123(2):252-256.
18. Anitua E, Alkhraisat MH. Clinical Performance of Short Dental Implants Supporting Single Crown Restoration in the Molar-Premolar Region: Cement Versus Screw Retention. Int J Oral Maxillofac Implants. 2019 July/August;34(4):969–976.
19. Anitua E, Orive G, Aguirre JJ, Ardanza B, Andía I. 5-year clinical experience with BTI dental implants: risk factors for implant failure. J Clin Periodontol. 2008 Aug;35(8):724-32.
20. Anner R, Grossmann Y, Anner Y, Levin L. Smoking, diabetes mellitus, periodontitis, and supportive periodontal treatment as factors associated with dental implant survival: a long-term retrospective evaluation of patients followed for up to 10 years. Implant Dent. 2010 Feb;19(1):57-64.
21. Arora H, Ivanovski S. Clinical and aesthetic outcomes of immediately placed single-tooth implants with immediate vs. delayed restoration in the anterior maxilla: A retrospective cohort study. Clin Oral Implants Res. 2018 Mar;29(3):346-352.
22. Arora H, Ivanovski S. Immediate and early implant placement in single-tooth gaps in the anterior maxilla: A prospective study on ridge dimensional, clinical, and aesthetic changes. Clin Oral Implants Res. 2018 Nov;29(11):1143-1154.
23. Atarchi AR, Miley DD, Omran MT, Abdulkareem AA. Early Failure Rate and Associated Risk Factors for Dental Implants Placed With and Without Maxillary Sinus Augmentation: A Retrospective Study. Int J Oral Maxillofac Implants. 2020 Nov/Dec;35(6):1187-1194.
24. Aykent F, Inan O, Ozyesil AG, Alptekin NO. A 1- to 12-year clinical evaluation of 106 endosseous implants supporting fixed and removable prostheses. Int J Periodontics Restorative Dent. 2007 Aug;27(4):358-67.
25. Bain CA, Moy PK. The association between the failure of dental implants and cigarette smoking. Int J Oral Maxillofac Implants. 1993;8(6):609-15.
26. Bain CA. Smoking and implant failure--benefits of a smoking cessation protocol. Int J Oral Maxillofac Implants. 1996 Nov-Dec;11(6):756-9.
27. Balaguer J, Ata-Ali J, Peñarrocha-Oltra D, García B, Peñarrocha-Diago M. Long-term survival rates of implants supporting overdentures. J Oral Implantol. 2015 Apr;41(2):173-7.

28. Balshi TJ, Wolfinger GJ, Slauch RW, Balshi SF. A retrospective analysis of 800 Brånemark System implants following the All-on-Four™ protocol. J Prosthodont. 2014 Feb;23(2):83-8.
29. Barbato L, Baldi N, Gonnelli A, Duvina M, Nieri M, Tonelli P. Association of Smoking Habits and Height of Residual Bone on Implant Survival and Success Rate in Lateral Sinus Lift: A Retrospective Study. J Oral Implantol. 2018 Dec;44(6):432-438.
30. Barnea E, Tal H, Nissan J, Tarrasch R, Peleg M, Kolerman R. The Use of Tilted Implant for Posterior Atrophic Maxilla. Clin Implant Dent Relat Res. 2016 Aug;18(4):788-800.
31. Barone A, Alfonsi F, Derchi G, Tonelli P, Toti P, Marchionni S, Covani U. The Effect of Insertion Torque on the Clinical Outcome of Single Implants: A Randomized Clinical Trial. Clin Implant Dent Relat Res. 2016 Jun;18(3):588-600.
32. Barone A, Toti P, Quaranta A, Derchi G, Covani U. The Clinical Outcomes of Immediate Versus Delayed Restoration Procedures on Immediate Implants: A Comparative Cohort Study for Single-Tooth Replacement. Clin Implant Dent Relat Res. 2015 Dec;17(6):1114-26.
33. Bell C, Bell RE. Immediate restoration of NobelActive implants placed into fresh extraction sites in the anterior maxilla. J Oral Implantol. 2014 Aug;40(4):455-8.
34. Bell CL, Diehl D, Bell BM, Bell RE. The immediate placement of dental implants into extraction sites with periapical lesions: a retrospective chart review. J Oral Maxillofac Surg. 2011 Jun;69(6):1623-7.
35. Bischof M, Nedir R, Abi Najm S, Szmukler-Moncler S, Samson J. A five-year life-table analysis on wide neck ITI implants with prosthetic evaluation and radiographic analysis: results from a private practice. Clin Oral Implants Res. 2006 Oct;17(5):512-20.
36. Boardman N, Darby I, Chen S. A retrospective evaluation of aesthetic outcomes for single-tooth implants in the anterior maxilla. Clin Oral Implants Res. 2016 Apr;27(4):443-51.
37. Boboeva O, Kwon TG, Kim JW, Lee ST, Choi SY. Comparing factors affecting dental-implant loss between age groups: A retrospective cohort study. Clin Implant Dent Relat Res. 2021 Apr;23(2):208-215.
38. Bouhy A, Rompen E, Lamy M, Legros C, Lecloux G, Lambert F. Maxillary implant overdenture retained by four unsplinted attachments and opposed by a natural or fixed dentition: One-year clinical outcomes. Clin Oral Implants Res. 2020 Aug;31(8):747-767.
39. Brandt R, Hollis S, Ahuja S, Adatrow P, Balanoff W. Short-term objective and subjective evaluation of small-diameter implants used to support and retain mandibular prosthesis. J Tenn Dent Assoc. 2012 Spring;92(1):34-8; quiz 38-9.
40. Cakarer S, Selvi F, Can T, Kirli I, Palancioglu A, Keskin B, Yaltirik M, Keskin C. Investigation of the risk factors associated with the survival rate of dental implants. Implant Dent. 2014 Jun;23(3):328-33.
41. Cannizzaro G, Cavallari M, Lazzarini M, Purello D'ambrosio G, Scialpi G, Audino S, Velasco-Ortega E, Ippolito DR, Esposito M. Immediate loading of three (fixed-on-3) vs four (fixed-on-4) implants supporting cross-arch fixed prostheses: 1-year results from a multicentre randomised controlled trial. Eur J Oral Implantol. 2018;11(3):323-333.
42. Cannizzaro G, Felice P, Gherlone E, Barausse C, Ferri V, Leone M, Trullenque-Eriksson A, Esposito M. Immediate loading of two (fixed-on-2) vs four (fixed-on-4) implants placed with a flapless technique supporting mandibular cross-arch fixed prostheses: 3-year results from a pilot randomised controlled trial. Eur J Oral Implantol. 2017;10(2):133-145.
43. Cannizzaro G, Felice P, Leone M, Ferri V, Viola P, Esposito M. Immediate versus early loading of 6.5 mm-long flapless-placed single implants: a 4-year after loading report of a split-mouth randomised controlled trial. Eur J Oral Implantol. 2012 Summer;5(2):111-21.
44. Cannizzaro G, Felice P, Loi I, Viola P, Ferri V, Leone M, Lazzarini M, Trullenque-Eriksson A, Esposito M. Machined versus roughened immediately loaded and finally restored single implants inserted flapless: Preliminary 6-month data from a split- mouth randomised controlled trial. Eur J Oral Implantol. 2016;9 Suppl 1(2):155-63.

45. Cannizzaro G, Felice P, Trullenque-Eriksson A, Lazzarini M, Velasco-Ortega E, Esposito M. Immediate vs early loading of 6.6 mm flapless-placed single implants: 9 years after-loading report of a split-mouth randomised controlled trial. Eur J Oral Implantol. 2018;11(2):163-173.
46. Cannizzaro G, Leone M, Esposito M. Immediate versus early loading of two implants placed with a flapless technique supporting mandibular bar-retained overdentures: a single-blinded, randomised controlled clinical trial. Eur J Oral Implantol. 2008 Spring;1(1):33-43.
47. Cannizzaro G, Leone M. Restoration of partially edentulous patients using dental implants with a microtextured surface: a prospective comparison of delayed and immediate full occlusal loading. Int J Oral Maxillofac Implants. 2003 Jul-Aug;18(4):512-22.
48. Cannizzaro G, Loi I, Viola P, Ferri V, Leone M, Trullenque-Eriksson A, Esposito M. Immediate loading of two (fixed-on-2) versus three (fixed-on-3) implants placed flapless supporting cross-arch fixed prostheses: One-year results from a randomised controlled trial. Eur J Oral Implantol. 2016;9 Suppl 1(2):143-53.
49. Canullo L, Peñarrocha D, Peñarrocha M, Rocio AG, Penarrocha-Diago M. Piezoelectric vs. conventional drilling in implant site preparation: pilot controlled randomized clinical trial with crossover design. Clin Oral Implants Res. 2014 Dec;25(12):1336-43.
50. Capparé P, Teté G, Romanos GE, Nagni M, Sannino G, Gherlone EF. The 'All-on-four' protocol in HIV-positive patients: A prospective, longitudinal 7-year clinical study. Int J Oral Implantol (Berl). 2019;12(4):501-510.
51. Carr AB, Arwani N, Lohse CM, Gonzalez RLV, Muller OM, Salinas TJ. Early Implant Failure Associated With Patient Factors, Surgical Manipulations, and Systemic Conditions. J Prosthodont. 2019 Jul;28(6):623-633.
52. Cavalcanti R, Oreglia F, Manfredonia MF, Gianserra R, Esposito M. The influence of smoking on the survival of dental implants: a 5-year pragmatic multicentre retrospective cohort study of 1727 patients. Eur J Oral Implantol. 2011 Spring;4(1):39-45.
53. Cha HS, Kim A, Nowzari H, Chang HS, Ahn KM. Simultaneous sinus lift and implant installation: prospective study of consecutive two hundred seventeen sinus lift and four hundred sixty-two implants. Clin Implant Dent Relat Res. 2014 Jun;16(3):337-47.
54. Chang LC. Risk factors associated with early failure of maxillary versus mandibular implants: A retrospective study. Int J Oral Implantol (Berl). 2020;13(1):55-63.
55. Chiapasco M, Tommasato G, Palombo D, Del Fabbro M. A retrospective 10-year mean follow-up of implants placed in ridges grafted using autogenous mandibular blocks covered with bovine bone mineral and collagen membrane. Clin Oral Implants Res. 2020 Apr;31(4):328-340.
56. Chiapasco M, Tommasato G, Palombo D, Scarnò D, Zaniboni M, Del Fabbro M. Dental implants placed in severely atrophic jaws reconstructed with autogenous calvarium, bovine bone mineral, and collagen membranes: A 3- to 19-year retrospective follow-up study. Clin Oral Implants Res. 2018 Jul;29(7):725-740.
57. Chrcanovic BR, Kisch J, Albrektsson T, Wennerberg A. A retrospective study on clinical and radiological outcomes of oral implants in patients followed up for a minimum of 20 years. Clin Implant Dent Relat Res. 2018 Apr;20(2):199-207.
58. Chrcanovic BR, Kisch J, Albrektsson T, Wennerberg A. Analysis of risk factors for cluster behavior of dental implant failures. Clin Implant Dent Relat Res. 2017 Aug;19(4):632-642.
59. Chrcanovic BR, Kisch J, Albrektsson T, Wennerberg A. Intake of Proton Pump Inhibitors Is Associated with an Increased Risk of Dental Implant Failure. Int J Oral Maxillofac Implants. 2017 September/October;32(5):1097–1102.
60. Clauser C, Sforza NM, Menini I, Kalemaj Z, Buti J; Collaborators of Accademia Toscana di Ricerca Odontostomatologica (ATRO) IPI Group. Immediate Postextraction Single-Tooth Implants and Provisional Crowns in the Esthetic Area: 2-year Results of a Cohort Prospective Multicenter Study-Patient-Centered Outcomes. Int J Oral Maxillofac Implants. 2020 Jul/Aug;35(4):833-840.

61. Conrad HJ, Jung J, Barczak M, Basu S, Seong WJ. Retrospective cohort study of the predictors of implant failure in the posterior maxilla. Int J Oral Maxillofac Implants. 2011 Jan-Feb;26(1):154-62.
62. Corvino E, Pesce P, Camodeca F, Moses O, Iannello G, Canullo L. Clinical and radiological outcomes of implants with two different connection configurations: A randomised controlled trial. Int J Oral Implantol (Berl). 2020;13(4):355-368.
63. Coskunses FM, Tak Ö. Clinical performance of narrow-diameter titanium-zirconium implants in immediately loaded fixed full-arch prostheses: a 2-year clinical study. Int J Implant Dent. 2021 Apr 16;7(1):30.
64. Crespi R, Fabris GBM, Crespi G, Toti P, Marconcini S, Covani U. Effects of different loading protocols on the bone remodeling volume of immediate maxillary single implants: A 2- to 3-year follow-up. Int J Oral Maxillofac Implants. 2019 July/August;34(4):953–962.
65. Cristalli MP, Marini R, La Monaca G, Sepe C, Tonoli F, Annibali S. Immediate loading of post-extractive single-tooth implants: a 1-year prospective study. Clin Oral Implants Res. 2015 Sep;26(9):1070-9.
66. Cucchi A, Vignudelli E, Franco S, Ghensi P, Malchiodi L, Corinaldesi G. Evaluation of Crestal Bone Loss Around Straight and Tilted Implants in Patients Rehabilitated by Immediate-Loaded Full-Arch All-on-4 or All-on-6: A Prospective Study. J Oral Implantol. 2019 Dec;45(6):434-443.
67. Daher FI, Abi-Aad HL, Dimassi HI, Cordioli G, Majzoub ZAK. Immediate versus conventional loading of variable-thread tapered implants supporting three- to four-unit fixed partial dentures in the posterior maxilla: 3-year results of a split-mouth randomised controlled trial. Int J Oral Implantol (Berl). 2019;12(4):449-466.
68. Daneshvar SS, Matthews DC, Michuad PL, Ghiabi E. Success and Survival Rates of Dental Implants Restored at an Undergraduate Dental Clinic: A 13-Year Retrospective Study with a Mean Follow-up of 5.8 Years. Int J Oral Maxillofac Implants. 2016 Jul-Aug;31(4):870-5.
69. Daubert DM, Weinstein BF, Bordin S, Leroux BG, Flemming TF. Prevalence and predictive factors for peri-implant disease and implant failure: a cross-sectional analysis. J Periodontol. 2015 Mar;86(3):337-47.
70. De Bruyn H, Collaert B, Lindén U, Johansson C, Albrektsson T. Clinical outcome of Screw Vent implants. A 7-year prospective follow-up study. Clin Oral Implants Res. 1999 Apr;10(2):139-48.
71. De Bruyn H, Collaert B. The effect of smoking on early implant failure. Clin Oral Implants Res. 1994 Dec;5(4):260-4.
72. Degidi M, Nardi D, Piattelli A. 10-year prospective cohort follow-up of immediately restored XiVE implants. Clin Oral Implants Res. 2016 Jun;27(6):694-700.
73. Deli G, Petrone V, De Risi V, Tadic D, Zafiropoulos GG. Longitudinal implant stability measurements based on resonance frequency analysis after placement in healed or regenerated bone. J Oral Implantol. 2014 Aug;40(4):438-47.
74. D'haese J, Vervaeke S, Verbanck N, De Bruyn H. Clinical and radiographic outcome of implants placed using stereolithographic guided surgery: a prospective monocenter study. Int J Oral Maxillofac Implants. 2013 Jan-Feb;28(1):205-15.
75. Di Stefano DA, Piattelli A, Zaniol T, Iezzi G. Implant and Prosthetic Success Following Peri-implant Guided Bone Regeneration in the Esthetic Zone Using an Equine Cortical Bone Membrane and an Equine Enzyme-Treated Bone Graft: A Retrospective Study with 9-year Follow-Up. Int J Oral Maxillofac Implants. 2020 Jul/Aug;35(4):824-832.
76. Doan NV, Du Z, Reher P, Xiao Y. Flapless dental implant surgery: a retrospective study of 1,241 consecutive implants. Int J Oral Maxillofac Implants. 2014 May-Jun;29(3):650-8.
77. Donati M, Ekestubbe A, Lindhe J, Wennström JL. Marginal bone loss at implants with different surface characteristics - A 20-year follow-up of a randomized controlled clinical trial. Clin Oral Implants Res. 2018 May;29(5):480-487.

78. Donati M, La Scala V, Di Raimondo R, Speroni S, Testi M, Berglundh T. Marginal bone preservation in single-tooth replacement: a 5-year prospective clinical multicenter study. Clin Implant Dent Relat Res. 2015 Jun;17(3):425-34.
79. Donos N, Horvath A, Calciolari E, Mardas N. Immediate provisionalization of bone level implants with a hydrophilic surface. A five-year follow-up of a randomized controlled clinical trial. Clin Oral Implants Res. 2019 Feb;30(2):139-149.
80. Doyle SL, Hodges JS, Pesun IJ, Baisden MK, Bowles WR. Factors affecting outcomes for single-tooth implants and endodontic restorations. J Endod. 2007 Apr;33(4):399-402.
81. Engstrand P, Gröndahl K, Ohrnell LO, Nilsson P, Nannmark U, Brånemark PI. Prospective follow-up study of 95 patients with edentulous mandibles treated according to the Brånemark Novum concept. Clin Implant Dent Relat Res. 2003;5(1):3-10.
82. Feher B, Lettner S, Heinze G, Karg F, Ulm C, Gruber R, Kuchler U. An advanced prediction model for postoperative complications and early implant failure. Clin Oral Implants Res. 2020 Oct;31(10):928-935.
83. Felice P, Pistilli R, Barausse C, Trullenque-Eriksson A, Esposito M. Immediate non-occlusal loading of immediate post-extractive versus delayed placement of single implants in preserved sockets of the anterior maxilla: 1-year post-loading outcome of a randomised controlled trial. Eur J Oral Implantol. 2015 Winter;8(4):361-72.
84. Flores-Guillen J, Álvarez-Novoa C, Barbieri G, Martín C, Sanz M. Five-year outcomes of a randomized clinical trial comparing bone-level implants with either submerged or transmucosal healing. J Clin Periodontol. 2018 Jan;45(1):125-135.
85. Fonseca M, Molinero-Mourelle P, Forrer FA, Schnider N, Hicklin SP, Schimmel M, Brägger U. Clinical performance of implant crowns with customized zirconia abutments: A prospective cohort study with a 4.5- to 8.8-year follow-up. Clin Oral Implants Res. 2021 Jul;32(7):853-862.
86. Francetti L, Azzola F, Corbella S, Taschieri S, Del Fabbro M. Evaluation of clinical outcomes and bone loss around titanium implants with oxidized surface: six-year follow-up results from a prospective case series study. Clin Implant Dent Relat Res. 2014 Feb;16(1):81-8.
87. Francetti L, Cavalli N, Taschieri S, Corbella S. Ten years follow-up retrospective study on implant survival rates and prevalence of peri-implantitis in implant-supported full-arch rehabilitations. Clin Oral Implants Res. 2019 Mar;30(3):252-260.
88. Friberg B, Jemt T. Turned Brånemark System implants in wide and narrow edentulous maxillae: a retrospective clinical study. Clin Implant Dent Relat Res. 2008 May;10(2):78-85.
89. Gamper FB, Benic GI, Sanz-Martin I, Asgeirsson AG, Hämmerle CHF, Thoma DS. Randomized controlled clinical trial comparing one-piece and two-piece dental implants supporting fixed and removable dental prostheses: 4- to 6-year observations. Clin Oral Implants Res. 2017 Dec;28(12):1553-1559.
90. Gander T, Studer S, Studer G, Grätz KW, Bredell M. Medium-term outcome of Astra Tech implants in head and neck oncology patients. Int J Oral Maxillofac Surg. 2014 Nov;43(11):1381-5.
91. Gašperšič R, Dard M, Linder S, Oblak Č. One-Year Results Assessing the Performance of Prosthetic Rehabilitations in the Posterior Maxilla Supported by 4-mm Extrashort Implants Splinted to 10-mm Implants: A Prospective Case Series. Int J Oral Maxillofac Implants. 2021 Mar-Apr;36(2):371-378.
92. Geurs NC, Wang IC, Shulman LB, Jeffcoat MK. Retrospective radiographic analysis of sinus graft and implant placement procedures from the Academy of Osseointegration Consensus Conference on Sinus Grafts. Int J Periodontics Restorative Dent. 2001 Oct;21(5):517-23.
93. Ghazal SS, Huynh-Ba G, Aghaloo T, Dibart S, Froum S, O'Neal R, Cochran D. A Randomized, Controlled, Multicenter Clinical Study Evaluating The Crestal Bone Level Change Of SLActive Bone Level Ø 3.3 mm Implants Compared To SLActive Bone Level Ø 4.1 mm Implants For Single-Tooth Replacement. Int J Oral Maxillofac Implants. 2019 May/June;34(3):708–718.

94. Gherlone EF, Capparé P, Tecco S, Polizzi E, Pantaleo G, Gastaldi G, Grusovin MG. Implant Prosthetic Rehabilitation in Controlled HIV-Positive Patients: A Prospective Longitudinal Study with 1-Year Follow-Up. Clin Implant Dent Relat Res. 2016 Aug;18(4):725-34.
95. Gjelvold B, Kisch J, Mohammed DJH, Chrcanovic BR, Albrektsson T, Wennerberg A. Immediate Loading of Single Implants, Guided Surgery, and Intraoral Scanning: A Nonrandomized Study. Int J Prosthodont. 2020 Sep/Oct;33(5):513-522.
96. Gorman LM, Lambert PM, Morris HF, Ochi S, Winkler S. The effect of smoking on implant survival at second-stage surgery: DICRG Interim Report No. 5. Dental Implant CLinical Research Group. Implant Dent. 1994 Fall;3(3):165-8.
97. Göthberg C, Gröndahl K, Omar O, Thomsen P, Slotte C. Bone and soft tissue outcomes, risk factors, and complications of implant-supported prostheses: 5-Years RCT with different abutment types and loading protocols. Clin Implant Dent Relat Res. 2018 Jun;20(3):313-321.
98. Grandi T, Guazzi P, Samarani R, Garuti G, Grandi G. Immediate loading of two unsplinted implants retaining the existing complete mandibular denture in elderly edentulous patients: 1-year results from a multicentre prospective cohort study. Eur J Oral Implantol. 2012 Spring;5(1):61-8.
99. Grandi T, Guazzi P, Samarani R, Garuti G. Immediate positioning of definitive abutments versus repeated abutment replacements in immediately loaded implants: effects on bone healing at the 1-year follow-up of a multicentre randomised controlled trial. Eur J Oral Implantol. 2012 Spring;5(1):9-16.
100. Grandi T, Guazzi P, Samarani R, Grandi G. A 3-year report from a multicentre randomised controlled trial: immediately versus early loaded implants in partially edentulous patients. Eur J Oral Implantol. 2013 Autumn;6(3):217-24.
101. Grandi T, Guazzi P, Samarani R, Grandi G. Immediate loading of four (all-on-4) post-extractive implants supporting mandibular cross-arch fixed prostheses: 18-month follow-up from a multicentre prospective cohort study. Eur J Oral Implantol. 2012 Autumn;5(3):277-85.
102. Grandi T, Guazzi P, Samarani R, Maghaireh H, Grandi G. One abutment-one time versus a provisional abutment in immediately loaded post-extractive single implants: a 1-year follow-up of a multicentre randomised controlled trial. Eur J Oral Implantol. 2014 Summer;7(2):141-9.
103. Grandi T, Guazzi P, Samarani R, Tohme H, Khoury S, Sbricoli L, Grandi G, Esposito M. Immediate, early (3 weeks) and conventional loading (4 months) of single implants: Preliminary data at 1 year after loading from a pragmatic multicenter randomised controlled trial. Eur J Oral Implantol. 2015 Summer;8(2):115-26.
104. Grisar K, Sinha D, Schoenaers J, Dormaar T, Politis C. Retrospective Analysis of Dental Implants Placed Between 2012 and 2014: Indications, Risk Factors, and Early Survival. Int J Oral Maxillofac Implants. 2017 May/June;32(3):649–654.
105. Groenendijk E, Staas TA, Bronkhorst E, Raghoebar GM, Meijer GJ. Immediate implant placement and provisionalization: Aesthetic outcome 1 year after implant placement. A prospective clinical multicenter study. Clin Implant Dent Relat Res. 2020 Apr;22(2):193-200.
106. Grossmann Y, Levin L, Sadan A. A retrospective case series of implants used to restore partially edentulous patients with implant-supported removable partial dentures: 31-month mean follow-up results. Quintessence Int. 2008 Sep;39(8):665-71.
107. Grunder U, Gaberthuel T, Boitel N, Imoberdorf M, Meyenberg K, Andreoni C, Meier T. Evaluating the clinical performance of the Osseotite implant: defining prosthetic predictability. Compend Contin Educ Dent. 1999 Jul;20(7):628-33, 636, 638-40.
108. Guarnieri R, Di Nardo D, Gaimari G, Miccoli G, Testarelli L. Short vs. Standard Laser-Microgrooved Implants Supporting Single and Splinted Crowns: A Prospective Study with 3 Years Follow-Up. J Prosthodont. 2019 Feb;28(2):e771-e779.
109. Guarnieri R, Testarelli L, Zuffetti F, Bertani P, Testori T. Comparative Results of Single Implants With and Without Laser-Microgrooved Collar Placed and Loaded with Different Protocols: A Long-Term (7 to 10 years) Retrospective Multicenter Study. Int J Oral Maxillofac Implants. 2020 Jul/Aug;35(4):841-849.

110. Guida L, Annunziata M, Esposito U, Sirignano M, Torrisi P, Cecchinato D. 6-mm-short and 11-mm-long implants compared in the full-arch rehabilitation of the edentulous mandible: A 3-year multicenter randomized controlled trial. Clin Oral Implants Res. 2020 Jan;31(1):64-73.
111. Habsha E. Survival of osseointegrated dental implants in smokers and non-smokers Toronto: University of Toronto; 2000.
112. Hakam AE, Vila G, Duarte PM, Mbadu MP, Ai Angary DS, Shuwaikan H, Aukhil I, Neiva R, da Silva HDP, Chang J. Effects of different antidepressant classes on dental implant failure: A retrospective clinical study. J Periodontol. 2021 Feb;92(2):196-204.
113. Han J, Tang Z, Zhang X, Meng H. A prospective, multi-center study assessing early loading with short implants in posterior regions. A 3-year post-loading follow-up study. Clin Implant Dent Relat Res. 2018 Feb;20(1):34-42.
114. Hartlev J, Schou S, Isidor F, Nørholt SE. A clinical and radiographic study of implants placed in autogenous bone grafts covered by either a platelet-rich fibrin membrane or deproteinised bovine bone mineral and a collagen membrane: a pilot randomised controlled clinical trial with a 2-year follow-up. Int J Implant Dent. 2021 Feb 8;7(1):8.
115. Hattingh A, De Bruyn H, Vandeweghe S. A retrospective study on ultra-wide diameter dental implants for immediate molar replacement. Clin Implant Dent Relat Res. 2019 Oct;21(5):879-887.
116. Hattingh A, Hommez G, De Bruyn H, Huyghe M, Vandeweghe S. A prospective study on ultra-wide diameter dental implants for immediate molar replacement. Clin Implant Dent Relat Res. 2018 Dec;20(6):1009-1015.
117. He J, Zhao B, Deng C, Shang D, Zhang C. Assessment of implant cumulative survival rates in sites with different bone density and related prognostic factors: an 8-year retrospective study of 2,684 implants. Int J Oral Maxillofac Implants. 2015 Mar-Apr;30(2):360-71.
118. Higuchi K, Rosenberg R, Davó R, Albanese M, Liddelow G. A Prospective Single-Cohort Multicenter Study of an Innovative Prefabricated Three-Implant-Supported Full-Arch Prosthesis for Treatment of Edentulous Mandible: 1-year Report. Int J Oral Maxillofac Implants. 2020 Jan/Feb;35(1):150-159.
119. Hingsammer L, Pommer B, Fürhauser R, Mailath-Pokorny G, Haas R, Busenlechner D. Single tooth implants in the esthetic zone following a two-stage all flapless approach: A retrospective analysis. Clin Implant Dent Relat Res. 2018 Dec;20(6):929-936.
120. Hinze M, Thalmair T, Bolz W, Wachtel H. Immediate loading of fixed provisional prostheses using four implants for the rehabilitation of the edentulous arch: a prospective clinical study. Int J Oral Maxillofac Implants. 2010 Sep-Oct;25(5):1011-8.
121. Hof M, Pommer B, Ambros H, Jesch P, Vogl S, Zechner W. Does Timing of Implant Placement Affect Implant Therapy Outcome in the Aesthetic Zone? A Clinical, Radiological, Aesthetic, and Patient-Based Evaluation. Clin Implant Dent Relat Res. 2015 Dec;17(6):1188-99.
122. Holahan CM, Koka S, Kennel KA, Weaver AL, Assad DA, Regennitter FJ, Kademani D. Effect of osteoporotic status on the survival of titanium dental implants. Int J Oral Maxillofac Implants. 2008 Sep-Oct;23(5):905-10.
123. Horwitz J, Levin L, Gabay E, Zuabi O, Machtei EE. Immediate restoration of delayed placement of dental implants in patients with treated periodontal disease: 1-year results. Int J Oral Maxillofac Implants. 2012 Nov-Dec;27(6):1569-75.
124. Hsu YT, Chan HL, Rudek I, Bashutski J, Oh WS, Wang HL, Oh TJ. Comparison of Clinical and Radiographic Outcomes of Platform-Switched Implants with a Rough Collar and Platform-Matched Implants with a Smooth Collar: A 1-Year Randomized Clinical Trial. Int J Oral Maxillofac Implants. 2016 Mar-Apr;31(2):382-90.
125. Hu C, Lang NP, Ong MM, Lim LP, Tan WC. Influence of periodontal maintenance and periodontitis susceptibility on implant success: A 5-year retrospective cohort on moderately rough surfaced implants. Clin Oral Implants Res. 2020 Aug;31(8):727-736.
126. Ibañez JC, Tahhan MJ, Zamar JA, Menendez AB, Juaneda AM, Zamar NJ, Monqaut JL. Immediate occlusal loading of double acid-etched surface titanium implants in 41 consecutive

full-arch cases in the mandible and maxilla: 6- to 74-month results. J Periodontol. 2005 Nov;76(11):1972-81.

127. Ibañez JC, Tahhan MJ, Zamar JA. Performance of double acid-etched surface external hex titanium implants in relation to one- and two-stage surgical procedures. J Periodontol. 2003 Nov;74(11):1575-81.

128. Iorio-Siciliano V, Marenzi G, Blasi A, Mignogna J, Cafiero C, Wang HL, Sammartino G. Influence of Platform-Switched, Laser-Microtextured Implant on Marginal Bone Level: A 24-Month Case Series Study. Int J Oral Maxillofac Implants. 2016 Jan-Feb;31(1):162-6.

129. Ji TJ, Kan JY, Rungcharassaeng K, Roe P, Lozada JL. Immediate loading of maxillary and mandibular implant-supported fixed complete dentures: a 1- to 10-year retrospective study. J Oral Implantol. 2012 Sep;38 Spec No:469-76.

130. Jones JD, Lupori J, Van Sickels JE, Gardner W. A 5-year comparison of hydroxyapatite-coated titanium plasma-sprayed and titanium plasma-sprayed cylinder dental implants. Oral Surg Oral Med Oral Pathol Oral Radiol Endod. 1999 Jun;87(6):649-52.

131. Kan JY, Rungcharassaeng K, Lozada JL, Goodacre CJ. Effects of smoking on implant success in grafted maxillary sinuses. J Prosthet Dent. 1999 Sep;82(3):307-11.

132. Karoussis IK, Salvi GE, Heitz-Mayfield LJ, Brägger U, Hämmerle CH, Lang NP. Long-term implant prognosis in patients with and without a history of chronic periodontitis: a 10-year prospective cohort study of the ITI Dental Implant System. Clin Oral Implants Res. 2003 Jun;14(3):329-39.

133. Keller EE, Tolman DE, Eckert SE. Maxillary antral-nasal inlay autogenous bone graft reconstruction of compromised maxilla: a 12-year retrospective study. Int J Oral Maxillofac Implants. 1999 Sep-Oct;14(5):707-21.

134. Khouly I, Pardiñas López S, Aliaga I, Froum SJ. Long-Term Implant Survival After 100 Maxillary Sinus Augmentations Using Plasma Rich in Growth Factors. Implant Dent. 2017 Apr;26(2):199-208.

135. Kim SY, Dodson TB, Do DT, Wadhwa G, Chuang SK. Factors Associated With Crestal Bone Loss Following Dental Implant Placement in a Longitudinal Follow-up Study. J Oral Implantol. 2015 Oct;41(5):579-85.

136. Kinsel RP, Liss M. Retrospective analysis of 56 edentulous dental arches restored with 344 single-stage implants using an immediate loading fixed provisional protocol: statistical predictors of implant failure. Int J Oral Maxillofac Implants. 2007 Sep-Oct;22(5):823-30.

137. Klotz AL, Ott L, Krisam J, Schmitz S, Seyidaliyeva A, Rammelsberg P, Zenthöfer A. Short-term performance of implant-supported restorations fitted in general dental practice: A retrospective study. Int J Oral Maxillofac Implants. 2019 September/October;34(5):1169–1176.

138. Kolerman R, Mijiritsky E, Barnea E, Dabaja A, Nissan J, Tal H. Esthetic Assessment of Implants Placed into Fresh Extraction Sockets for Single-Tooth Replacements Using a Flapless Approach. Clin Implant Dent Relat Res. 2017 Apr;19(2):351-364.

139. Komiyama A, Klinge B, Hultin M. Treatment outcome of immediately loaded implants installed in edentulous jaws following computer-assisted virtual treatment planning and flapless surgery. Clin Oral Implants Res. 2008 Jul;19(7):677-85.

140. Krennmair S, Hunger S, Forstner T, Malek M, Krennmair G, Stimmelmayr M. Implant health and factors affecting peri-implant marginal bone alteration for implants placed in staged maxillary sinus augmentation: A 5-year prospective study. Clin Implant Dent Relat Res. 2019 Feb;21(1):32-41.

141. Krennmair S, Weinländer M, Malek M, Forstner T, Krennmair G, Stimmelmayr M. Mandibular Full-Arch Fixed Prostheses Supported on 4 Implants with Either Axial Or Tilted Distal Implants: A 3-Year Prospective Study. Clin Implant Dent Relat Res. 2016 Dec;18(6):1119-1133.

142. Kumar A, Jaffin RA, Berman C. The effect of smoking on achieving osseointegration of surface-modified implants: a clinical report. Int J Oral Maxillofac Implants. 2002 Nov-Dec;17(6):816-9.

143. Lambert PM, Morris HF, Ochi S. The influence of smoking on 3-year clinical success of osseointegrated dental implants. Ann Periodontol. 2000 Dec;5(1):79-89.

144. Le BT, Follmar T, Borzabadi-Farahani A. Assessment of short dental implants restored with single-unit nonsplinted restorations. Implant Dent. 2013 Oct;22(5):499-502.
145. Lee CT, Sanz-Miralles E, Zhu L, Glick J, Heath A, Stoupel J. Predicting bone and soft tissue alterations of immediate implant sites in the esthetic zone using clinical parameters. Clin Implant Dent Relat Res. 2020 Jun;22(3):325-332.
146. Lee CT, Tran D, Jeng MD, Shen YT. Survival rates of hybrid rough surface implants and their alveolar bone level alterations. J Periodontol. 2018 Dec;89(12):1390-1399.
147. Leventi E, Malden NJ, Lopes VR. Periimplant bone-level reduction in relation to hydroxyapatite-coated dental implants that act as mandibular overdenture retainers: results at 6 to 10 years. J Prosthet Dent. 2014 Oct;112(4):792-7.
148. Levin L, Hertzberg R, Har-Nes S, Schwartz-Arad D. Long-term marginal bone loss around single dental implants affected by current and past smoking habits. Implant Dent. 2008 Dec;17(4):422-9.
149. Levin L, Ofec R, Grossmann Y, Anner R. Periodontal disease as a risk for dental implant failure over time: a long-term historical cohort study. J Clin Periodontol. 2011 Aug;38(8):732-7.
150. Lin TH, Chen L, Cha J, Jeffcoat M, Kao DW, Nevins M, Fiorellini JP. The effect of cigarette smoking and native bone height on dental implants placed immediately in sinuses grafted by hydraulic condensation. Int J Periodontics Restorative Dent. 2012 Jun;32(3):255-61.
151. Lindquist LW, Carlsson GE, Jemt T. Association between marginal bone loss around osseointegrated mandibular implants and smoking habits: a 10-year follow-up study. J Dent Res. 1997 Oct;76(10):1667-74.
152. Lini F, Poli PP, Beretta M, Cortinovis I, Maiorana C. Long-term retrospective observational cohort study on the survival rate of stepped screw titanium implants followed up to 20 years. Int J Oral Maxillofac Implants. 2019 July/August;34(4):999–1006.
153. Lobato RPB, Kinalski MA, Martins TM, Agostini BA, Bergoli CD, Dos Santos MBF. Influence of low-level laser therapy on implant stability in implants placed in fresh extraction sockets: A randomized clinical trial. Clin Implant Dent Relat Res. 2020 Jun;22(3):261-269.
154. Locante WM. Single-tooth replacements in the esthetic zone with an immediate function implant: a preliminary report. J Oral Implantol. 2004;30(6):369-75.
155. Lombardo G, Signoriello A, Simancas-Pallares M, Marincola M, Nocini PF. Survival of Short and Ultra-Short Locking-Taper Implants Supporting Single Crowns in the Posterior Mandible: A 3-Year Retrospective Study. J Oral Implantol. 2020 Aug 1;46(4):396-406.
156. Luongo R, Sgaramella N, Traini T, Bugea C. Graftless Maxillary Sinus Floor Augmentation with Simultaneous Porcine Bone Layer Insertion: A 1- to 5-Year Follow-up Study. Int J Oral Maxillofac Implants. 2020 Jul/Aug;35(4):808-815.
157. Malchiodi L, Balzani L, Cucchi A, Ghensi P, Nocini PF. Primary and Secondary Stability of Implants in Postextraction and Healed Sites: A Randomized Controlled Clinical Trial. Int J Oral Maxillofac Implants. 2016 Nov/Dec;31(6):1435-1443.
158. Malchiodi L, Cucchi A, Ghensi P, Consonni D, Nocini PF. Influence of crown-implant ratio on implant success rates and crestal bone levels: a 36-month follow-up prospective study. Clin Oral Implants Res. 2014 Feb;25(2):240-51.
159. Maló P, de Araújo Nobre M, Lopes A, Ferro A, Botto J. The All-on-4 treatment concept for the rehabilitation of the completely edentulous mandible: A longitudinal study with 10 to 18 years of follow-up. Clin Implant Dent Relat Res. 2019 Aug;21(4):565-577.
160. Maló P, de Araújo Nobre M, Lopes A, Ferro A, Nunes M. The All-on-4 concept for full-arch rehabilitation of the edentulous maxillae: A longitudinal study with 5-13 years of follow-up. Clin Implant Dent Relat Res. 2019 Aug;21(4):538-549.
161. Maló P, Nobre Md, Lopes A. Immediate loading of 'All-on-4' maxillary prostheses using trans-sinus tilted implants without sinus bone grafting: a retrospective study reporting the 3-year outcome. Eur J Oral Implantol. 2013 Autumn;6(3):273-83.

162. Mastrangelo F, Gastaldi G, Vinci R, Troiano G, Tettamanti L, Gherlone E, Lo Muzio L. Immediate Postextractive Implants With and Without Bone Graft: 3-year Follow-up Results From a Multicenter Controlled Randomized Trial. Implant Dent. 2018 Dec;27(6):638-645.
163. Mayfield LJ, Skoglund A, Hising P, Lang NP, Attström R. Evaluation following functional loading of titanium fixtures placed in ridges augmented by deproteinized bone mineral. A human case study. Clin Oral Implants Res. 2001 Oct;12(5):508-14.
164. McCarthy C, Patel RR, Wragg PF, Brook IM. Sinus augmentation bone grafts for the provision of dental implants: report of clinical outcome. Int J Oral Maxillofac Implants. 2003 May-Jun;18(3):377-82.
165. Mendonça JA, Francischone CE, Senna PM, Matos de Oliveira AE, Sotto-Maior BS. A retrospective evaluation of the survival rates of splinted and non-splinted short dental implants in posterior partially edentulous jaws. J Periodontol. 2014 Jun;85(6):787-94.
166. Merli M, Bianchini E, Mariotti G, Moscatelli M, Piemontese M, Rappelli G, Nieri M. Ceramic vs composite veneering of full arch implant-supported zirconium frameworks: assessing patient preference and satisfaction. A crossover double-blind randomised controlled trial. Eur J Oral Implantol. 2017;10(3):311-322.
167. Merli M, Merli M, Mariotti G, Pagliaro U, Moscatelli M, Nieri M. Immediate versus early non-occlusal loading of dental implants placed flapless in partially edentulous patients: A 10-year randomized clinical trial. J Clin Periodontol. 2020 May;47(5):621-629.
168. Mertens C, Steveling HG, Stucke K, Pretzl B, Meyer-Bäumer A. Fixed implant-retained rehabilitation of the edentulous maxilla: 11-year results of a prospective study. Clin Implant Dent Relat Res. 2012 Dec;14(6):816-27.
169. Migliorati M, Amorfini L, Signori A, Biavati AS, Benedicenti S. Clinical and Aesthetic Outcome with Post-Extractive Implants with or without Soft Tissue Augmentation: A 2-Year Randomized Clinical Trial. Clin Implant Dent Relat Res. 2015 Oct;17(5):983-95.
170. Mijiritsky E, Barbu H, Lorean A, Shohat I, Danza M, Levin L. Use of Implant-Derived Minimally Invasive Sinus Floor Elevation: A Multicenter Clinical Observational Study With 12- to 65-Month Follow-Up. J Oral Implantol. 2016 Aug;42(4):343-8.
171. Mijiritsky E, Lorean A, Mazor Z, Levin L. Implant Tooth-Supported Removable Partial Denture with at Least 15-Year Long-Term Follow-Up. Clin Implant Dent Relat Res. 2015 Oct;17(5):917-22.
172. Mijiritsky E, Mazor Z, Lorean A, Levin L. Implant diameter and length influence on survival: interim results during the first 2 years of function of implants by a single manufacturer. Implant Dent. 2013 Aug;22(4):394-8.
173. Minsk L, Polson AM, Weisgold A, Rose LF, Sanavi F, Baumgarten H, Listgarten MA. Outcome failures of endosseous implants from a clinical training center. Compend Contin Educ Dent. 1996 Sep;17(9):848-50, 852-4, 856 passim.
174. Mir-Mari J, Mir-Orfila P, Valmaseda-Castellón E, Gay-Escoda C. Long-term marginal bone loss in 217 machined-surface implants placed in 68 patients with 5 to 9 years of follow-up: a retrospective study. Int J Oral Maxillofac Implants. 2012 Sep-Oct;27(5):1163-9.
175. Moheng P, Feryn JM. Clinical and biologic factors related to oral implant failure: a 2-year follow-up study. Implant Dent. 2005 Sep;14(3):281-8.
176. Moráguez O, Vailati F, Grütter L, Sailer I, Belser UC. Four-unit fixed dental prostheses replacing the maxillary incisors supported by two narrow-diameter implants - a five-year case series. Clin Oral Implants Res. 2017 Jul;28(7):887-892.
177. Morales-Vadillo R, Leite FP, Guevara-Canales J, Netto HD, Miranda Chaves Md, Cruz F, Cruz G, Cruz-Pierce S, Cruz M. Retrospective study of the survival and associated risk factors of wedge-shaped implants. Int J Oral Maxillofac Implants. 2013 May-Jun;28(3):875-82.
178. Mordenfeld A, Albrektsson T, Hallman M. A 10-year clinical and radiographic study of implants placed after maxillary sinus floor augmentation with an 80:20 mixture of deproteinized bovine bone and autogenous bone. Clin Implant Dent Relat Res. 2014 Jun;16(3):435-46.

179. Mundt T, Mack F, Schwahn C, Biffar R. Private practice results of screw-type tapered implants: survival and evaluation of risk factors. Int J Oral Maxillofac Implants. 2006 Jul-Aug;21(4):607-14.
180. Muñoz M, Busoms E, Vilarrasa J, Albertini M, Ruíz-Magaz V, Nart J. Bone-level changes around implants with 1- or 3-mm-high abutments and their relation to crestal mucosal thickness: A 1-year randomized clinical trial. J Clin Periodontol. 2021 Oct;48(10):1302-1311.
181. Naeini EN, Dierens M, Atashkadeh M, De Bruyn H. Long-term clinical outcome of single implants inserted flaplessly or conventionally. Clin Implant Dent Relat Res. 2018 Oct;20(5):829-837.
182. Nedir R, Bischof M, Briaux JM, Beyer S, Szmukler-Moncler S, Bernard JP. A 7-year life table analysis from a prospective study on ITI implants with special emphasis on the use of short implants. Results from a private practice. Clin Oral Implants Res. 2004 Apr;15(2):150-7.
183. Niedermaier R, Stelzle F, Riemann M, Bolz W, Schuh P, Wachtel H. Implant-Supported Immediately Loaded Fixed Full-Arch Dentures: Evaluation of Implant Survival Rates in a Case Cohort of up to 7 Years. Clin Implant Dent Relat Res. 2017 Feb;19(1):4-19.
184. Nitzan D, Mamlider A, Levin L, Schwartz-Arad D. Impact of smoking on marginal bone loss. Int J Oral Maxillofac Implants. 2005 Jul-Aug;20(4):605-9.
185. Noelken R, Pausch T, Wagner W, Al-Nawas B. Peri-implant defect grafting with autogenous bone or bone graft material in immediate implant placement in molar extraction sites-1- to 3-year results of a prospective randomized study. Clin Oral Implants Res. 2020 Nov;31(11):1138-1148.
186. Nogueira TE, Aguiar FMO, de Barcelos BA, Leles CR. A 2-year prospective study of single-implant mandibular overdentures: Patient-reported outcomes and prosthodontic events. Clin Oral Implants Res. 2018 Jun;29(6):541-550.
187. Noguerol B, Muñoz R, Mesa F, de Dios Luna J, O'Valle F. Early implant failure. Prognostic capacity of Periotest: retrospective study of a large sample. Clin Oral Implants Res. 2006 Aug;17(4):459-64.
188. Norton MR. The Influence of Low Insertion Torque on Primary Stability, Implant Survival, and Maintenance of Marginal Bone Levels: A Closed-Cohort Prospective Study. Int J Oral Maxillofac Implants. 2017 Jul/Aug;32(4):849-857.
189. Nyström E, Nilson H, Gunne J, Lundgren S. Reconstruction of the atrophic maxilla with interpositional bone grafting/Le Fort I osteotomy and endosteal implants: a 11-16 year follow-up. Int J Oral Maxillofac Surg. 2009 Jan;38(1):1-6.
190. Oliva J, Oliva X, Oliva JD. All-on-three delayed implant loading concept for the completely edentulous maxilla and mandible: a retrospective 5-year follow-up study. Int J Oral Maxillofac Implants. 2012 Nov-Dec;27(6):1584-92.
191. Olmedo-Gaya MV, Manzano-Moreno FJ, Cañaveral-Cavero E, de Dios Luna-del Castillo J, Vallecillo-Capilla M. Risk factors associated with early implant failure: A 5-year retrospective clinical study. J Prosthet Dent. 2016 Feb;115(2):150-5.
192. Olson JW, Dent CD, Morris HF, Ochi S. Long-term assessment (5 to 71 months) of endosseous dental implants placed in the augmented maxillary sinus. Ann Periodontol. 2000 Dec;5(1):152-6.
193. Omran MT, Miley DD, McLeod DE, Garcia MN. Retrospective assessment of survival rate for short endosseous dental implants. Implant Dent. 2015 Apr;24(2):185-91.
194. Park WB, Kang KL, Han JY. Factors influencing long-term survival rates of implants placed simultaneously with lateral maxillary sinus floor augmentation: A 6- to 20-year retrospective study. Clin Oral Implants Res. 2019 Oct;30(10):977-988.
195. Patil PG, Seow LL. Crestal bone-level changes and patient satisfaction with mandibular overdentures retained by one or two implants with immediate loading protocols: A randomized controlled clinical study. J Prosthet Dent. 2020 May;123(5):710-716.
196. Peleg M, Garg AK, Mazor Z. Healing in smokers versus nonsmokers: survival rates for sinus floor augmentation with simultaneous implant placement. Int J Oral Maxillofac Implants. 2006 Jul-Aug;21(4):551-9.

197. Peñarrocha D, Candel E, Guirado JL, Canullo L, Peñarrocha M. Implants placed in the nasopalatine canal to rehabilitate severely atrophic maxillae: a retrospective study with long follow-up. J Oral Implantol. 2014 Dec;40(6):699-706.
198. Peñarrocha-Oltra D, Peñarrocha-Diago M, Encinas RF, Ippolito DR, Xhanari E, Esposito M. Natural or palatal positioning of immediate post-extractive implants in the aesthetic zone? Three-year results of a multicentre randomised controlled trial. Int J Oral Implantol (Berl). 2019;12(2):181-194.
199. Perez A, Caiazzo A, Valente NA, Toti P, Alfonsi F, Barone A. Standard vs customized healing abutments with simultaneous bone grafting for tissue changes around immediate implants. 1-year outcomes from a randomized clinical trial. Clin Implant Dent Relat Res. 2020 Feb;22(1):42-53.
200. Pico A, Martín-Lancharro P, Caneiro L, Nóvoa L, Batalla P, Blanco J. Influence of abutment height and implant depth position on interproximal peri-implant bone in sites with thin mucosa: A 1-year randomized clinical trial. Clin Oral Implants Res. 2019 Jul;30(7):595-602.
201. Pozzi A, Holst S, Fabbri G, Tallarico M. Clinical reliability of CAD/CAM cross-arch zirconia bridges on immediately loaded implants placed with computer-assisted/template-guided surgery: a retrospective study with a follow-up between 3 and 5 years. Clin Implant Dent Relat Res. 2015 Jan;17 Suppl 1:e86-96.
202. Pozzi A, Moy PK. Minimally invasive transcrestal guided sinus lift (TGSL): a clinical prospective proof-of-concept cohort study up to 52 months. Clin Implant Dent Relat Res. 2014 Aug;16(4):582-93.
203. Pozzi A, Tallarico M, Moy PK. Four-implant overdenture fully supported by a CAD-CAM titanium bar: A single-cohort prospective 1-year preliminary study. J Prosthet Dent. 2016 Oct;116(4):516-523.
204. Pozzi A, Tallarico M, Moy PK. Three-year post-loading results of a randomised, controlled, split-mouth trial comparing implants with different prosthetic interfaces and design in partially posterior edentulous mandibles. Eur J Oral Implantol. 2014 Spring;7(1):47-61.
205. Prati C, Zamparini F, Pirani C, Montebugnoli L, Canullo L, Gandolfi MG. A Multilevel Analysis of Platform-Switching Flapless Implants Placed at Tissue Level: 4-year Prospective Cohort Study. Int J Oral Maxillofac Implants. 2020 Mar/Apr;35(2):330-341.
206. Queridinha BM, Almeida RF, Felino A, de Araújo Nobre M, Maló P. Partial Rehabilitation with Distally Tilted and Straight Implants in the Posterior Maxilla with Immediate Loading Protocol: A Retrospective Cohort Study with 5-Year Follow-up. Int J Oral Maxillofac Implants. 2016 Jul-Aug;31(4):891-9.
207. Raabe C, Monje A, Abou-Ayash S, Buser D, von Arx T, Chappuis V. Long-term effectiveness of 6 mm micro-rough implants in various indications: A 4.6- to 18.2-year retrospective study. Clin Oral Implants Res. 2021 Aug;32(8):1008-1018.
208. Raes F, Eccellente T, Lenzi C, Ortolani M, Luongo G, Mangano C, Mangano F. Immediate functional loading of single implants: a multicenter study with 4 years of follow-up. J Dent Res Dent Clin Dent Prospects. 2018 Winter;12(1):26-37.
209. Raes S, Rocci A, Raes F, Cooper L, De Bruyn H, Cosyn J. A prospective cohort study on the impact of smoking on soft tissue alterations around single implants. Clin Oral Implants Res. 2015 Sep;26(9):1086-90.
210. Ramaglia L, Sbordone C, Saviano R, Martuscelli R, Sbordone L. Marginal masticatory mucosa dimensional changes in immediate post-extractive implants: a 2 year prospective cohort study. Clin Oral Implants Res. 2015 Dec;26(12):1495-502.
211. Rasperini G, Siciliano VI, Cafiero C, Salvi GE, Blasi A, Aglietta M. Crestal bone changes at teeth and implants in periodontally healthy and periodontally compromised patients. A 10-year comparative case-series study. J Periodontol. 2014 Jun;85(6):e152-9.
212. Ravidà A, Barootchi S, Tattan M, Saleh MHA, Gargallo-Albiol J, Wang HL. Clinical outcomes and cost effectiveness of computer-guided versus conventional implant-retained hybrid prostheses:

A long-term retrospective analysis of treatment protocols. J Periodontol. 2018 Sep;89(9):1015-1024.
213. Ravidà A, Tattan M, Askar H, Barootchi S, Tavelli L, Wang HL. Comparison of three different types of implant-supported fixed dental prostheses: A long-term retrospective study of clinical outcomes and cost-effectiveness. Clin Oral Implants Res. 2019 Apr;30(4):295-305.
214. Roccuzzo M, Grasso G, Dalmasso P. Keratinized mucosa around implants in partially edentulous posterior mandible: 10-year results of a prospective comparative study. Clin Oral Implants Res. 2016 Apr;27(4):491-6.
215. Rodriguez-Argueta OF, Figueiredo R, Valmaseda-Castellon E, Gay-Escoda C. Postoperative complications in smoking patients treated with implants: a retrospective study. J Oral Maxillofac Surg. 2011 Aug;69(8):2152-7.
216. Romandini M, Cordaro M, Donno S, Cordaro L. Discrepancy between patient satisfaction and biologic complication rate in patients rehabilitated with overdentures and not participating in a structured maintenance program after 7 to 12 years of loading. Int J Oral Maxillofac Implants. 2019 September/October;34(5):1143–1151.
217. Romanos GE, Gaertner K, Aydin E, Nentwig GH. Long-term results after immediate loading of platform-switched implants in smokers versus nonsmokers with full-arch restorations. Int J Oral Maxillofac Implants. 2013 May-Jun;28(3):841-5.
218. Romanos GE, Gaertner K, Nentwig GH. Long-term evaluation of immediately loaded implants in the edentulous mandible using fixed bridges and platform shifting. Clin Implant Dent Relat Res. 2014 Aug;16(4):601-8.
219. Rosen PS, Sahlin H, Seemann R, Rosen AS. A 1-7 year retrospective follow-up on consecutively placed 7-mm-long dental implants with an electrowetted surface. Int J Implant Dent. 2018 Aug 23;4(1):24.
220. Rosen PS, Summers R, Mellado JR, Salkin LM, Shanaman RH, Marks MH, Fugazzotto PA. The bone-added osteotome sinus floor elevation technique: multicenter retrospective report of consecutively treated patients. Int J Oral Maxillofac Implants. 1999 Nov-Dec;14(6):853-8.
221. Rossi F, Lang NP, Ricci E, Ferraioli L, Baldi N, Botticelli D. Long-term follow-up of single crowns supported by short, moderately rough implants-A prospective 10-year cohort study. Clin Oral Implants Res. 2018 Dec;29(12):1212-1219.
222. Rossi F, Lang NP, Ricci E, Ferraioli L, Marchetti C, Botticelli D. 6-mm-long implants loaded with fiber-reinforced composite resin-bonded fixed prostheses (FRCRBFDPs). A 5-year prospective study. Clin Oral Implants Res. 2017 Dec;28(12):1478-1483.
223. Salman A, Thacker S, Rubin S, Dhingra A, Ioannidou E, Schincaglia GP. Immediate versus delayed loading of mandibular implant-retained overdentures: A 60-month follow-up of a randomized clinical trial. J Clin Periodontol. 2019 Aug;46(8):863-871.
224. Sánchez-Pérez A, Moya-Villaescusa MJ, Caffesse RG. Tobacco as a risk factor for survival of dental implants. J Periodontol. 2007 Feb;78(2):351-9.
225. Sanna AM, Molly L, van Steenberghe D. Immediately loaded CAD-CAM manufactured fixed complete dentures using flapless implant placement procedures: a cohort study of consecutive patients. J Prosthet Dent. 2007 Jun;97(6):331-9.
226. Sanz-Martín I, Sanz-Sánchez I, Noguerol F, Cok S, Ortiz-Vigón A, Sanz M. Randomized controlled clinical trial comparing two dental implants with different neck configurations. Clin Implant Dent Relat Res. 2017 Jun;19(3):512-522.
227. Sayardoust S, Gröndahl K, Johansson E, Thomsen P, Slotte C. Implant survival and marginal bone loss at turned and oxidized implants in periodontitis-susceptible smokers and never-smokers: a retrospective, clinical, radiographic case-control study. J Periodontol. 2013 Dec;84(12):1775-82.
228. Schlee M, Pradies G, Mehmke WU, Beneytout A, Stamm M, Meda RG, Kamm T, Poiroux F, Weinlich F, del Canto Pingarron M, Crichton E, Poulet JB, Bousquet P. Prospective, Multicenter Evaluation of Trabecular Metal-Enhanced Titanium Dental Implants Placed in Routine Dental

Practices: 1-Year Interim Report From the Development Period (2010 to 2011). Clin Implant Dent Relat Res. 2015 Dec;17(6):1141-53.
229. Schmid E, Morandini M, Roccuzzo A, Ramseier CA, Sculean A, Salvi GE. Clinical and radiographic outcomes of implant-supported fixed dental prostheses with cantilever extension. A retrospective cohort study with a follow-up of at least 10 years. Clin Oral Implants Res. 2020 Dec;31(12):1243-1252.
230. Schmid E, Roccuzzo A, Morandini M, Ramseier CA, Sculean A, Salvi GE. Clinical and radiographic evaluation of implant-supported single-unit crowns with cantilever extension in posterior areas: A retrospective study with a follow-up of at least 10 years. Clin Implant Dent Relat Res. 2021 Apr;23(2):189-196.
231. Schneider D, Witt L, Hämmerle CHF. Influence of the crown-to-implant length ratio on the clinical performance of implants supporting single crown restorations: a cross-sectional retrospective 5-year investigation. Clin Oral Implants Res. 2012 Feb;23(2):169-174.
232. Schoenbaum TR, Moy PK, Aghaloo T, Elashoff D. Risk Factors for Dental Implant Failure in Private Practice: A Multicenter Survival Analysis. Int J Oral Maxillofac Implants. 2021 Mar-Apr;36(2):388-394.
233. Schwartz-Arad D, Grossman Y, Chaushu G. The clinical effectiveness of implants placed immediately into fresh extraction sites of molar teeth. J Periodontol. 2000 May;71(5):839-44.
234. Schwartz-Arad D, Ofec R, Eliyahu G, Ruban A, Sterer N. Long Term Follow-Up of Dental Implants Placed in Autologous Onlay Bone Graft. Clin Implant Dent Relat Res. 2016 Jun;18(3):449-61.
235. Schwartz-Arad D, Samet N, Samet N, Mamlider A. Smoking and complications of endosseous dental implants. J Periodontol. 2002 Feb;73(2):153-7.
236. Shibuya Y, Takeuchi Y, Asai T, Takeuchi J, Suzuki H, Komori T. Maxillary sinus floor elevation combined with a vertical onlay graft. Implant Dent. 2012 Apr;21(2):91-6.
237. Si M, Zhang Y, Li J, He F. Retrospective study on the clinical outcomes of small-diameter implants supporting fixed prostheses without bone augmentation in the posterior region after 2 to 12 years. Clin Implant Dent Relat Res. 2019 Jun;21(3):454-461.
238. Si MS, Shou YW, Shi YT, Yang GL, Wang HM, He FM. Long-term outcomes of osteotome sinus floor elevation without bone grafts: a clinical retrospective study of 4-9 years. Clin Oral Implants Res. 2016 Nov;27(11):1392-1400.
239. Sicilia A, Gallego L, Sicilia P, Mallo C, Cuesta S, Sanz M. Crestal bone loss associated with different implant surfaces in the posterior mandible in patients with a history of periodontitis. A retrospective study. Clin Oral Implants Res. 2021 Jan;32(1):88-99.
240. Siebers D, Gehrke P, Schliephake H. Delayed function of dental implants: a 1- to 7-year follow-up study of 222 implants. Int J Oral Maxillofac Implants. 2010 Nov-Dec;25(6):1195-202.
241. Simons WF, De Smit M, Duyck J, Coucke W, Quirynen M. The proportion of cancellous bone as predictive factor for early marginal bone loss around implants in the posterior part of the mandible. Clin Oral Implants Res. 2015 Sep;26(9):1051-9.
242. Sivolella S, Botticelli D, Prasad S, Ricci S, Bressan E, Prasad H. Evaluation and comparison of histologic changes and implant survival in extraction sites immediately grafted with two different xenografts: A randomized clinical pilot study. Clin Oral Implants Res. 2020 Sep;31(9):825-835.
243. Souza CSV, Ortega-Lopes R, Barreno AC, de Moraes M, Albergaria-Barbosa JR, Nóia CF. Analysis of the Survival of Dental Implants Installed in Reconstructed Maxilla With Autogenous Iliac Crest Graft: 7- to 9-Year Follow-Up. J Oral Implantol. 2019 Dec;45(6):427-436.
244. Stacchi C, Troiano G, Rapani A, Lombardi T, Sentineri R, Speroni S, Berton F, Di Lenarda R. Factors influencing the prevalence of peri-implantitis in implants inserted in augmented maxillary sinuses: A multicenter cross-sectional study. J Periodontol. 2021 Aug;92(8):1117-1125.
245. Stoker G, van Waas R, Wismeijer D. Long-term outcomes of three types of implant-supported mandibular overdentures in smokers. Clin Oral Implants Res. 2012 Aug;23(8):925-9.

246. Strietzel FP, Karmon B, Lorean A, Fischer PP. Implant-prosthetic rehabilitation of the edentulous maxilla and mandible with immediately loaded implants: preliminary data from a retrospective study, considering time of implantation. Int J Oral Maxillofac Implants. 2011 Jan-Feb;26(1):139-47.
247. Sverzut AT, Stabile GA, de Moraes M, Mazzonetto R, Moreira RW. The influence of tobacco on early dental implant failure. J Oral Maxillofac Surg. 2008 May;66(5):1004-9.
248. Tallarico M, Canullo L, Pisano M, Peñarrocha-Oltra D, Peñarrocha-Diago M, Meloni SM. An up to 7-Year Retrospective Analysis of Biologic and Technical Complication With the All-on-4 Concept. J Oral Implantol. 2016 Jun;42(3):265-71.
249. Tallarico M, Esposito M, Xhanari E, Caneva M, Meloni SM. Computer-guided vs freehand placement of immediately loaded dental implants: 5-year postloading results of a randomised controlled trial. Eur J Oral Implantol. 2018;11(2):203-213.
250. Tallarico M, Meloni SM. Retrospective Analysis on Survival Rate, Template-Related Complications, and Prevalence of Peri-implantitis of 694 Anodized Implants Placed Using Computer-Guided Surgery: Results Between 1 and 10 Years of Follow-Up. Int J Oral Maxillofac Implants. 2017 Sep/Oct;32(5):1162-1171.
251. Tartaglia GM, Maiorana C, Gallo M, Codari M, Sforza C. Implant-Supported Immediately Loaded Full-Arch Rehabilitations: Comparison of Resin and Zirconia Clinical Outcomes in a 5-Year Retrospective Follow-Up Study. Implant Dent. 2016 Feb;25(1):74-82.
252. Taschieri S, Lolato A, Testori T, Francetti L, Del Fabbro M. Short dental implants as compared to maxillary sinus augmentation procedure for the rehabilitation of edentulous posterior maxilla: Three-year results of a randomized clinical study. Clin Implant Dent Relat Res. 2018 Feb;20(1):9-20.
253. Tattan M, Puranam M, Comnick C, McBrearty C, Xie XJ, Caplan DJ, Avila-Ortiz G, Elangovan S. Surgery start time and early implant failure: A case-control study. Clin Oral Implants Res. 2021 Jul;32(7):871-880.
254. Tawil G, Younan R, Azar P, Sleilati G. Conventional and advanced implant treatment in the type II diabetic patient: surgical protocol and long-term clinical results. Int J Oral Maxillofac Implants. 2008 Jul-Aug;23(4):744-52.
255. Tealdo T, Menini M, Bevilacqua M, Pera F, Capalbo V, Pera P. Brånemark Novum immediate loading rehabilitation of edentulous mandibles: 11-year retrospective study. Clin Oral Implants Res. 2015 Jan;26(1):83-9.
256. Temmerman A, Keestra JA, Coucke W, Teughels W, Quirynen M. The outcome of oral implants placed in bone with limited bucco-oral dimensions: a 3-year follow-up study. J Clin Periodontol. 2015 Mar;42(3):311-8.
257. Testori T, Galli F, Fumagalli L, Capelli M, Zuffetti F, Deflorian M, Parenti A, Del Fabbro M. Assessment of Long-Term Survival of Immediately Loaded Tilted Implants Supporting a Maxillary Full-Arch Fixed Prosthesis. Int J Oral Maxillofac Implants. 2017 Jul/Aug;32(4):904-911.
258. Testori T, Taschieri S, Scutellà F, Del Fabbro M. Immediate Versus Delayed Loading of Postextraction Implants: A Long-Term Retrospective Cohort Study. Implant Dent. 2017 Dec;26(6):853-859.
259. Testori T, Wiseman L, Woolfe S, Porter SS. A prospective multicenter clinical study of the Osseotite implant: four-year interim report. Int J Oral Maxillofac Implants. 2001 Mar-Apr;16(2):193-200.
260. Testori T, Zuffetti F, Capelli M, Galli F, Weinstein RL, Del Fabbro M. Immediate versus conventional loading of post-extraction implants in the edentulous jaws. Clin Implant Dent Relat Res. 2014 Dec;16(6):926-35.
261. Thoma DS, Gasser TJW, Jung RE, Hämmerle CHF. Randomized controlled clinical trial comparing implant sites augmented with a volume-stable collagen matrix or an autogenous connective tissue graft: 3-year data after insertion of reconstructions. J Clin Periodontol. 2020 May;47(5):630-639.

262. Thoma DS, Haas R, Sporniak-Tutak K, Garcia A, Taylor TD, Hämmerle CHF. Randomized controlled multicentre study comparing short dental implants (6 mm) versus longer dental implants (11-15 mm) in combination with sinus floor elevation procedures: 5-Year data. J Clin Periodontol. 2018 Dec;45(12):1465-1474.
263. Thoma DS, Maggetti I, Waller T, Hämmerle CHF, Jung RE. Clinical and patient-reported outcomes of implants placed in autogenous bone grafts and implants placed in native bone: A case-control study with a follow-up of 5-16 years. Clin Oral Implants Res. 2019 Mar;30(3):242-251.
264. Troiano G, Luongo R, Romano DC, Galli M, Ravidà A, Wang HL, Laino L. Comparison of immediate versus delayed implant placement in a failed implant site: A retrospective analysis of early implant survival. Int J Oral Implantol (Berl). 2021 Mar 16;14(1):67-76.
265. Twito D, Sade P. The effect of cigarette smoking habits on the outcome of dental implant treatment. PeerJ. 2014 Sep 2;2:e546.
266. Urdaneta RA, Daher S, Leary J, Emanuel KM, Chuang SK. The survival of ultrashort locking-taper implants. Int J Oral Maxillofac Implants. 2012 May-Jun;27(3):644-54.
267. Uribarri A, Bilbao E, Marichalar-Mendia X, Martínez-Conde R, Aguirre JM, Verdugo F. Bone Remodeling around Implants Placed in Augmented Sinuses in Patients with and without History of Periodontitis. Clin Implant Dent Relat Res. 2017 Apr;19(2):268-279.
268. van Steenberghe D, Molly L, Jacobs R, Vandekerckhove B, Quirynen M, Naert I. The immediate rehabilitation by means of a ready-made final fixed prosthesis in the edentulous mandible: a 1-year follow-up study on 50 consecutive patients. Clin Oral Implants Res. 2004 Jun;15(3):360-5.
269. Vandeweghe S, Ackermann A, Bronner J, Hattingh A, Tschakaloff A, De Bruyn H. A retrospective, multicenter study on a novo wide-body implant for posterior regions. Clin Implant Dent Relat Res. 2012 Apr;14(2):281-92.
270. Vandeweghe S, De Bruyn H. The effect of smoking on early bone remodeling on surface modified Southern Implants®. Clin Implant Dent Relat Res. 2011 Sep;13(3):206-14.
271. Vandeweghe S, Nicolopoulos C, Thevissen E, Jimbo R, Wennerberg A, De Bruyn H. Immediate loading of screw-retained all-ceramic crowns in immediate versus delayed single implant placement. Int J Prosthodont. 2013 Sep-Oct;26(5):458-64.
272. Vervaeke S, Collaert B, Cosyn J, Deschepper E, De Bruyn H. A multifactorial analysis to identify predictors of implant failure and peri-implant bone loss. Clin Implant Dent Relat Res. 2015 Jan;17 Suppl 1:e298-307.
273. Waechter J, Madruga MM, Carmo Filho LCD, Leite FRM, Schinestsck AR, Faot F. Comparison between tapered and cylindrical implants in the posterior regions of the mandible: A prospective, randomized, split-mouth clinical trial focusing on implant stability changes during early healing. Clin Implant Dent Relat Res. 2017 Aug;19(4):733-741.
274. Wagenberg B, Froum SJ. A retrospective study of 1925 consecutively placed immediate implants from 1988 to 2004. Int J Oral Maxillofac Implants. 2006 Jan-Feb;21(1):71-80.
275. Wagenberg BD, Froum SJ, Eckert SE. Long-term bone stability assessment around 1,187 immediately placed implants with 1- to 22-year follow-up. Int J Oral Maxillofac Implants. 2013 Mar-Apr;28(2):605-12.
276. Wallace RH. The relationship between cigarette smoking and dental implant failure. Eur J Prosthodont Restor Dent. 2000 Sep;8(3):103-6.
277. Wang F, Monje A, Huang W, Zhang Z, Wang G, Wu Y. Maxillary Four Implant-retained Overdentures via Locator® Attachment: Intermediate-term Results from a Retrospective Study. Clin Implant Dent Relat Res. 2016 Jun;18(3):571-9.
278. Wang J, Lerman G, Bittner N, Fan W, Lalla E, Papapanou PN. Immediate versus delayed temporization at posterior single implant sites: A randomized controlled trial. J Clin Periodontol. 2020 Oct;47(10):1281-1291.

279. Wennström JL, Ekestubbe A, Gröndahl K, Karlsson S, Lindhe J. Oral rehabilitation with implant-supported fixed partial dentures in periodontitis-susceptible subjects. A 5-year prospective study. J Clin Periodontol. 2004 Sep;31(9):713-24.
280. Werbelow L, Weiss M, Schramm A. Long-term follow-up of full-arch immediate implant-supported restorations in edentulous jaws: a clinical study. Int J Implant Dent. 2020 Jul 30;6(1):34.
281. Widmark G, Andersson B, Carlsson GE, Lindvall AM, Ivanoff CJ. Rehabilitation of patients with severely resorbed maxillae by means of implants with or without bone grafts: a 3- to 5-year follow-up clinical report. Int J Oral Maxillofac Implants. 2001 Jan-Feb;16(1):73-9.
282. Windael S, Collaert B, De Buyser S, De Bruyn H, Vervaeke S. Early peri-implant bone loss as a predictor for peri-implantitis: A 10-year prospective cohort study. Clin Implant Dent Relat Res. 2021 Jun;23(3):298-308.
283. Windael S, Vervaeke S, Wijnen L, Jacquet W, De Bruyn H, Collaert B. Ten-year follow-up of dental implants used for immediate loading in the edentulous mandible: A prospective clinical study. Clin Implant Dent Relat Res. 2018 Aug;20(4):515-521.
284. Wolf F, Spoerl S, Gottsauner M, Klingelhöffer C, Spanier G, Kolbeck C, Reichert TE, Hautmann MG, Ettl T. Significance of site-specific radiation dose and technique for success of implant-based prosthetic rehabilitation in irradiated head and neck cancer patients-A cohort study. Clin Implant Dent Relat Res. 2021 Jun;23(3):444-455.
285. Wu S, Wu X, Shrestha R, Lin J, Feng Z, Liu Y, Shi Y, Huang B, Li Z, Liu Q, Zhang X, Hu M, Chen Z. Clinical and Radiologic Outcomes of Submerged and Nonsubmerged Bone-Level Implants with Internal Hexagonal Connections in Immediate Implantation: A 5-Year Retrospective Study. J Prosthodont. 2018 Feb;27(2):101-107.
286. Wu X, Al-Abedalla K, Abi-Nader S, Daniel NG, Nicolau B, Tamimi F. Proton Pump Inhibitors and the Risk of Osseointegrated Dental Implant Failure: A Cohort Study. Clin Implant Dent Relat Res. 2017 Apr;19(2):222-232.
287. Wu X, Al-Abedalla K, Eimar H, Arekunnath Madathil S, Abi-Nader S, Daniel NG, Nicolau B, Tamimi F. Antihypertensive Medications and the Survival Rate of Osseointegrated Dental Implants: A Cohort Study. Clin Implant Dent Relat Res. 2016 Dec;18(6):1171-1182.
288. Wu X, Al-Abedalla K, Rastikerdar E, Abi Nader S, Daniel NG, Nicolau B, Tamimi F. Selective serotonin reuptake inhibitors and the risk of osseointegrated implant failure: a cohort study. J Dent Res. 2014 Nov;93(11):1054-61.
289. Zafiropoulos GG, Deli G, Bartee BK, Hoffmann O. Single-tooth implant placement and loading in fresh and regenerated extraction sockets. Five-year results: a case series using two different implant designs. J Periodontol. 2010 Apr;81(4):604-15.
290. Zembic A, Tahmaseb A, Jung RE, Wismeijer D. One-year results of maxillary overdentures supported by 2 titanium-zirconium implants - implant survival rates and radiographic outcomes. Clin Oral Implants Res. 2017 Jul;28(7):e60-e67.
291. Zinser MJ, Randelzhofer P, Kuiper L, Zöller JE, De Lange GL. The predictors of implant failure after maxillary sinus floor augmentation and reconstruction: a retrospective study of 1045 consecutive implants. Oral Surg Oral Med Oral Pathol Oral Radiol. 2013 May;115(5):571-82.
292. Zumstein T, Schütz S, Sahlin H, Sennerby L. Factors influencing marginal bone loss at a hydrophilic implant design placed with or without GBR procedures: A 5-year retrospective study. Clin Implant Dent Relat Res. 2019 Oct;21(5):817-826.

c. Figure S1. Forest plot for the event 'implant failure', global results (estimate in odds ratio).

Studies	Estimate (95% C.I.)
Abduljabbar 2018	0.897 (0.017, 46.175)
Abi-Aad 2018	7.721 (0.409, 145.655)
Agliardi (1) 2010	4.879 (0.093, 254.792)
Agliardi (2) 2014	1.923 (0.118, 31.246)
Al Amri 2017	0.851 (0.016, 44.255)
Al Najam 2021	0.519 (0.064, 4.209)
Al-Aali 2018	1.058 (0.021, 53.900)
Al-Sabbagh 2015	1.025 (0.436, 2.407)
Alahmari 2019	0.521 (0.010, 26.745)
Alasqah 2019	1.101 (0.021, 56.826)
Alghamdi 2020	0.962 (0.018, 50.349)
Aloy-Prosper 2016	8.818 (0.159, 490.150)
Alqahtani 2020	0.981 (0.019, 50.375)
Alsaadi (1) 2007	1.845 (1.354, 2.514)
Alsaadi (2) 2008	4.889 (1.519, 15.732)
Alsaasi (3) 2008	1.574 (0.952, 2.603)
Angelis 2020	2.714 (0.051, 145.737)
Anitua (1) 2008	2.606 (1.230, 5.523)
Anitua (2) 2019	4.733 (0.091, 244.899)
Anner 2010	2.459 (1.458, 4.146)
Arora (1) 2018	19.667 (0.281, 1377.852)
Arora (2) 2018	8.111 (0.143, 461.104)
Atarchi 2020	7.982 (4.370, 14.578)
Aykent 2007	17.870 (0.935, 341.502)
Bain (1) 1993	2.540 (1.735, 3.719)
Bain (2) 1996	3.932 (1.495, 10.341)
Balaguer 2015	2.535 (0.854, 7.523)
Balshi 2014	3.014 (1.238, 7.336)
Barbato 2018	6.122 (2.029, 18.477)
Barnea 2016	1.878 (0.036, 98.162)
Barone (1) 2014	1.480 (0.028, 79.608)
Barone (2) 2016	1.162 (0.102, 13.248)
Bell (1) 2011	1.640 (0.456, 5.895)
Bell (2) 2014	5.800 (0.544, 61.866)
Bischof 2006	0.990 (0.108, 9.049)
Boardman 2015	12.200 (0.226, 659.462)
Boboeva 2021	1.508 (0.593, 3.837)
Bouhy 2020	2.556 (0.709, 9.206)
Brandt 2012	39.578 (2.144, 730.601)
Cakarer 2014	1.903 (0.670, 5.402)
Cannizzaro (1) 2003	26.053 (0.990, 685.681)
Cannizzaro (2) 2008	0.193 (0.009, 4.114)
Cannizzaro (3) 2012	1.522 (0.091, 25.563)
Cannizzaro (4) 2016	0.633 (0.025, 15.951)
Cannizzaro (5) 2016	2.481 (0.048, 127.195)
Cannizzaro (6) 2017	2.732 (0.053, 139.592)
Cannizzaro (7) 2018	0.673 (0.127, 3.579)
Cannizzaro (8) 2018	1.522 (0.091, 25.563)
Canullo 2014	9.000 (0.330, 245.378)
Cappare 2019	37.364 (2.130, 655.285)
Carr 2019	1.359 (1.031, 1.792)
Cavalcanti (1) 2011	1.929 (1.473, 2.527)
Cha 2014	7.683 (2.719, 21.707)
Chang 2020	3.088 (1.038, 9.187)
Chiapasco (1) 2018	0.803 (0.041, 15.785)
Chiapasco (2) 2020	6.500 (0.393, 107.418)
Chrcanovic (1) 2017	2.037 (1.503, 2.760)
Chrcanovic (2) 2017	1.626 (1.319, 2.004)
Chrcanovic (3) 2018	1.548 (1.012, 2.367)
Clauser 2020	1.770 (0.771, 4.068)
Conrad 2011	1.723 (0.681, 4.355)
Corvino 2020	3.759 (0.071, 197.657)
Coskunses 2021	0.848 (0.034, 21.167)
Crespi 2019	5.571 (0.094, 330.090)
Cristalli 2015	3.600 (0.190, 68.341)
Cucchi 2019	3.689 (0.780, 17.451)
D'haese 2013	32.889 (4.080, 265.120)
Daher 2019	12.337 (0.692, 219.835)
Daneshvar 2016	0.151 (0.008, 2.721)
Daubert 2015	0.368 (0.021, 6.423)
De Bruyn (1) 1994	5.463 (1.569, 19.021)
De Bruyn (2) 1999	0.639 (0.196, 2.081)
Degidi 2015	16.852 (2.041, 139.172)
Deli 2014	1.346 (0.026, 68.460)
Di Stefano 2020	2.818 (0.127, 62.528)
Doan 2014	32.278 (13.860, 75.171)
Donati (1) 2015	0.398 (0.021, 7.569)
Donati (2) 2018	0.667 (0.206, 2.154)
Donos 2019	3.857 (0.064, 230.985)
Doyle 2007	8.429 (1.863, 38.125)
Engstrand 2003	1.619 (0.584, 4.489)
Feher 2020	1.778 (0.615, 5.141)
Felice 2015	8.243 (0.375, 181.309)
Flores-Guillen 2018	2.280 (0.043, 121.522)
Fonseca 2021	4.467 (0.082, 243.649)
Francetti (1) 2014	5.757 (0.223, 148.406)
Francetti (2) 2019	1.227 (0.233, 6.458)
Friberg 2008	3.038 (1.364, 6.766)
Gamper 2017	4.444 (0.599, 32.970)
Gander 2014	9.730 (1.248, 75.881)
Gaspersic 2021	30.600 (0.967, 967.916)
Geurs 2001	2.838 (1.077, 7.481)
Ghazal 2019	19.400 (0.315, 1195.297)
Gherlone 2016	0.422 (0.129, 1.377)
Gjelvold 2020	21.000 (0.933, 472.586)
Gorman 1994	2.031 (1.325, 3.113)
Grandi (1) 2012	3.247 (0.064, 166.018)

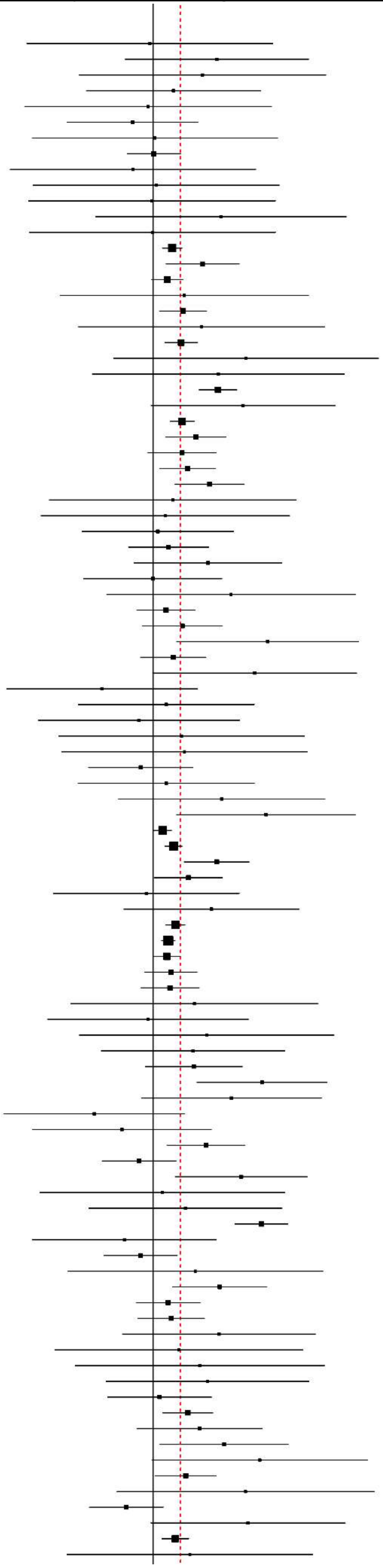

Grandi (2) 2012	2.081	(0.040, 109.053)
Grandi (3) 2012	3.595	(0.069, 187.367)
Grandi (4) 2013	3.067	(0.060, 156.857)
Grandi (5) 2014	1.737	(0.032, 94.864)
Grandi (6) 2015	3.292	(0.198, 54.631)
Grisar 2017	3.176	(1.807, 5.584)
Groenendijk 2020	6.920	(0.131, 364.656)
Grossmann 2008	10.926	(0.490, 243.484)
Grunder 1999	0.416	(0.021, 8.175)
Guarnieri (1) 2019	11.609	(0.443, 304.105)
Guarnieri (2) 2020	7.259	(2.063, 25.538)
Guida 2020	3.254	(0.063, 166.946)
Göthberg 2018	0.455	(0.052, 4.005)
Habsha 2000	1.147	(0.754, 1.746)
Hakam 2021	3.480	(1.594, 7.597)
Han 2018	1.747	(0.083, 36.759)
Hartlev 2021	1.286	(0.050, 32.779)
Hattingh (1) 2018	7.154	(0.130, 392.516)
Hattingh (2) 2019	3.732	(0.072, 193.831)
He 2015	2.985	(1.591, 5.602)
Higuchi 2020	3.954	(0.963, 16.233)
Hingsammer 2018	6.429	(0.109, 379.649)
Hinze 2010	0.943	(0.176, 5.054)
Hof 2014	8.935	(0.171, 466.357)
Holahan 2008	2.638	(1.227, 5.674)
Horwitz 2012	27.783	(1.388, 556.062)
Hsu 2016	6.714	(0.114, 396.170)
Hu 2020	1.479	(0.078, 27.911)
Ibañez (1) 2003	2.021	(0.335, 12.201)
Ibañez (2) 2005	0.894	(0.042, 18.861)
Iorio−Siciliano 2016	2.231	(0.040, 125.207)
Ji 2012	1.037	(0.401, 2.681)
Jones 1999	4.056	(1.376, 11.957)
Kan 1999	2.765	(1.155, 6.618)
Karoussis 2003	2.077	(0.329, 13.116)
Keller 1999	2.046	(0.805, 5.201)
Khouly 2017	3.867	(1.415, 10.563)
Kim 2015	5.340	(0.103, 275.865)
Kinsel 2007	2.121	(0.767, 5.868)
Klotz 2019	1.659	(0.397, 6.925)
Kolerman 2017	15.000	(0.548, 410.590)
Komiyama 2008	8.254	(2.976, 22.894)
Krennmair (1) 2016	7.049	(0.136, 365.031)
Krennmair (2) 2019	4.528	(0.279, 73.558)
Kumar 2002	1.837	(0.770, 4.381)
Lambert 2000	1.533	(1.145, 2.053)
Le 2013	1.361	(0.163, 11.358)
Lee (1) 2018	0.888	(0.114, 6.947)
Lee (2) 2020	13.800	(0.223, 853.583)
Leventi 2014	10.021	(0.395, 254.253)
Levin (1) 2008	3.067	(0.266, 35.329)
Levin (2) 2011	1.374	(0.829, 2.278)
Lin 2012	1.813	(0.767, 4.287)
Lindquist 1997	0.155	(0.008, 3.038)
Lini 2019	9.910	(4.355, 22.551)
Lobato 2020	1.889	(0.070, 50.876)
Locante 2004	2.684	(0.102, 70.685)
Lombardo 2020	1.074	(0.117, 9.895)
Luongo 2020	3.258	(1.103, 9.625)
Malchiodi (1) 2014	23.957	(1.354, 423.820)
Malchiodi (2) 2016	2.905	(0.054, 155.791)
Maló (1) 2013	0.667	(0.073, 6.061)
Maló (2) 2019	2.008	(1.385, 2.909)
Maló (3) 2019	2.827	(1.747, 4.576)
Mastrangelo 2018	16.509	(0.768, 354.688)
Mayfield 2001	98.077	(4.985, 1929.655)
McCarthy 2003	0.244	(0.051, 1.167)
Mendonca 2014	1.303	(0.370, 4.583)
Merli (1) 2017	3.737	(0.073, 192.463)
Merli (2) 2020	2.088	(0.079, 55.098)
Mertens 2012	11.667	(0.585, 232.719)
Migliorati 2015	18.600	(0.302, 1146.480)
Mijiritsky (1) 2013	1.200	(0.605, 2.380)
Mijiritsky (2) 2015	4.059	(0.075, 219.647)
Mijiritsky (3) 2016	14.200	(0.230, 877.991)
Minsk 1996	1.210	(0.678, 2.157)
Mir−Mari 2012	2.380	(0.047, 121.236)
Moheng 2005	9.091	(1.790, 46.177)
Moraguez 2016	3.667	(0.063, 211.754)
Morales−Vadillo 2013	1.811	(1.058, 3.100)
Mordenfeld 2014	4.772	(1.409, 16.159)
Mundt 2006	7.758	(2.954, 20.375)
Munoz 2021	61.000	(3.218, 1156.334)
Naeini 2018	4.846	(0.088, 267.206)
Nedir 2004	8.096	(0.727, 90.145)
Niedermaier 2017	0.213	(0.083, 0.544)
Nitzan 2005	0.457	(0.092, 2.283)
Noelken 2020	17.000	(0.631, 457.882)
Nogueira 2018	0.737	(0.035, 15.667)
Noguerol 2006	2.798	(1.699, 4.606)
Norton 2017	19.667	(0.281, 1377.852)
Nyström 2009	0.951	(0.329, 2.751)
Oliva 2012	1.179	(0.023, 61.046)
Olmedo−Gaya 2016	3.423	(1.228, 9.536)
Olson 2000	2.612	(0.230, 29.646)
Omran 2015	10.857	(2.618, 45.026)
Park 2019	1.780	(0.953, 3.324)
Patil 2020	4.467	(0.142, 140.552)
Peleg 2006	1.381	(0.742, 2.571)
Penarrocha 2014	3.750	(0.222, 63.300)

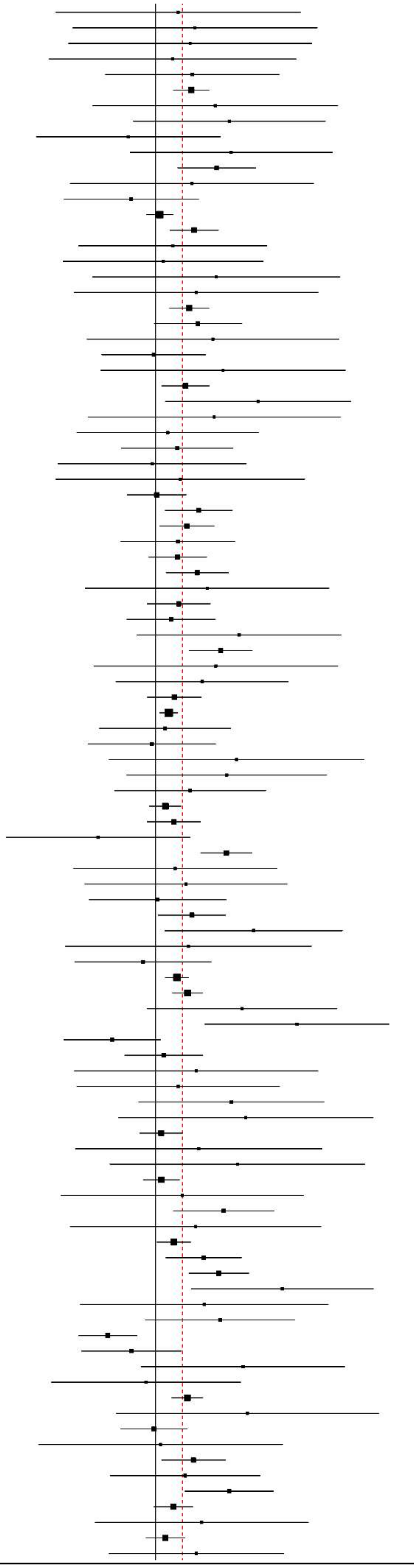

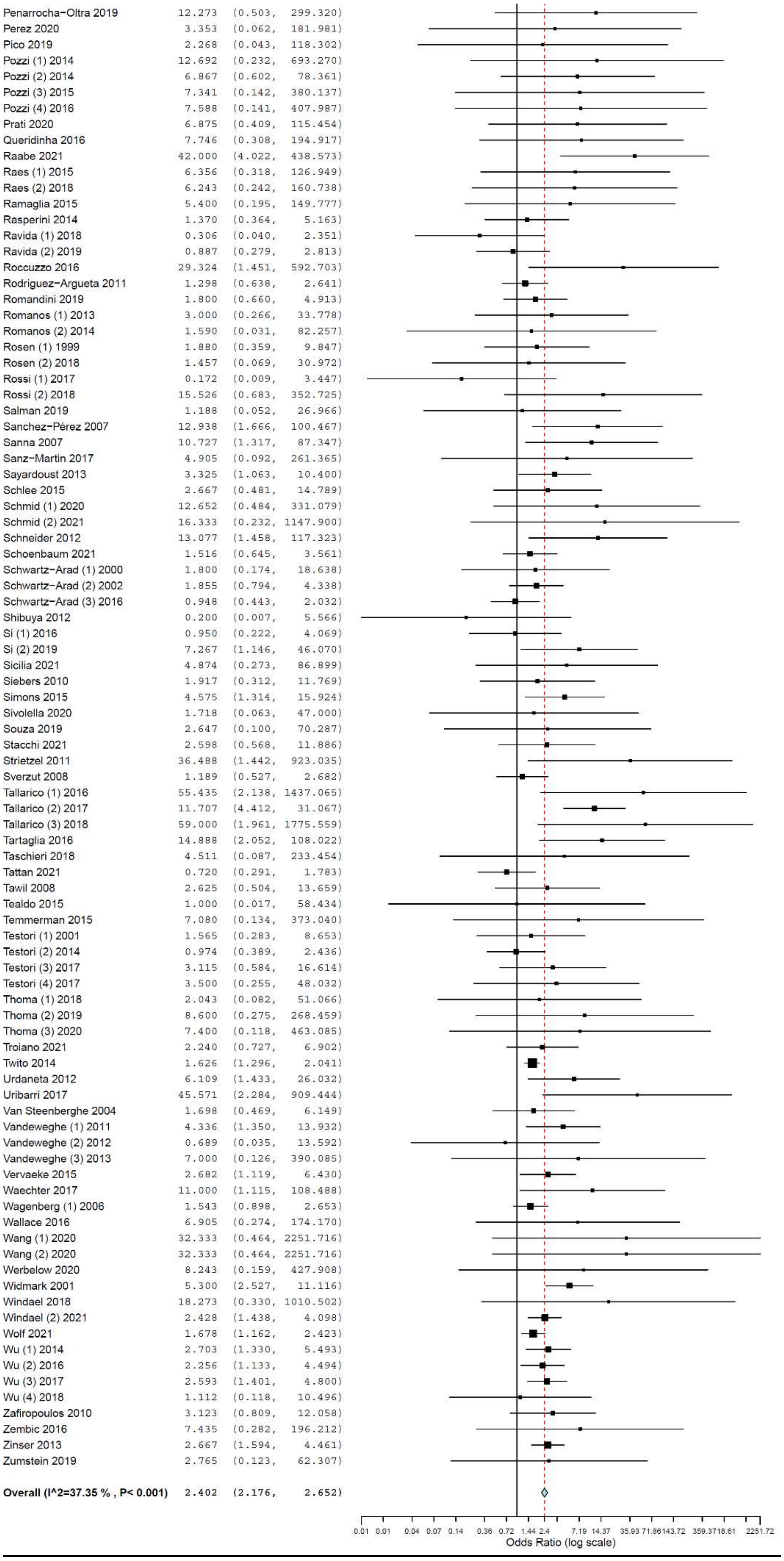
Penarrocha−Oltra 2019 12.273 (0.503, 299.320)
Perez 2020 3.353 (0.062, 181.981)
Pico 2019 2.268 (0.043, 118.302)
Pozzi (1) 2014 12.692 (0.232, 693.270)
Pozzi (2) 2014 6.867 (0.602, 78.361)
Pozzi (3) 2015 7.341 (0.142, 380.137)
Pozzi (4) 2016 7.588 (0.141, 407.987)
Prati 2020 6.875 (0.409, 115.454)
Queridinha 2016 7.746 (0.308, 194.917)
Raabe 2021 42.000 (4.022, 438.573)
Raes (1) 2015 6.356 (0.318, 126.949)
Raes (2) 2018 6.243 (0.242, 160.738)
Ramaglia 2015 5.400 (0.195, 149.777)
Rasperini 2014 1.370 (0.364, 5.163)
Ravida (1) 2018 0.306 (0.040, 2.351)
Ravida (2) 2019 0.887 (0.279, 2.813)
Roccuzzo 2016 29.324 (1.451, 592.703)
Rodriguez−Argueta 2011 1.298 (0.638, 2.641)
Romandini 2019 1.800 (0.660, 4.913)
Romanos (1) 2013 3.000 (0.266, 33.778)
Romanos (2) 2014 1.590 (0.031, 82.257)
Rosen (1) 1999 1.880 (0.359, 9.847)
Rosen (2) 2018 1.457 (0.069, 30.972)
Rossi (1) 2017 0.172 (0.009, 3.447)
Rossi (2) 2018 15.526 (0.683, 352.725)
Salman 2019 1.188 (0.052, 26.966)
Sanchez−Pérez 2007 12.938 (1.666, 100.467)
Sanna 2007 10.727 (1.317, 87.347)
Sanz−Martin 2017 4.905 (0.092, 261.365)
Sayardoust 2013 3.325 (1.063, 10.400)
Schlee 2015 2.667 (0.481, 14.789)
Schmid (1) 2020 12.652 (0.484, 331.079)
Schmid (2) 2021 16.333 (0.232, 1147.900)
Schneider 2012 13.077 (1.458, 117.323)
Schoenbaum 2021 1.516 (0.645, 3.561)
Schwartz−Arad (1) 2000 1.800 (0.174, 18.638)
Schwartz−Arad (2) 2002 1.855 (0.794, 4.338)
Schwartz−Arad (3) 2016 0.948 (0.443, 2.032)
Shibuya 2012 0.200 (0.007, 5.566)
Si (1) 2016 0.950 (0.222, 4.069)
Si (2) 2019 7.267 (1.146, 46.070)
Sicilia 2021 4.874 (0.273, 86.899)
Siebers 2010 1.917 (0.312, 11.769)
Simons 2015 4.575 (1.314, 15.924)
Sivolella 2020 1.718 (0.063, 47.000)
Souza 2019 2.647 (0.100, 70.287)
Stacchi 2021 2.598 (0.568, 11.886)
Strietzel 2011 36.488 (1.442, 923.035)
Sverzut 2008 1.189 (0.527, 2.682)
Tallarico (1) 2016 55.435 (2.138, 1437.065)
Tallarico (2) 2017 11.707 (4.412, 31.067)
Tallarico (3) 2018 59.000 (1.961, 1775.559)
Tartaglia 2016 14.888 (2.052, 108.022)
Taschieri 2018 4.511 (0.087, 233.454)
Tattan 2021 0.720 (0.291, 1.783)
Tawil 2008 2.625 (0.504, 13.659)
Tealdo 2015 1.000 (0.017, 58.434)
Temmerman 2015 7.080 (0.134, 373.040)
Testori (1) 2001 1.565 (0.283, 8.653)
Testori (2) 2014 0.974 (0.389, 2.436)
Testori (3) 2017 3.115 (0.584, 16.614)
Testori (4) 2017 3.500 (0.255, 48.032)
Thoma (1) 2018 2.043 (0.082, 51.066)
Thoma (2) 2019 8.600 (0.275, 268.459)
Thoma (3) 2020 7.400 (0.118, 463.085)
Troiano 2021 2.240 (0.727, 6.902)
Twito 2014 1.626 (1.296, 2.041)
Urdaneta 2012 6.109 (1.433, 26.032)
Uribarri 2017 45.571 (2.284, 909.444)
Van Steenberghe 2004 1.698 (0.469, 6.149)
Vandeweghe (1) 2011 4.336 (1.350, 13.932)
Vandeweghe (2) 2012 0.689 (0.035, 13.592)
Vandeweghe (3) 2013 7.000 (0.126, 390.085)
Vervaeke 2015 2.682 (1.119, 6.430)
Waechter 2017 11.000 (1.115, 108.488)
Wagenberg (1) 2006 1.543 (0.898, 2.653)
Wallace 2016 6.905 (0.274, 174.170)
Wang (1) 2020 32.333 (0.464, 2251.716)
Wang (2) 2020 32.333 (0.464, 2251.716)
Werbelow 2020 8.243 (0.159, 427.908)
Widmark 2001 5.300 (2.527, 11.116)
Windael 2018 18.273 (0.330, 1010.502)
Windael (2) 2021 2.428 (1.438, 4.098)
Wolf 2021 1.678 (1.162, 2.423)
Wu (1) 2014 2.703 (1.330, 5.493)
Wu (2) 2016 2.256 (1.133, 4.494)
Wu (3) 2017 2.593 (1.401, 4.800)
Wu (4) 2018 1.112 (0.118, 10.496)
Zafiropoulos 2010 3.123 (0.809, 12.058)
Zembic 2016 7.435 (0.282, 196.212)
Zinser 2013 2.667 (1.594, 4.461)
Zumstein 2019 2.765 (0.123, 62.307)
Overall (I^2=37.35 % , P< 0.001) 2.402 (2.176, 2.652)
0.01 0.01 0.04 0.07 0.14 0.36 0.72 1.44 2.4 7.19 14.37 35.93 71.86143.72 359.3718.61 2251.72
Odds Ratio (log scale)

d. Table S1. Detailed data of the included studies.

Study	Year	Study design	Country / Setting	Patients (male/ female) (n)	Patients' Age Range (mean) (years)	Prosthetic loading	Implant location (maxilla and/or mandible)	Implant used	Type of smokers included [a]
**Abduljabbar**	2018	CCT (unicenter)	Saudi Arabia / University	56 (56/0)	NM (41)	Delayed (3-4 mo)	Mx, Md	Osseospeed (Astra Tech, Mölndal, Sweden)	29 smokers
**Abi-Aad**	2018	RCT (unicenter)	Lebanon / University	26 (12/14)	34-67 (49.5)	Immediate, delayed (3 mo)	Mx	NobelActive (Nobel Biocare, Göteborg, Sweden)	5 heavy smokers<br>9 extra-heavy smokers
**Agliardi (1)**	2010	CCT (unicenter)	Italy / Private practice	24 (13/11)	40-73 (60)	Immediate	Md	MkIV TiUnite, NobelSpeedy Groovy (Nobel Biocare, Göteborg, Sweden)	4 smokers
**Agliardi (2)**	2014	CCT (unicenter)	Italy / Private practice	32 (15/17)	44-68 (58)	Immediate	Mx	MK IV and NobelSpeedy Groovy (Nobel Biocare, Göteborg, Sweden)	11 light smokers
**Al Amri**	2017	RA (unicenter)	Saudi Arabia / University	61 (51/10)	33-53 (43)	Immediate, delayed (mean 3.2 mo)	Md	Straumann Bone Level (Straumann, Basel, Switzerland)	33 light and heavy smokers
**Al Najam**	2021	RA (unicenter)	Netherlands / Private Practice	110 (62/48)	18-40 (24)	Delayed (4 mo)	Mx, Md	Bone level (Straumann, Basel, Switzerland)	19 smokers
**Al-Aali**	2018	RA (unicenter)	Saudi Arabia / University	116 (116/0)	35-51 (44)	Delayed (mean 3.2 mo)	Mx, Md	NM	59 smokers, but the degree of smoking among the patients was not reported
**Alahmari**	2019	RA (unicenter)	Saudi Arabia / University	123 (123/0)	NM (43)	Delayed	Mx, Md	NM	41 cigarette smokers (minimum of 5 cig./day), 40 water pipe smokers
**Alasqah**	2019	RA (unicenter)	Saudi Arabia / University	82 (65/17)	NM (43)	NM	Md	NM (Straumann, Basel, Switzerland)	39 smokers (minimum of 1 cig./day)
**Alghamdi**	2020	RA (unicenter)	Saudi Arabia / University	51 (51/0)	NM (46)	Immediate	Mx, Md	NM	26 smokers, but the degree of smoking among the patients was not reported
**Aloy-Prosper**	2016	RA (unicenter)	Spain / University	53 (14/39)	18-67 (35-48)	Delayed (3 mo)	Mx, Md	TSA Avantblast (Phibo Dental Solutions)	2 light smokers<br>3 heavy smokers
**Alqahtani**	2020	RA (unicenter)	Saudi Arabia / University	101 (101/0)	NM (51-55)	NM	Mx, Md	NM	51 smokers

**Alsaadi (1)**	2007	RA (unicenter)	Belgium / University	2004 (792/1212)	NM	NM	Mx, Md	Brånemark, TiUnite (Nobel Biocare, Göteborg, Sweden)	Light, heavy, and extra-heavy smokers
**Alsaadi (2)**	2008	PS (unicenter)	Belgium / University	283 (96/187)	18-86 (56.2)	NM	Mx, Md	TiUnite (Nobel Biocare, Göteborg, Sweden)	NM
**Alsaadi (3)**	2008	RA (unicenter)	Belgium / University	412 (172/240)	NM	NM	Mx, Md	Brånemark, TiUnite (Nobel Biocare, Göteborg, Sweden)	61 smokers
**Al-Sabbagh**	2015	RA (unicenter)	USA / University	415 (174/241)	NM (59.4)	NM	Mx, Md	NM	46 smokers
**Angelis**	2020	RA (unicenter)	Italy / Private practice	38 (17/21)	51-76 (65)	Delayed (3 mo)	Mx, Md	Bone Level Tapered RC, SLA, Loxim, Roxolid (Straumann, Basel, Switzerland)	10 light smokers
**Anitua (1)**	2008	RA (multicenter)	Spain / NM	1060 (386/674)	17-91 (54)	Immediate, delayed (>3 mo)	Mx, Md	BTI (Biotechnology Institute, Vitoria, Spain)	221 light, heavy, and extra-heavy smokers
**Anitua (2)**	2019	RA (unicenter)	Spain / Private practice	113 (50/63)	32-77 (56)	Delayed (mean 4 mo)	Mx, Md	UnicCa (BTI Biotechnology Institute, Vitoria-Gasteiz, Spain)	NM
**Anner**	2010	RA (unicenter)	Israel / Private practice	475 (176/299)	NM (52)	NM	Mx, Md	NM	63 smokers
**Arora (1)**	2018	PS (unicenter)	Australia / Private practice	30 (13/17)	26-77 (52)	Delayed (3-4 mo)	Mx	NM (Straumann, Basel, Switzerland)	Only light smokers (<10 cig/day)
**Arora (2)**	2018	RA (unicenter)	Australia / Private practice	40 (16/24)	19-74 (50.6)	Immediate, delayed (3-4 mo)	Mx	Osseospeed (Astra Tech, Mölndal, Sweden)	3 light smokers<br>1 heavy smoker
**Atarchi**	2020	RA (multicenter)	USA / University	1343 (516/827)	18-96 (61.7)	NM	Mx	Several (Astra EV OsseoSpeed, NobelReplace, Biomet 3i, Straumann, Zimmer)	58 smokers
**Aykent**	2007	RA (unicenter)	Turkey / University	34 (14/20)	19-70 (44.5)	Delayed (3-6 mo)	Mx, Md	Calcitek (Sulzer Medical, Carlsbad, USA), SLA (Straumann, Basel, Switzerland)	NM
**Bain (1)**	1993	RA (unicenter)	Scotland / Private practice	540 (229/311)	12-86 (55)	NM	Mx, Md	Brånemark (Nobel Biocare, Göteborg, Sweden)	NM
**Bain (2)**	1996	RA (unicenter)	Scotland / Private practice	78 (NM)	NM	NM	Mx, Md	Brånemark (Nobel Biocare, Göteborg, Sweden)	NM
**Balaguer**	2015	PS (unicenter)	Spain / University	95 (43/52)	23-80 (55.9)	Delayed (3 mo)	Mx, Md	Tissue Level (Straumann, Basel, Switzerland)	18 smokers
**Balshi**	2014	RA (unicenter)	USA / Private practice	152 (NM)	NM	Immediate	Mx, Md	Brånemark (Nobel Biocare, Göteborg, Sweden)	13 light, heavy, and extra-heavy smokers
**Barbato**	2018	RA (unicenter)	Italy / Private practice	32 (10/22)	37-81 (55.4)	Delayed (6-9 mo)	Mx	TSA Advance (Defcon, Barcelona, Spain)	13 heavy and extra-heavy smokers

**Barnea**	2016	RA (unicenter)	Israel / Private practice	29 (16/13)	40-83 (64.5)	Delayed (6 mo)	Mx	Biocom (MIS Implant technologies, Bar Lev Industrial Park, Israel)	10 smokers
**Barone (1)**	2014	PS (unicenter)	Italy / Hospital	30 (16/14)	29-67 (43-51)	Immediate, delayed (4 mo)	Mx, Md	Blossom (Intra-Lock International, Boca Raton, USA)	12 light smokers
**Barone (2)**	2016	RCT (unicenter)	Italy / University	116 (39/77)	NM (51.4)	Delayed (3 mo)	Mx, Md	CT, Blossom (Intra-Lock International, Boca Raton, USA)	35 light smokers
**Bell (1)**	2011	RA (unicenter)	USA / Private practice	655 (NM)	NM	3 months	Mx, Md	SLA (Straumann, Waldenburg, Switzerland)	NM
**Bell (2)**	2014	RA (unicenter)	USA / Private practice	39 (NM)	24-88 (58)	Immediate, delayed	Mx	Oxidized (NobelActive, Nobel Biocare, Göteborg, Sweden)	NM
**Bischof**	2006	RA (unicenter)	Switzerland / Private practice	212 (91/121)	22-88 (49.9)	Delayed (mean 3.7 mo)	Mx, Md	SLA, ITI (Straumann, Waldenburg, Switzerland)	Light and heavy smokers
**Boardman**	2015	RA (unicenter)	Australia / Private practice	98 (21/77)	23-81 (51.2)	NM	Mx	Several (AstraTech, Straumann, Biomet 3i, Nobel Biocare)	7 smokers
**Boboeva**	2021	RA (unicenter)	South Korea / University	1295 (584/711)	15-86 (46.7, 71.4)	Delayed	Mx, Md	Several (Astra Tech, AB, Mölndal, Sweden; Dentis, Daegu, South Korea; Dentium, Seoul, South Korea; Straumann, Basel, Switzerland; Megagen Implant, Gyeongsan, South Korea; Osstem Implant, Seoul, South Korea).	78 smokers
**Bouhy**	2020	PS (unicenter)	Belgium / University	30 (17/13)	48-82 (66.4)	Delayed (3 mo)	Mx	Roxolid (Straumann, Basel, Switzerland)	4 light smokers (<10 cig/day)
**Brandt**	2012	PS (unicenter)	USA / University	34 (NM)	35-70 (NM)	Immediate	Md	MDL (Intra-Lock System International, Boca Raton, Florida)	NM
**Cakarer**	2014	RA (unicenter)	Turkey / University	274 (109/165)	19-84 (50.2)	Delayed (2-3 mo)	Mx, Md	Several (Astra Tech, Straumann, SwissPlus)	Yes, but the exact number was not informed
**Cannizzaro (1)**	2003	CCT (unicenter)	Italy / Private practice	28 (14/14)	18-72 (38)	Immediate, delayed	Mx, Md	Spline Twist MTX (Centerpulse Dental, Carlsbad, USA)	6 smokers
**Cannizzaro (2)**	2008	RCT (unicenter)	Italy / Private practice	60 (25/35)	36-80 (61)	Immediate, early (6 wk)	Md	Tapered SwissPlus (Zimmer Dental, Carlsbad, USA)	9 light smokers<br>21 heavy smokers
**Cannizzaro (3)**	2012	RCT (unicenter)	Italy / Private practice	30 (15/15)	18-57 (35)	Immediate, delayed (6 wk)	Mx, Md	NanoTite (Biomet 3i, Palm Beach, USA)	12 light and heavy smokers
**Cannizzaro (4)**	2016	RCT (unicenter)	Italy / Private practice	50 (25/25)	19-62 (40)	Immediate	Mx, Md	Syra and Syra SL (Sweden & Martina, Due Carrare, Italy)	11 light smokers<br>6 heavy smokers
**Cannizzaro (5)**	2016	RCT (unicenter)	Italy / Private practice	40 (21/19)	33-78 (56)	Immediate	Mx, Md	Prama PF tapered (Sweden & Martina, Due Carrare, Italy)	Light and heavy smokers

**Cannizzaro (6)**	2017	RCT (multicenter)	Italy / Private practice	60 (29/31)	37-83 (59)	Immediate	Md	NT Full Osseotite (Zimmer Biomet, Palm Beach Gardens, USA), ExFeel (MegaGen Implant, Gyeongbuk, South Korea)	11 light smokers<br>5 heavy smokers
**Cannizzaro (7)**	2018	RCT (multicenter)	Italy / Private practice	48 (28/20)	36-78 (58)	Immediate	Mx, Md	Syra and Syra SL (Sweden & Martina, Due Carrare, Italy)	13 light smokers<br>4 heavy smokers
**Cannizzaro (8)**	2018	RCT (unicenter)	Italy / Private practice	30 (15/15)	18-57 (35)	Immediate, delayed (6 wk)	Mx, Md	NanoTite (Biomet 3i, Palm Beach Gardens, USA)	9 light smokers<br>3 heavy smokers
**Canullo**	2014	RCT (unicenter)	Spain / University	15 (6/9)	32-76 (57.3)	Delayed (2 mo)	Md	Premium SP (Sweden & Martina, Padua, Italy)	4 light smokers
**Cappare**	2019	PS (unicenter)	Italy / University	24 (NM)	NM	Immediate	Mx, Md	TTx, Winsix (Biosafin, Ancona, Italy)	12 smokers
**Carr**	2019	RA (unicenter)	USA / Non-profit organization	2798 (NM)	>18	Immediate, delayed	Mx, Md	NM	NM
**Cavalcanti**	2011	RA (multicenter)	Italy / Private practice	1727 (702/1025)	17-85 (47-51)	Immediate, delayed	Mx, Md	Several (3i Biomet, Astra Tech, Camlog, Friadent-Dentsply, Nobel Biocare, Straumann, Sweden & Martina, Zimmer Dental)	549 smokers
**Cha**	2014	PS (unicenter)	South Korea / Hospital	161 (96/65)	NM	Delayed (6 mo)	Mx	Implantium (Dentium Co., Seoul, Korea)	18 heavy and extra-heavy smokers
**Chang**	2020	RA (unicenter)	China / Hospital	376 (222/154)	NM (49)	Delayed	Mx, Md	Osseotite, Certain Taper (Biomet 3i, Palm Beach Gardens, USA)	15 smokers<br>52 former smokers
**Chiapasco (1)**	2018	RA (NM)	Italy / NM	69 (16/53)	16-72 (48)	Delayed (3-9 mo)	Mx, Md	NM	10 light smokers
**Chiapasco (2)**	2020	RA (NM)	Italy / NM	75 (21/54)	18-78 (49)	Delayed (3-10 mo)	Md	NM	10 light smokers
**Chrcanovic (1)**	2017	RA (unicenter)	Sweden / Public service	999 (479/520)	14-90 (60)	Immediate, delayed	Mx, Md	Several	263 light, heavy and extra-heavy smokers<br>23 former smokers
**Chrcanovic (2)**	2017	RA (unicenter)	Sweden / Public service	1406 (625/781)	NM	Immediate, delayed	Mx, Md	Several	285 light, heavy and extra-heavy smokers<br>33 former smokers
**Chrcanovic (3)**	2018	RA (unicenter)	Sweden / Public service	227 (95/132)	NM	Immediate, delayed	Mx, Md	Several	Light, heavy and extra-heavy smokers
**Clauser**	2020	PS (multicenter)	Italy / Private practice	214 (92/122)	17-84 (48.3)	Immediate	Mx, Md	NanoTite Certain Tapered (Biomet 3i)	24 heavy smokers<br>4 extra-heavy smokers

**Conrad**	2011	RA (unicenter)	USA / University	NM	18-84 (55.3)	Immediate, delayed (mean 3.8 mo)	Mx	NM	NM
**Corvino**	2020	RCT (unicenter)	Italy / Private practice	33 (17/16)	NM (67.4)	Delayed (2-3 mo)	Mx, Md	NeO Conical Standard, NeO Internal Hex (Alpha-Bio Tec, Petah Tikva, Israel)	5 light smokers
**Coskunses**	2021	PS (unicenter)	Turkey / University	28 (17/11)	23-72 (52)	Immediate	Mx, Md	Roxolid Bone Level Tapered (Straumann, Basel, Switzerland)	6 smokers
**Crespi**	2019	RA (NM)	Italy / NM	22 (8/14)	NM (54)	Immediate, delayed (4 mo)	Mx	out-Link PRO-Link (Sweden & Martina)	3 smokers
**Cristalli**	2015	PS (NM)	Italy / NM	24 (9/15)	35-65 (47.3)	Immediate	Mx, Md	NobelActive (Nobel Biocare, Göteborg, Sweden)	6 light smokers
**Cucchi**	2019	PS (NM)	Italy / NM	20 (NM)	NM (67)	Early (3-5 d)	Mx, Md	NM	Light smokers
**D'haese**	2013	PS (unicenter)	Belgium / University	26 (16/10)	20-81 (51.8)	Immediate	Mx	Osseospeed (Astra Tech, Mölndal, Sweden)	9 heavy smokers
**Daher**	2019	RCT (unicenter)	Lebanon / University	24 (11/13)	34-67 (49.2)	Immediate, delayed	Mx	NobelActive (Nobel Biocare, Göteborg, Sweden)	13 heavy smokers
**Daneshvar**	2016	RA (unicenter)	Canada / University	111 (40/71)	17-86 (56.1)	NM	Mx, Md	NM	8 smokers
**Daubert**	2015	RA (unicenter)	USA / University	96 (48/48)	31-86 (67.6)	Immediate, delayed	Mx, Md	Several	7 smokers, but the degree of smoking among the patients was not reported
**De Bruyn**	1994	RA (unicenter)	Belgium / Private practice	117 (NM)	20-80 (NM)	NM	Mx, Md	Brånemark (Nobel Biocare, Göteborg, Sweden)	26 smokers
**De Bruyn**	1999	PS (unicenter)	Sweden / University	23 (NM)	NM	NM	Mx	Screw-Vent (Zimmer Dental, Carlsbad, USA)	10 smokers
**Degidi**	2015	PS (unicenter)	Italy / Private practice	114 (NM)	NM (53.1)	Immediate	Mx, Md	XiVE (Dentsply, Mannheim, Germany)	34 smokers
**Deli**	2014	RA (unicenter)	Italy / University	216 (94/122)	NM (54)	Delayed (4 mo)	Mx, Md	Straight line (Dentegris, Duisburg, Germany)	92 light smokers
**Di Stefano**	2020	RA (multicenter)	Italy / Private practice	32 (12/20)	36-73 (52.2)	Delayed (3-4 mo)	Mx, Md	XiVE (Dentsply)	9 light smokers
**Doan**	2014	RA (multicenter)	Australia / University	472 (216/256)	19-89 (54)	Delayed	Mx, Md	Several (Imtec, Innova, MIS, Southern, Straumann, Biomet/3i, Zimmer Dental Implant Systems)	39 smokers
**Donati (1)**	2015	PS (multicenter)	Italy / Private practice	151 (70/81)	NM (45)	Immediate, delayed (3 mo)	Mx, Md	Osseospeed (Astra Tech, Mölndal, Sweden)	34 smokers
**Donati (2)**	2018	RCT (unicenter)	Sweden / Public service	51 (20/31)	36-80 (59.5)	Delayed	Mx, Md	Turned and TiOblast (Astra Tech, Mölndal, Sweden)	17 smokers, but the degree of smoking among

									the patients was not reported
**Donos**	2019	RCT (unicenter)	England / University	16 (5/11)	NM (49)	Immediate, delayed (4 mo)	Mx	Straumann Bone Level SLActive (Straumann, Basel, Switzerland)	3 light smokers
**Doyle**	2007	RA (unicenter)	USA / Private practice	171 (NM)	NM (47.5)	NM	Mx, Md	NM	10 smokers
**Engstrand**	2003	PS (unicenter)	Sweden / Public Service	95 (53/42)	45-89 (68.5)	Immediate, delayed	Md	Turned (Brånemark, Nobel Biocare, Göteborg, Sweden)	23 smokers
**Feher**	2020	RA (unicenter)	Austria / University	1132 (505/627)	NM (50.6)	Evaluation of implants before loading	Mx, Md	NM	157 light smokers<br>60 heavy smokers
**Felice**	2015	RCT (multicenter)	Italy / Private practice	50 (25/25)	32-72 (52)	Immediate, delayed (4 mo)	Mx	XiVE S plus (Dentsply Friadent, Mannheim, Germany)	Light and heavy smokers
**Flores-Guillen**	2018	RCT (unicenter)	Spain / University	40 (23/17)	NM (48)	Delayed (2-3 mo)	Mx, Md	Straumann Bone Level SLActive (Straumann, Basel, Switzerland)	12 light smokers
**Fonseca**	2021	PS (unicenter)	Switzerland / University	32 (20/12)	28-82 (52.4)	Early, delayed (2-4 mo)	Mx, Md	SLActive (Straumann, Basel, Switzerland)	5 smokers<br>3 former smokers
**Francetti (1)**	2014	PS (unicenter)	Italy / Private practice	22 (10/22)	NM	Delayed (3-6 mo)	Mx, Md	Replace Select Straight and Tapered TiUnite (Nobel Biocare, Göteborg, Sweden)	6 light and heavy smokers
**Francetti (2)**	2019	RA (unicenter)	Italy / Private practice	77 (32/45)	NM (68.2)	Immediate, early	Mx, Md	Nobel Speedy Groovy, MK IV TiUnite (Nobel Biocare, Göteborg, Sweden)	19 light and heavy smokers
**Friberg**	2008	RA (unicenter)	Sweden / Public Service	75 (39/36)	20-87 (64)	NM	Mx	Turned (Brånemark, Nobel Biocare, Göteborg, Sweden)	28 smokers
**Gamper**	2017	RCT (unicenter)	Switzerland / University	60 (23/37)	NM (47.8-55.7, 2 groups)	Early, delayed (from 6 wk to 7 mo)	Mx, Md	Mk III and Mk IV TiUnite (Nobel Biocare, Göteborg, Sweden), SLA Standard, Standard Plus, and Standard Plus Tapered (Straumann, Basel, Switzerland)	14 smokers, 2 smoked <5 cig/day and 12 smoked >5 cig/day
**Gander**	2014	RA (multicenter)	Switzerland / Private practice, University	33 (23/10)	NM (64)	NM	Md	OsseoSpeed (Astra Tech, Mölndal, Sweden)	21 smokers
**Gaspersic**	2021	PS (unicenter)	Slovenia / NM	11 (5/6)	49-73 (61)	Delayed (6 mo)	Mx	SLActive (Straumann, Basel, Switzerland)	3 smokers
**Geurs**	2001	RA (multicenter)	USA / Private practice	100 (NM)	NM	NM	Mx	NM	NM
**Ghazal**	2019	RCT (multicenter)	USA / University	50 (18/32)	NM (51.2)	Early (3-4 wk)	Mx, Md	Roxolid (Straumann, Basel, Switzerland)	2 light smokers<br>6 former smokers
**Gherlone (2)**	2016	PS (unicenter)	Italy / Hospital	68 (22/46)	40-73 (55.3)	Delayed (2-3 mo)	Mx, Md	WinSix (BioSAFin., Ancona, Italy)	29 light smokers<br>13 heavy smokers

**Gjelvold**	2020	PS (unicenter)	Sweden / Public service	46 (25/21)	NM (40)	Immediate	Mx	Tapered Internal (BioHorizons, Birmingham, USA)	3 smokers, but the degree of smoking among the patients was not reported
**Gorman**	1994	CCT (multicenter)	USA / Military clinic	310 (NM)	NM	NM	Mx, Md	NM	82 smokers
**Göthberg**	2018	RCT (unicenter)	Sweden / Public service	50 (18/32)	NM (67)	Immediate, delayed (3 mo)	Mx, Md	Brånemark TiUnite (Nobel Biocare, Göteborg, Sweden)	15 light smokers
**Grandi (1)**	2012	CCT (multicenter)	Italy / Private practice	47 (22/25)	52-78 (62.3)	Immediate	Md	JDEvolution (JDentalCare, Modena, Italy)	11 light and heavy smokers
**Grandi (2)**	2012	RCT (multicenter)	Italy / Private practice	28 (11/17)	39-64 (51)	Immediate	Mx, Md	JDEvolution (JDentalCare, Modena, Italy)	9 light smokers
**Grandi (3)**	2012	PS (multicenter)	Italy / Private practice	42 (13/29)	71-89 (76.5)	Immediate	Md	JDEvolution (JDentalCare, Modena, Italy)	9 light smokers
**Grandi (4)**	2013	RCT (multicenter)	Italy / Private practice	80 (31/49)	39-65 (52-55)	Immediate, delayed (2 mo)	Mx, Md	JDEvolution (JDentalCare, Modena, Italy)	22 light smokers
**Grandi (5)**	2014	RCT (multicenter)	Italy / Private practice	25 (9/16)	39-74 (56)	Immediate	Mx, Md	JDEvolution (JDentalCare, Modena, Italy)	9 light and heavy smokers
**Grandi (6)**	2015	RCT (multicenter)	Italy / Private practice	105 (50/55)	21-75 (45-51)	Immediate, early, delayed (4 mo)	Mx, Md	JD Evolution (J DentalCare, Modena, Italy)	19 light smokers<br>6 heavy smokers
**Grisar**	2017	RA (unicenter)	Belgium / University	509 (240/269)	16-87 (58)	Immediate, delayed	Mx, Md	Several (Nobel Biocare, Straumann, Bicon, Astra, Ankylos)	Yes, but exact number not reported
**Groenendijk**	2020	PS (multicenter)	Netherlands / Private practice + University	98 (41/57)	17-80 (45.8)	Immediate	Mx	NobelActive (Nobel Biocare, Göteborg, Sweden)	12 smokers
**Grossmann**	2008	RA (unicenter)	Israel / Private practice	23 (20/3)	33-57 (44.2)	NM	Mx, Md	Several (Zimmer Dental, 3i Implant Innovations, MIS Implants Technologies)	8 smokers
**Grunder**	1999	PS (multicenter)	Switzerland / Private practice	74 (40/34)	NM (57.8)	Delayed (3-6 mo)	Mx, Md	Osseotite (3i-Implant Innovations Inc., Palm Beach Gardens, USA)	19 smokers
**Guarnieri (1)**	2019	RCT (unicenter)	Italy / University	20 (12/8)	36-64 (49.7)	Delayed (4-6 mo)	Mx, Md	Laser Lok (BioHorizons, Birmingham, USA)	Light smokers
**Guarnieri (2)**	2020	RA (multicenter)	Italy / Private practice	274 (146/128)	45-75 (48.1)	Immediate, delayed	Mx, Md	BioHorizons TLX and TRX (BioHorizons)	124 smokers
**Guida**	2020	RCT (multicenter)	Italy / Private practice + University	30 (17/13)	NM (63)	Delayed (3 mo)	Md	OsseoSpeed TX (Dentsply, Mölndal, Sweden)	7 heavy smokers<br>4 former smokers

**Habsha**	2000	RA (unicenter)	Canada / University	389 (NM)	15-84 (49.3)	NM	Mx, Md	Brånemark (Nobel Biocare, Göteborg, Sweden)	104 smokers
**Hakam**	2021	RA (unicenter)	USA / University	771 (346/425)	22-97 (67.3)	NM	Mx, Md	NM	37 smokers
**Han**	2018	PS (multicenter)	China / University	45 (17/28)	26-73 (53)	Early (6 wk)	Mx, Md	OsseoSpeed TX (Dentsply, Mölndal, Sweden)	2 smokers<br>3 former smokers
**Hartlev**	2021	RCT (unicenter)	Denmark / University	27 (15/12)	23-72 (50)	Delayed (mean 6.7 mo)	Mx	NobelParallel Conical Connection (Nobel Biocare, Göteborg, Sweden)	3 smokers
**Hattingh (1)**	2018	PS (unicenter)	England / Private practice	51 (36/15)	38-89 (61)	Delayed (4 mo)	Mx, Md	MAX (Southern Implants, Irene, South Africa)	5 smokers
**Hattingh (2)**	2019	RA (unicenter)	United Kingdom / Private practice	85 (43/42)	45-87 (65)	Delayed (4 mo)	Mx, Md	MAX (Southern Implants, Irene, South Africa)	NM
**He**	2015	RA (unicenter)	China / University	1377 (704/673)	18-83 (44.9)	Immediate, delayed	Mx, Md	Several (Straumann, Bego, Dentium, Anthogyr, Biomet 3i, Lifecore, Osstem, BLB)	NM
**Higuchi**	2020	PS (multicenter)	USA, Chile, Spain, Australia, Italy / University + Private practice	110 (50/60)	NM (61.7)	Immediate, early (within 10 d)	Md	Nobel Active (Nobel Biocare, Göteborg, Sweden)	23 light and heavy smokers<br>47 former smokers
**Hingsammer**	2018	RA (unicenter)	Austria / Private practice	25 (9/16)	19-73 (42.5)	Immediate	Mx	NM (Nobel Biocare, Göteborg, Sweden)	3 smokers
**Hinze**	2010	CCT (unicenter)	Germany / Private practice	37 (18/19)	39-84 (64.6)	Immediate	Mx, Md	Osseotite, NanoTite Tapered (Biomet 3i, Palm Beach Gardens, USA)	11 light and heavy smokers
**Hof**	2014	RA (unicenter)	Austria / University	153 (73/80)	NM (37)	Delayed	Mx	MkIII, Nobel Replace Tapered (Nobel Biocare, Göteborg, Sweden)	15 smokers
**Holahan**	2008	RA (unicenter)	USA / University	192 (0/192)	50-85 (63.4)	NM	Mx, Md	NM	24 smokers
**Horwitz**	2012	PS (unicenter)	Israel / University	18 (NM)	34-69 (54.5)	Immediate	Mx, Md	Seven (MIS Implants Technologies Ltd., Tel Aviv, Israel)	5 smokers
**Hsu**	2016	RCT (unicenter)	USA / University	26 (19/7)	31-90 (57.7)	Early	Mx	NM (SuperLine, Dentium), Zimmer TSV (Zimmer Dental)	3 smokers<br>10 former smokers

**Hu**	2020	RA (unicenter)	Singapore / Public Service	200 (100/100)	28-84 (57.7)	NM	Mx, Md	NM (Straumann, Basel, Switzerland)	Light smokers
**Ibañez (1)**	2003	CCT (NM)	Argentina / Private practice	195 (86/109)	18-79 (49.2)	Immediate, delayed	Mx, Md	Osseotite (3i-Implant Innovations Inc., Palm Beach Gardens, USA)	Heavy smokers
**Ibañez (2)**	2005	PS (multicenter)	Argentina / Private practice, University	41 (11/30)	38-82 (62.1)	Immediate	Mx, Md	Osseotite (Biomet 3i, Palm Beach Gardens, USA)	NM
**Iorio-Siciliano**	2016	RA (unicenter)	Italy / University	20 (7/13)	NM (46.5)	Delayed (3-6 mo)	Mx, Md	Laser-Lok Tapered Internal Plus (BioHorizons)	6 light smokers
**Ji**	2012	RA (unicenter)	USA / University	45 (18/27)	25-88 (61.5)	Immediate	Mx, Md	XiVe (Dentsply Friadent, Mannhein, Germany), TiUnite (Nobel Biocare, Göteborg, Sweden), Steri-Oss Hex-Loc (Nobel Biocare, Göteborg, Sweden) Tapered Screw-Vent, (Zimmer Dental, Carlsbad, USA)	8 smokers
**Jones**	1999	RCT (unicenter)	USA / University	65 (25/40)	21-70 (50)	Delayed (3-7 mo)	Mx, Md	TPS (Sterngold/Implamed, Attleboro, USA)	19 smokers
**Kan**	1999	RA (unicenter)	USA / University	60 (27/33)	41-84 (64.6)	NM	Mx	NM	16 smokers
**Karoussis**	2003	CCT (unicenter)	Switzerland / University	53 (NM)	NM	Delayed (4-6 mo)	Mx, Md	TPS (ITI Straumann, Waldenburg, Switzerland)	12 smokers
**Keller**	1999	RA (unicenter)	USA / Non-profit organization	62 (42/20)	15-73 (28-59)	NM	Mx	Brånemark (Nobel Biocare, Göteborg, Sweden)	8 smokers
**Khouly**	2017	RA (unicenter)	Spain / Private practice	67 (30/37)	NM (56)	Delayed (6-9 mo)	Mx	BTI Externa (BTI, Biotechnology Institute, Vitoria-Gasteiz, Spain) and Brånemark Mk IV TiUnite (Nobel Biocare, Göteborg, Sweden)	NM
**Kim**	2015	RA (unicenter)	USA / Community practice	85 (43/42)	17-83 (55)	Immediate, delayed	Mx, Md	Nobel Replace Select (Nobel Biocare, Göteborg, Sweden)	NM
**Kinsel**	2007	RA (unicenter)	USA / Private practice	43 (12/31)	35-80 (58)	Immediate	Mx, Md	TPS, SLA (Straumann, Basel, Switzerland)	12 extra-heavy smokers
**Klotz**	2019	RA (unicenter)	Germany / Private practice	84 (28/56)	28-96 (60.2)	Delayed	Mx, Md	BlueSky (Bredent Medical)	Yes, but the exact number was not reported

**Kolerman**	2017	RA (unicenter)	Israel / University	39 (16/23)	24-82 (47.5)	Immediate	Mx	Lans (MIS, Bar Lev, Israel)	7 light smokers
**Komiyama**	2008	RA (unicenter)	Sweden / University	29 (20/9)	42-90 (71.5)	Immediate	Mx, Md	TiUnite Brånemark MkIII (Nobel Biocare, Göteborg, Sweden)	5 smokers
**Krennmair (1)**	2016	CCT (unicenter)	Austria / University	41 (21/20)	NM (62.9)	Delayed (2 mo)	Md	Camlog Screw-line (Promote+, Wimsheim, Germany)	5 light smokers
**Krennmair (2)**	2019	PS (unicenter)	Austria / Private practice	85 (39/46)	NM (56.7)	Delayed (5-7 mo)	Mx	Camlog Screw-line (Promote+, Wimsheim, Germany)	15 smokers
**Kumar**	2002	RA (unicenter)	USA / Private Practice	461 (NM)	NM	Delayed (6-12 wk)	Mx, Md	Sandblasted and acid-etched (SLA, ITI, Straumann, Waldenburg, Switzerland)	72 heavy smokers
**Lambert**	2000	CCT (multicenter)	USA / Public Hospital	662 (NM)	NM	NM	Mx, Md	NM	199 smokers
**Le**	2013	RA (unicenter)	USA / Private practice	168 (74/94)	34-87 (61)	NM	Mx, Md	SLA (Straumann, Waldenburg, Switzerland), Astra Tech (AstraTech, Mölndal, Sweden), NM (Zimmer Dental, Warsaw, USA), NM (3i Implant Innovations, Palm Beach Gardens), NM (BioHorizons, Birmingham, USA)	13 smokers
**Lee (1)**	2018	RA (unicenter)	Taiwan / Private practice	161 (NM)	23-90 (53)	Immediate, delayed	Mx, Md	IDEOSS (IDEOSS Biotech, Taipei, Taiwan)	Yes, but the exact number was not reported
**Lee (2)**	2020	RCT (unicenter)	USA / University	36 (13/23)	NM (49.2)	Immediate	Mx	Full OSSEOTITE Certain Tapered Implant (Biomet 3i, Palm Beach Gardens, USA)	2 light smokers
**Leventi**	2014	RA (unicenter)	Scotland / University	41 (16/25)	45-88 (65.4)	Delayed (>3 mo)	Md	NM	9 smokers
**Levin (1)**	2008	RA (unicenter)	Israel / Private practice	55 (NM)	18-78 (45)	NM	Mx, Md	NM	6 smokers
**Levin (2)**	2011	CCT (unicenter)	Israel / Private practice	717 (273/444)	NM (46-54)	NM	Mx, Md	NM	103 heavy smokers
**Lin**	2012	RA (multicenter)	USA / University	75 (32/43)	NM (59.6)	Delayed (1 mo)	Mx	NM	28 smokers
**Lindquist**	1997	CCT (unicenter)	Sweden / University	45 (13/32)	33-64 (65)	Delayed (4 mo)	Md	Brånemark (Nobel Biocare, Göteborg, Sweden)	21 light, heavy, and extra-heavy smokers
**Lini**	2019	RA (unicenter)	Italy / University	67 (45/22)	NM (65)	Delayed (2-6 mo)	Mx, Md	Frialit-2 (Friadent)	NM
**Lobato**	2020	RCT (unicenter)	Brazil / University	44 (NM)	25-77 (50.8)	Delayed (4-6 mo)	Mx, Md	Alvim, Drive, and Titamax (Neodent, Curitiba, Brazil)	Light and heavy smokers

**Locante**	2004	RA (unicenter)	USA / Private practice	86 (35/51)	12-81 (NM)	Immediate	Mx	Stabledent (Crystal Medical Technology, Pelham, USA)	9 light, heavy, and extra-heavy smokers
**Lombardo**	2020	RA (unicenter)	NM / University	98 (43/55)	32-77 (54.1)	Delayed (4-6 mo)	Md	Morse taper, Morse cone (Bicon Dental Implants, Boston, USA)	20 smokers
**Luongo**	2020	PS (NM)	NM / NM	190 (97/93)	NM (64.2)	Delayed (6-7 mo)	Mx	T3 (Biomet 3i)	42 smokers
**Malchiodi (1)**	2014	PS (NM)	Italy / University	136 (46/90)	48-71 (58.9)	Delayed (3-4 mo)	Mx, Md	Endopore Dental System (Innova Corporation, Toronto, Canada)	32 light smokers<br>19 heavy smokers
**Malchiodi (2)**	2016	RCT (unicenter)	Italy / University	40 (24/16)	35-75 (52)	Delayed (3 mo)	Mx, Md	SybronPRO XR (Sybron Implant Solutions)	10 light and heavy smokers
**Maló (1)**	2013	RA (unicenter)	Portugal / Private clinic	70 (29/41)	35-81 (54)	Immediate	Mx	NobelSpeedy Replace and Shorty (Nobel Biocare, Göteborg, Sweden)	NM
**Maló (2)**	2019	RA (unicenter)	Portugal / Private practice	1072 (442/630)	20-88 (55.8)	Immediate	Mx	MK III, MK IV, NobelSpeedy Groovy (Nobel Biocare, Göteborg, Sweden)	241 smokers
**Maló (3)**	2019	RA (unicenter)	Portugal / Private practice	471 (185/286)	20-85 (57.7)	Immediate	Md	MK II, MK III, MK IV, NobelSpeedy Groovy (Nobel Biocare, Göteborg, Sweden)	117 smokers
**Mastrangelo**	2018	RCT (multicenter)	Italy / Private practice	102 (63/39)	18-72 (44)	Delayed (4 mo)	Mx	NM (tioLogic Implant System, Dentaurum, Germany)	28 smokers, but the degree of smoking among the patients was not reported
**Mayfield**	2001	RA (unicenter)	Sweden / Private practice	15 (7/8)	47-80 (71)	NM	Mx, Md	Several (Nobel Biocare, ITI, Cresco IMZ)	3 smokers
**McCarthy**	2003	RA (unicenter)	UK / University	18 (6/12)	18-75 (43.7)	Delayed (mean 35.4 wk)	Mx	Brånemark (Nobel Biocare, Göteborg, Sweden)	5 smokers
**Mendonça**	2014	RA (unicenter)	Brazil / Private practice	198 (86/112)	45-81 (60)	Delayed (12-18 wk)	Mx, Md	NM	NM
**Merli (1)**	2017	RCT (unicenter)	Italy / Private practice	24 (9/15)	47-76 (63.4)	NM	Mx, Md	NM (Nobel Biocare, Göteborg, Sweden)	5 light and heavy smokers
**Merli (2)**	2020	RCT (unicenter)	Italy / Private practice	60 (22/38)	19-72 (49)	Immediate, early	Mx, Md	Element, Contact (Thommen Medical, Waldenburg, Switzerland)	7 smokers
**Mertens**	2012	PS (unicenter)	Germany / University	15 (5/10)	41-69 (55.3)	Delayed (6 mo)	Mx	TiOblast (Astra Tech, Mölndal, Sweden)	6 smokers
**Migliorati**	2015	RCT (unicenter)	Italy / University	48 (23/25)	22-70 (47.5)	Immediate	Mx	Tapered Effect, Bone Level SLActive (Straumann, Basel, Switzerland)	2 light smokers
**Mijiritsky (1)**	2013	RA (multicenter)	Israel / Private practice	787 (NM)	18-86 (53.7)	Immediate, delayed	Mx, Md	NM (Adin Dental Implants, Alon Tavor, Israel).	NM

**Mijiritsky (2)**	2015	RA (NM)	Israel / NM	20 (13/7)	44-67 (56)	NM	Mx, Md	Several (Zimmer Dental, Carlsbad, USA; Friadent, Mannheim, Germany; MIS Implants Technologies, Shlomi, Israel)	4 smokers
**Mijiritsky (3)**	2016	RA (multicenter)	Israel, Romania, Italy, Canada / University + Private practice	37 (22/15)	37-75 (51.2)	Delayed	Mx	iRaise (Maxillent Ltd, Herzliya, Israel)	2 smokers
**Minsk**	1996	RA (unicenter)	USA / University	380 (NM)	NM	NM	Mx, Md	Brånemark (Nobel Biocare, Göteborg, Sweden), others (ITI, 3i, IMZ, Astra)	NM
**Mir-Mari**	2012	RA (unicenter)	Spain / Private practice	68 (24/44)	NM (45.1)	Delayed (4-6 mo)	Mx, Md	NM	21 smokers
**Moheng**	2005	PS (unicenter)	France / Hospital	93 (36/57)	18-85 (60.5)	NM	Mx, Md	Frialit-2 (Friadent, Mannheim, Germany), IMZ Twin Plus (Friadent, Mannheim, Germany)	15 smokers
**Moraguez**	2016	PS (unicenter)	Switzerland / University	10 (4/6)	32-68 (49.4)	Delayed	Mx	NM (Straumann, Basel, Switzerland)	2 light smokers
**Morales-Vadillo**	2013	RA (unicenter)	Brazil / Private practice	154 (NM)	20-87 (55)	NM	Mx, Md	Bioform (BiomacMed, Juiz de Fora, Brazil)	NM
**Mordenfeld**	2014	PS (unicenter)	Sweden / Public service	20 (6/14)	48-69 (62)	Delayed (6-9 mo)	Mx	Mk II (Nobel Biocare, Göteborg, Sweden)	9 smokers
**Mundt**	2006	RA (unicenter)	Germany / Private practice	159 (65/94)	14.9-80.9 (54.1)	NM	Mx, Md	Tiolox implants (Dentaurum, Ispringen, Germany)	NM
**Munoz**	2021	RCT (unicenter)	Spain / University	65 (32/33)	NM (54.9)	Delayed (10 wk)	Mx, Md	Vega (Klockner, Spain)	8 smokers
**Naeini**	2018	CCT (unicenter)	England / Private practice	36 (20/16)	28-85 (62)	Delayed	Mx, Md	Brånemark TiUnite (Nobel Biocare, Göteborg, Sweden)	5 smokers
**Nedir**	2004	CCT (unicenter)	Switzerland / Private practice	236 (91/145)	18-89 (NM)	Delayed	Mx, Md	TPS, SLA, ITI (Straumann, Waldenburg, Switzerland)	Light and heavy smokers
**Niedermaier**	2017	RA (unicenter)	Germany / Private practice	380 (188/192)	23-92 (61.9)	Immediate	Mx, Md	NanoTite (Biomet 3i, Palm Beach Gardens, USA), Nobel Active, Nobel Speedy, Nobel Speedy Replace and Nobel Replace CC (Nobel Biocare, Göteborg, Sweden)	141 smokers

**Nitzan**	2005	RA (unicenter)	Israel / Private practice	161 (NM)	23-89 (57)	NM	Mx, Md	NM	59 light and heavy smokers
**Noelken**	2020	RCT (unicenter)	Germany / Private practice	50 (18/32)	23-73 (47)	Delayed (>3 mo)	Mx, Md	OsseoSpeed (Astra Tech, Mölndal, Sweden)	7 light smokers<br>1 heavy smoker
**Nogueira**	2018	PS (unicenter)	Brazil / University	45 (11/34)	48-80 (63.4)	Delayed (3 mo)	Md	Titamax (Neodent, Curitiba, Brazil)	5 light smokers
**Noguerol**	2006	RA (unicenter)	Spain / Private practice	316 (NM)	NM	NM	Mx, Md	Brånemark (Nobel Biocare, Göteborg, Sweden)	Light, heavy, and extra-heavy smokers
**Norton**	2017	PS (unicenter)	United Kingdom / Private practice	22 (10/12)	22-79 (NM)	Immediate	Mx, Md	Astra Tech AV (Dentsply, Mölndal, Sweden)	Light smokers
**Nyström**	2009	PS (unicenter)	Sweden / University	26 (13/13)	38-70 (54.7)	Delayed (4-8 wk)	Mx	Brånemark (Nobel Biocare, Göteborg, Sweden)	6 smokers
**Oliva**	2012	RA (unicenter)	Spain / Private practice	17 (11/6)	NM (52.88)	Delayed (4 mo)	Mx, Md	SLA, ITI (Straumann, Waldenburg, Switzerland), SSII (Osstem, Surrey, UK)	8 smokers
**Olmedo-Gaya**	2016	RA (unicenter)	Spain / Private practice	142 (52/90)	20-78 (48.5)	Delayed	Mx, Md	BTI (Biotechnology Institute)	37 smokers
**Olson**	2000	RA (multicenter)	USA / University, Public institution	29 (28/1)	34-78 (56)	NM	Mx	NM	NM
**Omran**	2015	RA (unicenter)	USA / University	NM	≥18	NM	Mx, Md	Several (Astra Tech Osseospeed, Biomet 3i, NobelReplace)	Yes, but exact number not informed
**Park**	2019	RA (unicenter)	South Korea / Private practice	207 (137/70)	35-70 (51.1)	Delayed (6 mo)	Mx	NM	98 smokers
**Patil**	2020	RCT (unicenter)	Malaysia / University	24 (10/14)	NM (64)	Immediate	Md	Roxolid (Straumann, Basel, Switzerland)	1 heavy smoker
**Peleg**	2006	CCT (NM)	USA, Israel / NM	731 (278/453)	42-81 (53)	Delayed (6-9 mo)	Mx	Spline HA cylinder MP-1 (Zimmer Dental, Carlsbad, USA), MTX (Zimmer Dental, Carlsbad, USA)	226 smokers
**Penarrocha**	2014	RA (unicenter)	Spain / University	13 (5/8)	30-76 (54.8)	Delayed (3 mo)	Mx	Phibo Avantblast (PhiboDental Solutions S.L., Barcelona, Spain)	13 light smokers
**Penarrocha-Oltra**	2019	RCT (multicenter)	Spain / University + Private practice	20 (7/13)	23-70 (51)	Delayed (4 mo)	Mx	Ticare Inhex (MozoGrau, Valladolid, Spain)	3 light smokers<br>4 heavy smokers

**Perez**	2020	RCT (unicenter)	Switzerland / University	36 (19/17)	23-77 (51-59)	Delayed (4 mo)	Mx, Md	BLT (Straumann, Basel, Switzerland)	8 light smokers
**Pico**	2019	RCT (unicenter)	Spain / University	33 (NM)	40-76 (54)	Delayed (3 mo)	Mx, Md	BioniQ (LASAK)	NM
**Pozzi (1)**	2014	RCT (unicenter)	Italy / University	34 (15/19)	39-59 (52.2)	Delayed (2 mo)	Md	NobelActive and Nobel Speedy Groovy (Nobel Biocare, Göteborg, Sweden)	4 light smokers
**Pozzi (2)**	2014	PS (multicenter)	Italy, USA / Private practice	66 (28/38)	39-79 (51.3)	Immediate	Mx	Nobel Speedy Replace and Groovy (Nobel Biocare, Göteborg, Sweden)	16 light smokers
**Pozzi (3)**	2015	RA (unicenter)	Italy / University	22 (11/11)	50-83 (68.3)	Immediate	Mx, Md	TiUnite (Nobel Biocare, Göteborg, Sweden)	3 light smokers
**Pozzi (4)**	2016	PS (unicenter)	Italy / University	18 (7/11)	58-73 (65.4)	Delayed (3 mo)	Mx, Md	Nobel Replace (Nobel Biocare, Göteborg, Sweden)	2 light smokers
**Prati**	2020	PS (multicenter)	Italy / University + Private practice	76 (34/42)	NM (55.6)	Delayed (3 mo)	Mx, Md	Premium SP (Sweden & Martina, Due Carrare, Italy)	8 heavy smokers
**Queridinha**	2016	RA (unicenter)	Portugal / Private practice	60 (21/39)	NM (64.1)	Immediate	Mx	Mk II, Mk III, Mk IV, TiUnite, NobelSpeedy Groovy, NobelReplace Tapered (Nobel Biocare, Göteborg, Sweden)	NM
**Raabe**	2021	RA (unicenter)	Switzerland / University	55 (18/37)	26-87 (60.8)	Delayed (>2 mo)	Mx, Md	SLA, SLActive (Straumann, Basel, Switzerland)	2 light smokers<br>3 heavy smokers
**Raes (1)**	2015	CCT (multicenter)	Belgium, Italy / Private practice + University	85 (42/43)	NM	Immediate	Mx	Osseospeed (Astra Tech, Mölndal, Sweden)	46 smokers
**Raes (2)**	2018	PS (multicenter)	Italy, Belgium / Private practice + University	46 (23/23)	18-73 (44.5)	Immediate	Mx, Md	AnyRidge (MegaGen Implant, Gyeongbuk, South Korea)	17 smokers
**Ramaglia**	2015	PS (unicenter)	Italy / University	21 (13/8)	30-53 (39)	Delayed (4 mo)	Mx	NM (Biomet 3i, Palm Beach, USA)	8 smokers
**Rasperini**	2014	RA (multicenter)	Italy / University	120 (NM)	NM (46-51)	Delayed (4-6 mo)	Mx, Md	Brånemark machined (Nobel Biocare, Göteborg, Sweden), TPS (Straumann, Basel, Switzerland)	40 smokers
**Ravida (1)**	2018	RA (unicenter)	USA / University	45 (24/21)	22-83 (58.9)	Immediate, delayed	Mx, Md	Several	5 smokers
**Ravida (2)**	2019	RA (unicenter)	USA / University	145 (64/81)	NM (69.7)	NM	Mx, Md	NM	39 smokers

**Roccuzzo**	2016	CCT (unicenter)	Italy / Private practice	128 (52/76)	NM (52.4)	Delayed (6-10 wk)	Md	SLA (Straumann, Basel, Switzerland)	21 smokers
**Rodriguez-Argueta**	2011	RA (unicenter)	Spain / University	295 (127/168)	21-68 (53.1)	NM	Mx, Md	Brånemark, Replace (Nobel Biocare, Göteborg, Sweden), DEFCON Tissue Care (Impladent SL, Sentmenat, Spain), NM (AstraTech, Mölndal, Sweden)	113 smokers
**Romandini**	2019	RA (unicenter)	Italy / University	52 (24/28)	NM (68.5)	NM	Mx, Md	Several (Straumann, Biomet 3i, Nobel Biocare, Camlog)	10 smokers<br>4 former smokers
**Romanos (1)**	2013	CCT (unicenter)	Germany / University	20 (12/8)	NM (55.7, G1)<br>NM (65.9, G2)	Immediate	Mx, Md	Ankylos (Dentsply Implants, Mannheim, Germany)	8 heavy smokers
**Romanos (2)**	2014	PS (unicenter)	Germany / University	13 (7/6)	NM (60.8)	Immediate	Md	Ankylos (Dentsply implants, Mölndal, Sweden)	5 heavy smokers
**Rosen (1)**	1999	RA (multicenter)	USA / Private practice	101 (37/64)	31-81 (56.1)	Delayed (mean 7 mo)	Mx	Several (Dentsply Standard; Implamed standard; TPS cylinders, Implant Innovations; standard, Implant Innovations; Interpore TPS cylinders, Wurmberg, Germany; turned, Nobel Biocare, Göteborg, Sweden; TPS, Straumann Waldenburg, Switzerland)	17 smokers
**Rosen (2)**	2018	RA (unicenter)	USA / Private practice	75 (27/48)	29-88 (61)	Delayed (2-6 mo)	Mx, Md	ProActive (Neoss Ltd., Harrogate, England)	6 smokers
**Rossi (1)**	2017	PS (unicenter)	Italy / Private practice	20 (7/13)	NM (55)	Delayed (2 mo)	Mx, Md	SLActive (Straumann, Basel, Switzerland)	7 heavy smokers
**Rossi (2)**	2018	PS (unicenter)	Italy / Private practice	35 (13/22)	28-70 (51)	Delayed (6 wk)	Mx, Md	SLActive (Straumann, Basel, Switzerland)	10 heavy smokers
**Salman**	2019	RCT (unicenter)	USA / University	30 (18/12)	53-85 (66)	Immediate, delayed (3 mo)	Md	Osseospeed TX (Astra Tech, Mölndal, Sweden)	4 light smokers
**Sánchez-Pérez**	2007	RA (unicenter)	Spain / Private practice	66 (NM)	15-71 (43.4)	Delayed (2 mo)	Mx, Md	Bis (Biotech, Avignon, France)	40 light, heavy, and extra-heavy smokers
**Sanna**	2007	RA (unicenter)	Belgium / University	30 (18/12)	38-74 (56)	Immediate	Mx, Md	NM (Nobel Biocare, Göteborg, Sweden)	13 smokers
**Sanz-Martin**	2017	RCT (unicenter)	Spain / University	47 (21/26)	NM (58)	Delayed (12 mo)	Mx, Md	NM (Sweden & Martina, Due Carrare, Italy)	10 light smokers<br>1 former smoker
**Sayadoust**	2013	RA (unicenter)	Sweden / Public institution	80 (40/40)	NM (53.5-63.2)	Delayed (3-4 mo)	Mx, Md	Brånemark, TiUnite (Nobel Biocare, Göteborg, Sweden)	40 heavy smokers

**Schlee**	2015	PS (multicenter)	Netherlands, Germany, Italy, France, Spain / Private practice	105 (50/55)	22-77 (55.2)	NM	Mx, Md	Trabecular Meta Material (Zimmer TMT, Parsippany, USA)	17 light and heavy smokers
**Schmid (1)**	2020	RA (unicenter)	Switzerland / University	26 (12/14)	NM (72.2)	Delayed (3-6 mo)	Mx, Md	SLA (Straumann, Basel, Switzerland)	4 smokers
**Schmid (2)**	2021	RA (unicenter)	Switzerland / University	21 (9/12)	45-89 (71.4)	Delayed (3-6 mo)	Mx, Md	SLA (Straumann, Basel, Switzerland)	1 smoker
**Schneider**	2012	RA (unicenter)	Switzerland / University	70 (27/43)	19.8-76.6 (50.7)	Early, delayed (mean 12 mo)	Mx, Md	Brånemark (Nobel Biocare, Göteborg, Sweden), SLA, ITI, (Straumann, Waldenburg, Switzerland)	31 smokers
**Schoenbaum**	2021	RA (multicenter)	USA / Private practice	378 (181/197)	NM (60)	NM	Mx, Md	NM	15% of the implants in smokers and former smokers
**Schwartz-Arad (1)**	2000	RA (unicenter)	Israel / University	43 (16/27)	17-73 (47)	NM	Mx, Md	NM	NM
**Schwartz-Arad (2)**	2012	RA (unicenter)	Israel / University	261 (NM)	18-67 (48)	NM	Mx, Md	NM	89 light and heavy smokers
**Schwartz-Arad (3)**	2016	RA (unicenter)	Israel / NM	214 (35/179)	NM (50.3)	Delayed (4-6 mo)	Mx, Md	Several (Screw-Vent and Spline, Zimmer Dental Inc., Warsaw, USA; NobelActive and Replace Select, Nobel Biocare, Göteborg, Sweden; Implant Direct, Implant Direct LLC, Zurich, Switzerland)	39 smokers
**Shibuya**	2012	RA (unicenter)	Japan / University	9 (8/1)	22-71 (50.6)	NM	Mx	TiUnite (Nobel Biocare, Göteborg, Sweden)	5 smokers
**Si (1)**	2016	RA (unicenter)	China / University	80 (43/37)	25-70 (48.8)	Delayed (3-4 mo)	Mx	NM (Straumann, Basel, Switzerland)	NM
**Si (2)**	2019	RA (unicenter)	China / University	156 (56/100)	21-82 (51.5)	Delayed (3 mo)	Mx, Md	SLA (Straumann, Basel, Switzerland)	13 smokers
**Sicilia**	2021	RA (unicenter)	Spain / Private practice	268 (NM)	NM	Delayed (8-10 wk)	Md	Turned (Lifecore Biomedical), Osseotite (Biomet 3i), TiUnite (Nobel Biocare, Göteborg, Sweden)	75 smokers
**Siebers**	2010	CCT (unicenter)	Germany / Private practice	76 (34/42)	22-85 (52 ± 13)	Immediate, delayed (4-6 mo)	Mx, Md	Camlog Rootline and Screw Line (Camlog Biotechnologies, Basel, Switzerland), Osseotite (Biomet 3i, Palm Beach Gardens, USA), Restore RBM (Lifecore Biomedical, Chaska, USA)	15 smokers

**Simons**	2015	RA (unicenter)	Belgium / University	185 (69/116)	20-88 (56.4)	Delayed (3-6 mo)	Md	Brånemark MK III (Nobel Biocare, Göteborg, Sweden)	29 smokers
**Sivolella**	2020	RCT (unicenter)	Italy / University	16 (10/6)	NM (53.5)	Delayed (4 mo)	Mx, Md	Osseotite Tapered Certain Prevail (Biomet 3i)	3 light smokers
**Souza**	2019	RA (unicenter)	Brazil / NM	10 (4/6)	49-70 (60)	Delayed (6 mo)	Mx	Master Porous (Conexão Sistemas de Prótese Ltda, São Paulo, Brazil)	1 smoker
**Stacchi**	2021	RA (multicenter)	Italy / Private practice	156 (61/95)	22-86 (60.9)	NM	Mx	NM	29 smokers
**Stoker**	2012	RCT (unicenter)	Netherlands / University	94 (28/66)	NM (59.8)	Delayed (3 mo)	Md	ITI/Bonefit (Straumann, Basel, Switzerland)	35 smokers
**Striezel**	2011	RA (unicenter)	Germany, Israel / Private practice	25 (12/13)	45-74 (55.2)	Immediate	Mx, Md	Alpha Bio (Alpha-Bio Tech Ltd., Petach Tikva, Israel)	3 smokers
**Sverzut**	2008	RA (unicenter)	Brazil / University	650 (NM)	13-84 (42.7)	NM	Mx, Md	NM	76 smokers
**Tallarico (1)**	2016	RA (NM)	NM	56 (25/31)	NM (66.2)	Immediate, delayed	Mx, Md	NobelReplace Conical Connection, NobelSpeedy Groovy, Brånemark MKIII, NobelReplace Tapered Groovy (Nobel Biocare, Göteborg, Sweden)	3 light smokers
**Tallarico (2)**	2017	RA (multicenter)	Italy / Private practice	141 (49/92)	29-88 (53.3)	Immediate, delayed	Mx, Md	TiUnite Nobel Replace Conical Connection, Nobel Replace Select Tapered, NobelSpeedy Groovy (Nobel Biocare, Göteborg, Sweden)	Light and heavy smokers
**Tallarico (3)**	2018	RCT (multicenter)	Italy / Private practice	20 (10/10)	28-84 (61-67)	Delayed (4 mo)	Mx, Md	NobelSpeedy Groovy (Nobel Biocare, Göteborg, Sweden)	1 heavy smoker
**Tartaglia**	2016	RA (unicenter)	Italy / Private practice	113 (53/60)	42-90 (65)	Immediate	Mx, Md	Milde (Titanmed, Bergamo, Italy)	18 smokers
**Taschieri**	2018	RCT (unicenter)	Italy / University	52 (22/30)	31-77 (52)	Delayed (5-7 mo)	Mx	Interna, Universal Platform and Universal Plus Platform (BTI Biotechnology Institute, Vitoria-Gasteiz, Spain)	12 light and heavy smokers
**Tattan**	2021	RA (unicenter)	USA / University	201 (95/106)	45-69 (60)	NM	Mx, Md	NM	37 smokers
**Tawil**	2008	CCT (unicenter)	Lebanon / Private practice	90 (57/33)	43-84 (64.7)	Immediate, delayed	Mx, Md	Brånemark, TiUnite (Nobel Biocare, Göteborg, Sweden)	40 smokers
**Tealdo**	2015	RA (unicenter)	Italy / University	4 (3/1)	41-65 (54)	Immediate	Md	NM (Nobel Biocare, Göteborg, Sweden)	2 smokers
**Temmerman**	2015	PS (unicenter)	Belgium / University	28 (3/25)	42-76 (63)	Delayed (mean 3.6 mo)	Mx, Md	NM (Astra Tech, Dentsply Implants, Mölndal, Sweden)	Light and heavy smokers

**Testori (1)**	2001	PS (multicenter)	Italy, Canada, Ireland, USA / Private Practice	181 (76/105)	18-86 (55.4)	Delayed (4-6 mo)	Mx, Md	Osseotite NanoTite Tapered (Biomet 3i, Palm Beach Gardens, USA)	37 light smokers
**Testori (2)**	2014	RA (unicenter)	Italy / University	80 (38/42)	NM (59)	Immediate, delayed	Mx, Md	NM (Biomet 3i, Palm Beach Gardens, USA)	11 light smokers<br>15 heavy smokers
**Testori (3)**	2017	RA (multicenter)	Italy / NM	27 (8/19)	39-69 (57)	Immediate	Mx	NM (Biomet 3i, Palm Beach Gardens, USA)	3 light smokers
**Testori (4)**	2017	RA (unicenter)	Italy / Private practice	21 (11/10)	24-73 (55.6)	Immediate, delayed	Mx, Md	T3 (Biomet 3i, Palm Beach Gardens, USA)	1 light smoker<br>1 heavy smoker<br>1 pipe smoker
**Thoma (1)**	2018	RCT (multicenter)	Switzerland, Poland, Austria, Spain, USA / University + Private practice	101 (49/52)	20-75 (50.5)	Delayed (5 mo)	Mx	Osseospeed (Astra Tech, Mölndal, Sweden)	NM
**Thoma (2)**	2019	RA (unicenter)	Switzerland / University	38 (18/20)	30-92 (62.8)	NM	Mx, Md	NM (Nobel Biocare, Göteborg, Sweden)<br>NM (Straumann, Basel, Switzerland)	2 light smokers
**Thoma (3)**	2020	RCT (unicenter)	Switzerland / University	20 (7/13)	NM (44)	Delayed (3 mo)	Mx, Md	NM	2 smokers
**Troiano**	2021	RA (unicenter)	Italy / Private practice	109 (63/46)	NM (58.1)	Immediate, Delayed (2-4 mo)	Mx, Md	Several (3i, JD Dental Care, Astra Tech, Sweden & Martina, SIN, Henry Schein Krugg, Biosfera)	31 smokers
**Twito**	2014	RA (unicenter)	Israel / Military clinic	NM	22-55 (41)	Immediate, delayed	Mx, Md	NM	NM
**Urdaneta**	2012	RA (unicenter)	USA / Private practice	291 (147/144)	NM	NM	Mx, Md	Integra-CP (Bicon, Boston, USA)	22 smokers
**Uribarri**	2017	CCT (unicenter)	Spain / University	104 (44/60)	38-78 (52)	Delayed (6 mo)	Mx	Osseospeed (Astra Tech, Mölndal, Sweden)<br>SLActive (Straumann, Basel, Switzerland)	12 smokers
**Van Steenberghe**	2004	PS (unicenter)	Belgium / University	50 (25/25)	45-80 (56.5)	Immediate	Md	Brånemark (Nobel Biocare, Göteborg, Sweden)	13 heavy smokers
**Vandeweghe (1)**	2011	RA (multicenter)	Belgium / Private practice	329 (141/188)	18-84 (54)	Immediate, delayed	Mx, Md	NM (Southern Implants, Irene, South Africa)	41 smokers

**Vandeweghe (2)**	2012	RA (multicenter)	South Africa, UK, Germany / Private practice	75 (31/44)	25-82 (58)	Immediate, delayed (3 mo)	Mx, Md	Sandblasted (Max Southern Implants, Irene, South Africa)	10 light and heavy smokers
**Vandeweghe (3)**	2013	RA (multicenter)	Greece, Belgium / Private practice, University	38 (16/22)	20-82 (49)	Immediate	Mx, Md	Sandblasted (Southern Implants, Irene, South Africa)	5 smokers
**Vervaeke**	2015	RA (unicenter)	Belgium / Private Practice	376 (166/210)	17-82 (56)	Immediate, delayed	Mx, Md	Osseospeed (Astra Tech, Mölndal, Sweden)	NM
**Waechter**	2017	RCT (unicenter)	Brazil / University	20 (7/13)	NM (50.8)	Delayed (3 mo)	Md	Duo, Integra (Signo Vinces)	2 light smokers
**Wagenberg (1)**	2006	RA (unicenter)	USA / University	891 (281/510)	14-94 (57.9)	Immediate, delayed (3-6 mo)	Mx, Md	Brånemark (Nobel Biocare, Göteborg, Sweden), Osseotite (3i Implant Innovations, Palm Beach Gardens, USA)	NM
**Wagenberg (2)**	2013	RA (unicenter)	USA / Private practice	541 (NM)	12-88 (58)	Immediate, delayed	Mx, Md	NM	Heavy smokers
**Wallace**	2000	RA (unicenter)	UK / NM	56 (29/27)	NM	NM	Mx, Md	NM	17 smokers
**Wang (1)**	2016	RA (unicenter)	China / University	26 (15/11)	55-76 (64)	Delayed (3 mo)	Mx	Tissue Level (Straumann, Basel, Switzerland)	8 light smokers
**Wang (2)**	2020	RCT (unicenter)	USA / University	49 (15/34)	25-70 (46.8)	Immediate, Delayed (3 mo)	Mx, Md	NobelParallel Conical Connection (Nobel Biocare, Göteborg, Sweden)	1 light smoker
**Wennström m**	2004	RCT (unicenter)	Sweden / University	51 (20/31)	36-80 (59.5)	Delayed (3-6 mo)	Mx, Md	Tioblast (Astra Tech, Mölndal, Sweden)	17 smokers
**Werbelow**	2020	RA (unicenter)	Germany / Private practice	23 (13/10)	42-74 (64)	Immediate	Mx, Md	blueSKY© (Bredent GmbH & Co. KG, Senden, Germany)	2 smokers
**Widmark**	2001	RA (unicenter)	Sweden / Public Service	36 (13/23)	NM	6-8 months	Mx	Brånemark (Nobel Biocare, Göteborg, Sweden)	11 smokers
**Windael (1)**	2018	PS (unicenter)	Belgium / University	21 (8/13)	49-84 (68.4)	Immediate	Md	Osseospeed (Astra Tech, Mölndal, Sweden)	1 smoker<br>6 former smokers
**Windael (2)**	2021	RA (unicenter)	Belgium / Private practice	407 (186/221)	28-92 (64.9)	Immediate, Delayed (3 mo)	Mx, Md	Osseospeed (Astra Tech, Mölndal, Sweden)	50 smokers<br>66 former smokers
**Wolf**	2021	RA (unicenter)	Germany / University	121 (91/30)	45-89 (65.7)	NM	Mx, Md	Several (Dentsply Sirona, Straumann, Camlog, Nobel Biocare)	27 smokers

**Wu (1)**	2014	RA (unicenter)	Canada / Private practice	490 (198/292)	17-93 (56.4)	Immediate, delayed (mean 5.1 mo)	Mx, Md	Brånemark (Nobel Biocare, Göteborg, Sweden)	Heavy smokers
**Wu (2)**	2016	RA (unicenter)	Canada / Private practice	728 (375/353)	18-93 (57.7)	Immediate, Delayed	Mx, Md	TiUnite (Nobel Biocare, Göteborg, Sweden)	83 smokers
**Wu (3)**	2017	RA (unicenter)	Canada / University	799 (369/430)	18-93 (56.6)	NM	Mx, Md	TiUnite (Nobel Biocare, Göteborg, Sweden)	Any degree of smoking
**Wu (4)**	2018	RA (unicenter)	China / University	72 (42/30)	NM (47)	Delayed (3-6 mo)	Mx, Md	XiVE S Plus (Dentsply implants, Mölndal, Sweden)	9 light smokers
**Zafiropoulos**	2010	RA (unicenter)	Germany / Private practice	252 (135/117)	43-70 (49)	Immediate	Mx, Md	Camlog root line (Altatec, Wimsheim, Germany), SLA (Straumann, Waldenburg, Switzerland)	86 light smokers
**Zembic**	2016	PS (unicenter)	Netherlands / University	20 (15/5)	45-84 (61)	Delayed (3-5 mo)	Mx	Roxolid Tissue Level (Straumann, Basel, Switzerland)	6 smokers
**Zinser**	2013	RA (unicenter)	Netherlands / Public Hospital	224 (120/104)	35-81 (56)	Delayed (3-6 mo)	Mx	NM	NM
**Zumstein**	2019	RA (unicenter)	Switzerland / Private practice	51 (22/29)	NM (58.2)	Immediate, delayed	Mx, Md	Neoss ProActive Straight (Neoss Ltd, Harrogate, UK)	4 smokers

NM – not mentioned; RA – retrospective analysis; PS – prospective study; CCT – controlled clinical trial; RCT – randomized controlled trial
G1 – group smokers; G2 – group non-smokers
d – days; wk – weeks; mo – months; Mx – maxilla; Md – mandible
[a] light smokers <10 cig/day; heavy smokers >10 cig/day; extra-heavy smokers >20 cig/day

e. Table S2. Quality assessment of the included studies, according to the National Institutes of Health (NIH).

Study	Year	Was the study question or objective clearly stated?	Was the study population clearly and fully described, including a case definition?	Were the cases consecutive?	Were the subjects comparable?	Was the intervention clearly described?	Were the outcome measures clearly defined, valid, reliable, and implemented consistently across all study participants?	Was the length of follow-up adequate? [a]	Were the statistical methods well-described?	Were the results well-described?	Total (n/9)
Abduljabbar	2018	1	1	0	1	1	1	1	0	1	7/9
Abi-Aad	2018	1	1	0	1	1	1	1	1	1	8/9
Agliardi	2010	1	1	0	1	1	1	1	0	1	7/9
Agliardi	2014	1	1	1	1	1	1	1	1	1	9/9
Al Amri	2017	1	1	0	1	1	1	1	0	1	7/9
Al Najam	2021	1	1	0	1	1	1	1	1	1	8/9
Al-Aali	2018	1	1	0	1	1	1	1	0	1	7/9
Alahmari	2019	1	1	0	1	1	1	1	0	1	7/9
Alasqah	2019	1	1	0	1	1	1	1	0	1	7/9
Alghamdi	2020	1	1	0	1	1	1	1	0	1	7/9
Aloy-Prósper	2016	1	1	0	1	1	1	1	1	1	8/9
Alqahtani	2020	1	1	0	1	1	1	1	0	1	7/9
Alsaadi (1)	2007	1	1	1	1	1	1	1	1	1	9/9
Alsaadi (2)	2008	1	1	0	1	1	1	1	1	1	8/9
Alsaadi (3)	2008	1	1	0	1	1	1	1	1	1	8/9
Al-Sabbagh	2015	1	1	0	1	1	1	1	1	0	8/9
Angelis	2020	1	1	1	1	1	1	1	0	1	8/9
Anitua (1)	2008	1	1	1	1	1	1	1	1	1	9/9
Anitua (2)	2019	1	1	0	1	1	1	1	1	1	8/9
Anner	2010	1	1	0	1	1	1	1	0	1	7/9
Arora (1)	2018	1	1	0	1	1	1	1	1	1	8/9
Arora (2)	2018	1	1	0	1	1	1	1	1	1	8/9
Atarchi	2020	1	1	0	1	1	1	1	1	1	8/9
Aykent	2007	1	1	1	1	1	1	1	1	1	9/9

Bain (1)	1993	1	1	0	1	1	1	1	0	1	7/9
Bain (2)	1996	1	1	0	1	1	1	1	0	1	7/9
Balaguer	2015	1	1	0	1	1	1	1	0	1	7/9
Balshi	2014	1	1	1	1	1	1	1	0	1	8/9
Barbato	2018	1	1	0	1	1	1	1	0	1	7/9
Barnea	2016	1	1	1	1	1	1	1	1	1	9/9
Barone (1)	2015	1	1	1	1	1	1	1	1	1	9/9
Barone (2)	2016	1	1	1	1	1	1	1	0	1	8/9
Bell (1)	2011	1	1	0	1	1	1	1	0	1	7/9
Bell (2)	2014	1	1	1	1	1	1	1	0	1	8/9
Bischof	2006	1	1	1	1	1	1	1	1	1	9/9
Boardman	2016	1	1	0	1	1	1	1	1	1	7/9
Boboeva	2021	1	1	1	1	1	1	1	1	1	9/9
Bouhy	2020	1	1	1	1	1	1	1	1	1	9/9
Brandt	2012	1	1	0	1	1	1	1	0	0	6/9
Cakarer	2014	1	1	1	1	1	1	1	1	1	9/9
Cannizzaro (1)	2003	1	1	0	1	1	1	1	0	1	7/9
Cannizzaro (2)	2008	1	1	1	1	1	1	1	1	1	9/9
Cannizzaro (3)	2012	1	1	1	1	1	1	1	1	1	9/9
Cannizzaro (4)	2016	1	1	1	1	1	1	1	1	1	9/9
Cannizzaro (5)	2016	1	1	1	1	1	1	1	1	1	9/9
Cannizzaro (6)	2017	1	1	1	1	1	1	1	1	1	9/9
Cannizzaro (7)	2018	1	1	1	1	1	1	1	1	1	9/9
Cannizzaro (8)	2018	1	1	1	1	1	1	1	1	1	9/9
Cannizzaro (9)	2016	1	1	1	1	1	1	1	1	1	9/9
Canullo	2014	1	1	0	1	1	1	1	1	1	8/9
Cappare	2019	1	1	1	1	1	1	1	1	1	9/9
Carr	2019	1	1	1	1	1	1	1	1	1	9/9
Cavalcanti	2011	1	1	1	1	1	1	1	0	1	8/9
Cha	2014	1	1	1	1	1	1	1	1	1	9/9
Chang	2020	1	1	0	1	1	1	1	1	1	8/9
Chiapasco (1)	2018	1	1	0	1	1	1	1	1	1	8/9
Chiapasco (2)	2020	1	1	0	1	1	1	1	1	1	8/9
Chrcanovic (1)	2017	1	1	0	1	1	1	1	1	1	8/9
Chrcanovic (2)	2017	1	1	1	1	1	1	1	1	1	9/9

Chrcanovic (3)	2018	1	1	0	1	1	1	1	1	1	8/9
Clauser	2020	1	1	1	1	1	1	1	1	1	9/9
Conrad	2011	1	1	0	1	1	1	1	1	1	8/9
Corvino	2020	1	1	0	1	1	1	1	0	1	7/9
Coskunses	2021	1	1	1	1	1	1	1	1	1	9/9
Crespi	2019	1	1	0	1	1	1	1	1	1	8/9
Cristalli	2015	1	1	1	1	1	1	1	1	1	9/9
Cucchi	2019	1	1	0	1	1	1	1	1	1	8/9
Daher	2019	1	1	0	1	1	1	1	1	1	8/9
Daneshvar	2016	1	1	1	1	1	1	1	0	1	8/9
Daubert	2015	1	1	1	1	1	1	1	1	1	9/9
De Bruyn (1)	1994	1	1	0	1	1	1	1	0	1	7/9
De Bruyn (2)	1999	1	1	0	1	1	1	1	0	1	7/9
Degidi	2015	1	1	0	1	1	1	1	1	1	8/9
Deli	2014	1	1	0	1	1	1	1	0	1	7/9
D'haese	2013	1	1	0	1	1	1	1	0	1	7/9
Di Stefano	2020	1	1	0	1	1	1	1	1	1	8/9
Doan	2014	1	1	1	1	1	1	1	0	1	8/9
Donati (1)	2015	1	1	0	1	1	1	1	1	1	8/9
Donati (2)	2018	1	1	0	1	1	1	1	1	1	8/9
Donos	2019	1	1	0	1	1	1	1	0	1	7/9
Doyle	2007	1	1	0	1	1	1	1	0	1	7/9
Engstrand	2003	1	1	1	1	1	1	1	1	1	9/9
Feher	2020	1	1	0	1	1	1	1	1	1	8/9
Felice	2015	1	1	1	1	1	1	1	1	1	9/9
Flores-Guillen	2018	1	1	1	1	1	1	1	1	1	9/9
Fonseca	2021	1	1	0	1	1	1	1	1	1	8/9
Francetti (1)	2014	1	1	0	1	1	1	1	0	1	7/9
Francetti (2)	2019	1	1	0	1	1	1	1	1	1	8/9
Friberg	2008	1	1	1	1	1	1	1	1	1	9/9
Gamper	2017	1	1	1	1	1	1	1	1	1	9/9
Gander	2014	1	1	1	1	1	1	1	1	1	9/9
Gaspersic	2021	1	1	0	1	1	1	1	1	1	8/9
Geurs	2001	1	1	0	1	1	1	1	0	1	7/9
Ghazal	2019	1	1	0	1	1	1	1	1	1	8/9

Gherlone	2016	1	1	0	1	1	1	1	1	1	8/9
Gjelvold	2020	1	1	1	1	1	1	1	1	1	9/9
Gorman	1994	1	1	0	1	1	1	1	0	1	7/9
Grandi (1)	2012	1	1	1	1	1	1	1	1	1	9/9
Grandi (2)	2012	1	1	0	1	1	1	1	1	1	8/9
Grandi (3)	2012	1	1	1	1	1	1	1	1	1	9/9
Grandi (4)	2013	1	1	1	1	1	1	1	1	1	9/9
Grandi (5)	2014	1	1	0	1	1	1	1	1	1	8/9
Grandi (6)	2015	1	1	1	1	1	1	1	1	1	9/9
Grisar	2017	1	1	1	1	1	1	1	1	1	9/9
Groenendijk	2020	1	1	1	1	1	1	1	0	1	8/9
Grossmann	2008	1	1	1	1	1	1	1	0	1	8/9
Grunder	1999	1	1	0	1	1	1	1	1	1	8/9
Guarnieri (1)	2019	1	1	1	1	1	1	1	1	1	9/9
Guarnieri (2)	2020	1	1	1	1	1	1	1	1	1	9/9
Guida	2020	1	1	0	1	1	1	1	1	1	8/9
Göthberg	2018	1	1	0	1	1	1	1	1	1	8/9
Habsha	2000	1	1	1	1	1	1	1	1	1	9/9
Hakam	2021	1	1	0	1	1	1	1	1	1	8/9
Han	2018	1	1	0	1	1	1	1	1	1	8/9
Hartlev	2021	1	1	1	1	1	1	1	1	1	9/9
Hattingh (1)	2018	1	1	0	1	1	1	1	0	1	7/9
Hattingh (2)	2019	1	1	0	1	1	1	1	1	1	8/9
He	2015	1	1	1	1	1	1	1	1	1	9/9
Higuchi	2020	1	1	0	1	1	1	1	0	1	7/9
Hingsammer	2018	1	1	0	1	1	1	1	1	1	8/9
Hinze	2010	1	1	0	1	1	1	1	0	1	7/9
Hof	2014	1	1	1	1	1	1	1	1	1	9/9
Holahan	2008	1	1	0	1	1	1	1	1	1	8/9
Horwitz	2012	1	1	0	1	1	1	1	0	1	7/9
Hsu	2016	1	1	0	1	1	1	1	1	1	8/9
Hu	2020	1	1	1	1	1	1	1	1	0	8/9
Ibañez (1)	2003	1	1	0	1	1	1	1	0	1	7/9
Ibañez (2)	2005	1	1	1	1	1	1	1	0	1	8/9
Iorio-Siciliano	2016	1	1	0	1	1	1	1	0	1	7/9

Ji	2012	1	1	0	1	1	1	1	0	1	7/9
Jones	1999	1	1	0	1	1	1	1	0	1	7/9
Kan	1999	1	1	0	1	1	1	1	0	1	7/9
Karoussis	2003	1	1	0	1	1	1	1	1	1	8/9
Keller	1999	1	1	1	1	1	1	1	0	1	8/9
Khouly	2017	1	1	1	1	1	1	1	1	0	8/9
Kim	2015	1	1	0	1	1	1	1	1	1	8/9
Kinsel	2007	1	1	1	1	1	1	1	1	1	9/9
Klotz	2019	1	1	1	1	1	1	1	1	1	9/9
Kolerman	2017	1	1	1	1	1	1	1	0	1	8/9
Komiyama	2008	1	1	0	1	1	1	1	0	1	7/9
Krennmair (1)	2016	1	1	0	1	1	1	1	1	1	8/9
Krennmair (2)	2019	1	1	1	1	1	1	1	1	1	9/9
Kumar	2002	1	1	1	1	1	1	1	0	1	8/9
Lambert	2000	1	1	0	1	1	1	1	1	1	8/9
Le	2013	1	1	0	1	1	1	1	0	1	7/9
Lee (1)	2018	1	1	0	1	1	1	1	1	0	7/9
Lee (2)	2020	1	1	0	1	1	1	1	0	1	7/9
Leventi	2014	1	1	0	1	1	1	1	0	1	7/9
Levin (1)	2008	1	1	1	1	1	1	1	0	1	8/9
Levin (2)	2011	1	1	1	1	1	1	1	1	1	9/9
Lin	2012	1	1	0	1	1	1	1	0	1	7/9
Lindquist	1997	1	1	0	1	1	1	1	1	1	8/9
Lini	2019	1	1	1	1	1	1	1	1	0	8/9
Lobato	2020	1	1	0	1	1	1	1	1	0	7/9
Locante	2004	1	1	0	1	1	1	1	0	1	7/9
Lombardo	2020	1	1	1	1	1	1	1	1	1	9/9
Luongo	2020	1	1	0	1	1	1	1	0	1	7/9
Malchiodi (1)	2014	1	1	1	1	1	1	1	1	1	9/9
Malchiodi (2)	2016	1	1	0	1	1	1	1	1	1	8/9
Maló (1)	2013	1	1	1	1	1	1	1	0	1	8/9
Maló (2)	2019	1	1	1	1	1	1	1	1	1	9/9
Maló (3)	2019	1	1	0	1	1	1	1	1	1	8/9
Mastrangelo	2018	1	1	0	1	1	1	1	1	1	8/9
Mayfield	2001	1	1	0	1	1	1	1	0	1	7/9

McCarthy	2003	1	1	1	1	1	1	1	0	1	8/9
Mendonca	2014	1	1	0	1	1	1	1	1	1	8/9
Merli (1)	2017	1	1	0	1	1	1	1	1	1	8/9
Merli (2)	2020	1	1	1	1	1	1	1	1	1	9/9
Mertens	2012	1	1	1	1	1	1	1	1	1	9/9
Mijiritsky (1)	2013	1	1	1	1	1	1	1	0	1	8/9
Mijiritsky (2)	2015	1	1	1	1	1	1	1	0	1	8/9
Mijiritsky (3)	2016	1	1	0	1	1	1	1	0	1	7/9
Minsk	1996	1	1	1	1	1	1	1	0	1	8/9
Mir-Mari	2012	1	1	0	1	1	1	1	0	1	7/9
Moheng	2005	1	1	1	1	1	1	1	1	1	9/9
Moraguez	2016	1	1	0	1	1	1	1	1	1	8/9
Morales-Vadillo	2013	1	1	1	1	1	1	1	0	1	8/9
Mordenfeld	2014	1	1	1	1	1	1	1	1	1	9/9
Mundt	2006	1	1	1	1	1	1	1	1	1	9/9
Munoz	2021	1	1	0	1	1	1	1	1	1	8/9
Naeini	2018	1	1	0	1	1	1	1	1	1	8/9
Nedir	2004	1	1	1	1	1	1	1	0	1	8/9
Niedemaier	2017	1	1	0	1	1	1	1	1	1	8/9
Nitzan	2005	1	1	1	1	1	1	1	0	1	8/9
Noelken	2020	1	1	0	1	1	1	1	1	1	8/9
Nogueira	2018	1	1	1	1	1	1	1	1	1	9/9
Noguerol	2006	1	1	1	1	1	1	1	1	1	9/9
Norton	2017	1	1	1	1	1	1	1	1	1	9/9
Nyström	2009	1	1	1	1	1	1	1	1	1	9/9
Oliva	2012	1	1	0	1	1	1	1	0	1	7/9
Olmedo-Gaya	2016	1	1	1	1	1	1	1	1	1	9/9
Olson	2000	1	1	0	1	1	1	1	1	1	8/9
Omran	2015	1	1	1	1	1	1	1	1	1	9/9
Park	2019	1	1	0	1	1	1	1	1	1	8/9
Patil	2020	1	1	0	1	1	1	1	0	1	7/9
Peleg	2006	1	1	0	1	1	1	1	0	1	7/9
Penarrocha	2014	1	1	0	1	1	1	1	0	1	7/9

Penarrocha-Oltra	2019	1	1	1	1	1	1	1	1	1	9/9
Perez	2020	1	1	0	1	1	1	1	1	1	8/9
Pico	2019	1	1	1	1	1	1	1	1	1	9/9
Pozzi (1)	2014	1	1	1	1	1	1	1	1	1	9/9
Pozzi (2)	2014	1	1	1	1	1	1	1	1	1	9/9
Pozzi (3)	2015	1	1	1	1	1	1	1	1	1	9/9
Pozzi (4)	2016	1	1	1	1	1	1	1	1	1	9/9
Prati	2020	1	1	1	1	1	1	1	1	1	9/9
Queridinha	2016	1	1	0	1	1	1	1	1	1	8/9
Raabe	2021	1	1	0	1	1	1	1	1	1	8/9
Raes (1)	2015	1	1	1	1	1	1	1	1	1	9/9
Raes (2)	2018	1	1	0	1	1	1	1	1	1	8/9
Ramaglia	2015	1	1	0	1	1	1	1	0	1	7/9
Rasperini	2014	1	1	1	1	1	1	1	0	1	8/9
Ravida (1)	2018	1	1	0	1	1	1	1	1	1	8/9
Ravida (2)	2019	1	1	1	1	1	1	1	1	1	9/9
Roccuzzo	2016	1	1	1	1	1	1	1	1	1	9/9
Rodriguez-Argueta	2011	1	1	1	1	1	1	1	0	1	8/9
Romandini	2019	1	1	0	1	1	1	1	0	1	7/9
Romanos (1)	2013	1	1	0	1	1	1	1	0	1	7/9
Romanos (2)	2014	1	1	0	1	1	1	1	0	1	7/9
Rosen (1)	1999	1	1	1	1	1	1	1	0	1	8/9
Rosen (2)	2018	1	1	1	1	1	1	1	1	1	9/9
Rossi (1)	2017	1	1	1	1	1	1	1	0	1	8/9
Rossi (2)	2018	1	1	1	1	1	1	1	0	1	8/9
Salman	2019	1	1	0	1	1	1	1	1	1	8/9
Sánchez-Pérez	2007	1	1	1	1	1	1	1	0	1	8/9
Sanna	2007	1	1	1	1	1	1	1	1	1	9/9
Sanz-Martin	2017	1	1	1	1	1	1	1	1	1	9/9
Sayardoust	2013	1	1	1	1	1	1	1	0	1	8/9
Schlee	2015	1	1	0	1	1	1	1	0	1	7/9
Schmid (1)	2020	1	1	0	1	1	1	1	1	1	8/9
Schmid (2)	2021	1	1	0	1	1	1	1	1	1	8/9

Schneider	2012	1	1	1	1	1	1	1	1	1	9/9
Schoenbaum	2021	1	1	0	1	1	1	1	1	1	8/9
Schwartz-Arad (1)	2000	1	1	0	1	1	1	1	0	1	7/9
Schwartz-Arad (2)	2002	1	1	0	1	1	1	1	0	1	7/9
Schwartz-Arad (3)	2016	1	1	1	1	1	1	1	1	1	9/9
Shibuya	2012	1	1	0	1	1	1	1	0	1	7/9
Si (1)	2016	1	1	1	1	1	1	1	1	1	9/9
Si (2)	2019	1	1	1	1	1	1	1	1	1	9/9
Siebers	2010	1	1	0	1	1	1	1	0	1	7/9
Simons	2015	1	1	0	1	1	1	1	1	1	8/9
Sivolella	2020	1	1	0	1	1	1	1	1	1	8/9
Souza	2019	1	1	0	1	1	1	1	0	1	7/9
Stacchi	2021	1	1	1	1	1	1	1	1	1	9/9
Stoker	2012	1	1	0	1	1	1	1	0	1	7/9
Strietzel	2011	1	1	0	1	1	1	1	1	1	8/9
Sverzut	2008	1	1	1	1	1	1	1	1	1	9/9
Tallarico (1)	2016	1	1	1	1	1	1	1	1	1	9/9
Tallarico (2)	2017	1	1	1	1	1	1	1	1	1	9/9
Tallarico (3)	2018	1	1	1	1	1	1	1	1	0	8/9
Tartaglia	2016	1	1	0	1	1	1	1	1	1	8/9
Taschieri	2018	1	1	0	1	1	1	1	1	1	8/9
Tattan	2021	1	1	1	1	1	1	1	1	1	9/9
Tawil	2008	1	1	1	1	1	1	1	1	1	9/9
Tealdo	2015	1	1	0	1	1	1	1	0	1	7/9
Temmerman	2015	1	1	0	1	1	1	1	1	1	8/9
Testori (1)	2001	1	1	1	1	1	1	1	0	1	8/9
Testori (2)	2014	1	1	1	1	1	1	1	1	1	9/9
Testori (3)	2017	1	1	0	1	1	1	1	0	1	7/9
Testori (4)	2017	1	1	0	1	1	1	1	1	1	8/9
Thoma (1)	2018	1	1	0	1	1	1	1	1	1	8/9
Thoma (2)	2019	1	1	0	1	1	1	1	0	1	7/9
Thoma (3)	2020	1	1	0	1	1	1	1	1	1	8/9

Troiano	2021	1	1	0	1	1	1	1	1	1	8/9
Twito	2014	1	1	1	1	1	1	1	1	1	9/9
Urdaneta	2012	1	1	0	1	1	1	1	1	1	8/9
Uribarri	2017	1	1	1	1	1	1	1	1	1	9/9
van Steenberghe	2004	1	1	1	1	1	1	1	1	1	9/9
Vandeweghe (1)	2011	1	1	0	1	1	1	1	1	1	8/9
Vandeweghe (2)	2012	1	1	1	1	1	1	1	0	1	8/9
Vandeweghe (3)	2013	1	1	1	1	1	1	1	0	1	8/9
Vervaeke	2015	1	1	1	1	1	1	1	1	1	9/9
Waechter	2017	1	1	0	1	1	1	1	1	1	8/9
Wagenberg (1)	2006	1	1	1	1	1	1	1	0	1	8/9
Wagenberg (2)	2013	1	1	1	1	1	1	1	0	1	8/9
Wallace	2000	1	1	0	1	1	1	1	0	1	7/9
Wang (1)	2016	1	1	0	1	1	1	1	0	1	7/9
Wang (2)	2020	1	1	0	1	1	1	1	1	1	8/9
Wennström	2004	1	1	0	1	1	1	1	1	1	8/9
Werbelow	2020	1	1	1	1	1	1	1	1	1	9/9
Widmark	2001	1	1	0	1	1	1	1	0	1	7/9
Windael (1)	2018	1	1	1	1	1	1	1	1	1	9/9
Windael (2)	2021	1	1	1	1	1	1	1	1	1	9/9
Wolf	2021	1	1	0	1	1	1	1	0	1	7/9
Wu (1)	2014	1	1	0	1	1	1	1	1	1	8/9
Wu (2)	2016	1	1	0	1	1	1	1	1	1	8/9
Wu (3)	2017	1	1	0	1	1	1	1	1	1	8/9
Wu (4)	2018	1	1	0	1	1	1	1	1	1	8/9
Zafiropoulos	2010	1	1	0	1	1	1	1	1	1	8/9
Zembic	2010	1	1	1	1	1	1	1	1	1	9/9
Zinser	2013	1	1	1	1	1	1	1	0	1	8/9
Zumstein	2019	1	1	1	1	1	1	1	1	1	9/9

[a] 3 months of follow-up was considered to be of adequate length.
Color codes concerning risk of bias of the studies (last column of the table): low (green), moderate (yellow) or high (red) risk of bias
